# Molybdenum trioxide as a newer diversified economic catalyst for the transformation of nitroarenes to arylamine and 5-substituted-1*H*-tetrazole†

**DOI:** 10.1039/d4ra05443a

**Published:** 2024-09-18

**Authors:** Anand Maurya, Upendra Kumar Patel, Sanjeev Kumar, Alka Agarwal

**Affiliations:** a Department of Medicinal Chemistry, Institute of Medical Sciences, Banaras Hindu University Varanasi-221005 Uttar Pradesh India agarwal.dralka@gmail.com

## Abstract

The present work has developed a straightforward, gentle, and effective approach for synthesizing arylamines and 5-substituted-1*H*-tetrazole derivatives, and among the two tested catalysts, molybdenum trioxide (MoO_3_) proved to be highly effective. The selective hydrogenation of nitroarenes to arylamines presents a significant challenge due to the complex reaction mechanism and the competitive hydrogenation of other reducible functional groups. It facilitated the transfer hydrogenation of nitrobenzene using hydrazine hydrate-produced amino compounds and enabled the [3 + 2] cycloaddition of sodium azide with aromatic nitriles to yield 5-substituted-1*H*-tetrazoles. The structure of compound 5-(4-bromophenyl)-1*H*-tetrazole (5k) was verified through single-crystal X-ray analysis, and the calculation of Green Chemistry Metrics showed the optimal range. Notably, the MoO_3_ catalyst can be reutilized for up to seven cycles with minimal loss of effectiveness. These attributes make molybdenum trioxide particularly attractive for industrial applications. This methodology offers several advantages over traditional synthetic methods.

## Abstract 2

The present work has developed a straightforward, gentle, and effective approach for synthesizing arylamines and 5-substituted-1*H*-tetrazole derivatives, and among the two tested catalysts, molybdenum trioxide (MoO_3_) proved to be highly effective.

## Graphical abstract

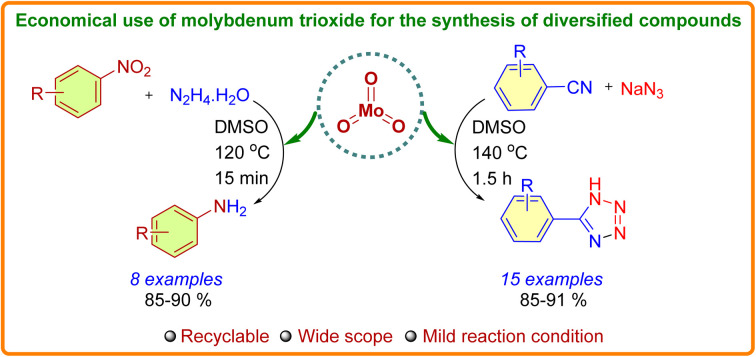

## 1 Introduction

Catalysis is integral to numerous chemical processes, serving as the foundation for countless synthetic transformations in research and industrial contexts. Numerous catalytic reagents can significantly enhance the reaction yield and selectivity while allowing for more easily controlled reaction conditions.^1^ Significant advancements have been made in heterogeneous and homogeneous catalysis over the twentieth century. Heterogeneous catalysis offers the advantage of easy catalyst separation at the end of the reaction, while homogeneous catalysis is known for achieving higher catalytic activity.^2^ Transition metal oxides have been extensively explored for their applications in various environmental, industrial, and technological fields. Transition metal catalysts were first demonstrated by Busch in 1929, who used Pd/CaCO_3_,^3^ and later Pietra,^4^ who used Pd/C for the hydrazine-facilitated transfer hydrogenation (TH) of nitro-aromatics. Catalytic transfer hydrogenation (CTH) presents an appealing alternative for converting nitroarenes to anilines. It involves the addition of hydrogen from a hydrogen donor compound such as hydrazine,^5^ formic acid,^6^ isopropanol,^7^ or sodium borohydride (NaBH_4_),^8^ to an organic molecule, and has recently become a significant research focus.^9^

A recent study has investigated molybdenum oxide-based catalysts for this reaction.^10,11^ Studies have suggested that reactive hydrogen species, in the forms of H^*δ*−^ and H^*δ*+^, are active in reducing nitrobenzene when using MoO_*x*_ catalysts.^10^ These hydrogen species are believed to form through hydrazine (N_2_H_4_) activation, particularly at low oxidation state molybdenum sites, such as Mo^4+^ and Mo^3+^, which serve as adsorption sites.^10,11^

Molybdenum oxides (MoO_*x*_) have demonstrated potential as catalysts due to their various oxidation states, including Mo^4+^, Mo^5+^, and Mo^6+^. They have been utilized in numerous applications such as CO_2_ hydrogenation, water splitting,^12–14^ and the reduction of nitrobenzene to aniline. The most effective active sites for the hydrogenation of nitrobenzene are reported to be the acidic sites associated with the low oxidation states of molybdenum, such as Mo^5+^, Mo^4+^, and Mo^3+^. This aligns with observations that molybdenum trioxide (MoO_3_) has significantly inferior performance to MoO_*x*_^10,15^ due to the presence of Mo^6+^. Molybdenum trioxide (MoO_3_) can exist in various phases, including α-(orthorhombic), β-(monoclinic), and h-(hexagonal) forms while MoO_3_ primarily consists of less active Mo^6+^ species; these can be reduced to lower oxidation states, altering the catalyst's structure. The *in situ* reduction of Mo^6+^ during catalytic reactions has been reported, namely, dehydroaromatization in methane using Mo-loaded HZSM-5 ^16,17^ and MoO_3_/HMCM-49.^18^ The reduction of Mo^6+^ by CH_4_ leads to the formation of Mo_2_C, which is proposed to be the active phase for this reaction.^19^ This process can be observed in the reaction profiles as an induction period.^17^ Recent studies have demonstrated that Mo^6+^ can be reduced to Mo^5+^ or Mo^4+^ in the presence of hydrazine.^20^ The formation of Mo^4+^ is significant as it is proposed to be the active site for decomposing N_2_H_4_ into reactive hydrogen species.^10^ This reduction has been observed during the hydrogenation of nitrobenzene to aniline over [FeMo]S_*x*_ using N_2_H_4_, forming Mo^4+^.^15^

**Fig. 1:**
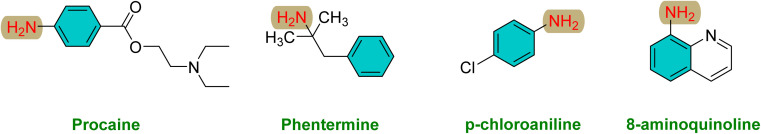
Structures of the marketed drugs contain an amine functional group.

According to literature reports, aromatic amines serve as essential components in synthesizing pharmaceuticals, dyes, pigments, polymers, and agrochemicals.^21,22^ It is also observed that nearly one-fourth of all pharmaceutical amine-containing drugs, such as procaine and phentermine, are widely used (Fig. 1).^23,24^

These drugs are clinically used for the treatment of local anesthetics and treat conditions like attention deficit hyperactivity disorder (ADHD) and narcolepsy, and the reduction of *p*-chloronitrobenzene and 8-nitroquinoline into *p*-chloroaniline and 8-aminoquinoline, respectively. These compounds serve as essential intermediates in the synthesis of paludrine and primaquine, which are important antimalarial drugs (Fig. 1).^25^

Several methods suffer from drawbacks in synthesizing anilines from nitrobenzene, including harsh reaction conditions, low yields, high temperatures, long reaction times, and costly reagents. Additionally, the catalysts used can be toxic, less accessible, thermally unstable, and exhibit poor functional group tolerance, often leading to side product formation. The preparation and use of these reagents also demand strict conditions. Therefore, significant room remains for improving and developing new, simple, and efficient reagents to address and mitigate these issues.^26,27^ Following research into metal oxide catalysts, we found that molybdenum trioxide (MoO_3_) is exceptionally effective at stabilizing hydride and proton when using hydrazine as a hydrogen source.

Tetrazoles are heterocyclic compounds comprising a five-membered ring of one carbon atom and four nitrogen atoms. These compounds can exist in two tautomeric forms: 1*H*-tetrazole and 2*H*-tetrazoles. In solution, the 1*H*-tetrazole form is more stable and commonly found, while the 2*H*-tetrazole form is more stable in the gas phase.^28^ Tetrazoles possess a wide array of physicochemical characteristics such as a high dipole moment, elevated formation enthalpy, robust stability, as well as notable acidity, basicity and exhibit diverse biological properties, including coordination chemistry, materials science, medicinal/biological sciences, and catalysis.^28–33^

**Fig. 2:**
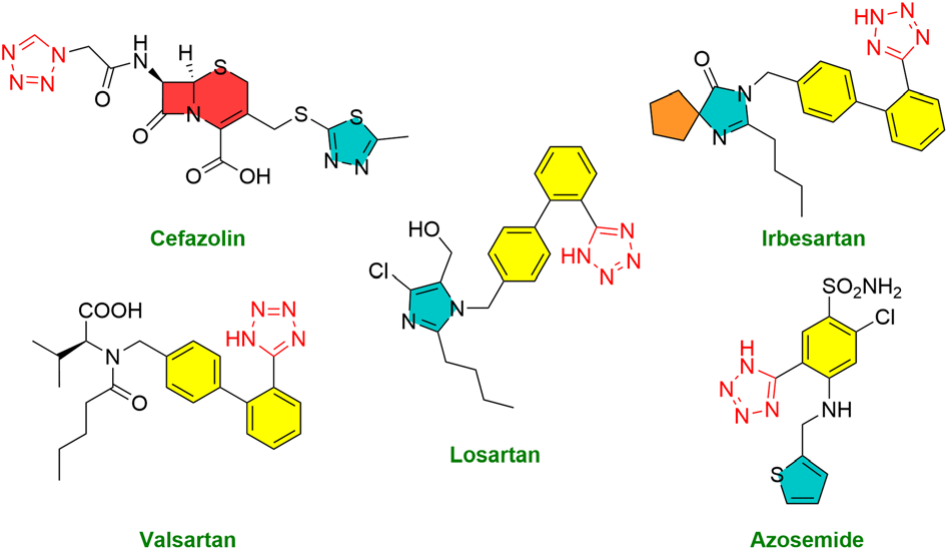
Structure of marketed drugs containing the tetrazole moiety.

Medications incorporating tetrazole compounds are employed in diverse therapeutic areas, encompassing antibacterial,^34^ antifungal,^35^ anticancer,^36^ antitubercular,^37^ peptides inhibitor,^38^ and antimalarial treatments.^39^ The tetrazole moiety is a constituent in several widely utilized drugs, such as losartan, valsartan, cefazolin, irbesartan, and azosemide (Fig. 2).^40^

Due to their excellent adaptability and broad utility, there has been significant interest in exploring the catalytic synthesis of tetrazole derivatives. Due to their widespread applications, 5-substituted tetrazoles have been a focal point in fundamental research.^41,42^ Previous research on various catalytic systems has enabled scientists to develop methods containing transition metals with precisely defined sizes, which are known as outstanding catalysts.

Keeping the above facts and applications in mind, we made efforts to explore the catalytic use or property of MoO_3_ in the synthesis of diversified compounds hydrazine-mediated transfer hydrogenation of nitroarenes to arylamines and synthesis of 5-substituted-1*H*-tetrazoles. The synthesized arylamines and 5-substituted-1*H*-tetrazoles were characterized by NMR, IR, and HRMS, and a single-crystal XRD study was done for compound 5-(4-bromophenyl)-1*H*-tetrazole (5k).

## 2 Experimental

### 2.1 Materials and methods

All the reagents and solvents were acquired from E. Merck (India), Avra, CDH, and Sigma-Aldrich and were used directly without additional processing. The reactions were monitored using thin-layer chromatography (TLC) on a pre-coated silica gel 60 F254 mesh. The results were observed using UV light or an iodine chamber. Merck silica gel (230–400 mesh) was employed for column chromatography. The compounds melting points were ascertained using the open capillary method, the results of which were uncorrected. The NMR spectra were collected on a Bruker Ascend 600 MHz spectrophotometer operating at 600 MHz for ^1^H and 151 MHz for ^13^C experiments. The chemical shifts are reported on a ppm scale concerning CDCl_3_ (7.269 ppm) for ^1^H and (77.00 ppm) for ^13^C NMR and DMSO-d_6_ (2.5 ppm), ^1^H (3.5 ppm) for moisture, and (40.39 ppm) for ^13^C NMR as an internal standard. The abbreviations are s = singlet, d = doublet, t = triplet, q = quartet, dd = double doublet, and m = multiplet. The chemical shifts were gauged in parts per million (ppm) on the delta (*δ*) scale with tetramethylsilane (TMS) acting as the internal reference. The mass spectra were recorded on a Sciex X500R QTOF mass spectrometer. FT-IR spectra were recorded in KBr pellets in the 4000–400 cm^−1^ range at room temperature using a PerkinElmer400 FT-IR spectrometer. The single crystal X-ray analysis further verified the synthesized tetrazole compound 5k. Crystals were formed *via* the slow evaporation of the solution with methanol in the solution technique. The graphite monochromatized Cu-Kα radiation (*λ* = 1.54184 Å) was used to measure the X-ray diffraction intensity data at 293 K using the X-ray scan method on a Rigaku XtaLAB Synergy-i Single Crystal X-ray Diffractometer with a CCD detector (HyPix-Bantam).

### 2.2 General procedure for the synthesis of amine derivatives

The synthesis of amine derivatives was carried out using the literature procedure.^43^ Nitroarene (1 mmol) and N_2_H_4_·H_2_O (4 equiv.) were dissolved in 3 mL of DMSO in an oven-dried 25 mL round bottom flask. MoO_3_ (22.75 mg, 25 mol%) was added to the reaction mixture and stirred at 120 °C for 15 min. After the completion of the reaction, as monitored by TLC (mobile phase, ethyl acetate : hexane = 50 : 50), the solution was cooled to room temperature, and the catalyst was removed by centrifugation. The reaction mixture was extracted with ethyl acetate (5 mL × 3). The organic layer was dried over anhydrous sodium sulfate, and the solvent was evaporated under reduced pressure. The final product was purified by silica gel column chromatography using ethyl acetate : hexane (25 : 75) as the eluent, and amine derivatives were obtained with a yield of 85–90%.

### 2.3 General procedure for the synthesis of 5-substituted-1*H*-tetrazole derivatives

A methodology from the literature was used to synthesize 5-substituted-1*H*-tetrazole derivatives.^44^ Benzonitrile (1 mmol) and sodium azide (1.5 mmol) were dissolved in 3 mL DMSO in a 25 mL round bottom flask. MoO_3_ (20.92 mg, 15 mol%) was added to the reaction mixture and stirred at 140 °C for 1.5 h. After the completion of the reaction (as monitored by TLC, mobile phase = ethyl acetate : hexane = 50 : 50), the solution was cooled to room temperature. The catalyst was removed by centrifugation. Note that 5 mL of ice water was added, followed by the dropwise addition of 3 N HCl until the reaction mixture became strongly acidic (pH 2). The reaction mixture was extracted with ethyl acetate (6 mL × 3). The organic layer was dehydrated with anhydrous sodium sulfate and evaporated *in vacuo*. The solid mass of 5-phenyl-1*H*-tetrazole derivatives was obtained with a yield of 85–91%.

## 3 Results and discussion

### 3.1 Optimization for the transfer hydrogenation of nitrobenzene

The catalytic efficiency of the catalyst (MoO_3_ and MoO_2_) was assessed in the transfer hydrogenation (TH) of nitroarenes employing N_2_H_4_·H_2_O. At first, the reaction was done using nitrobenzene as a benchmark substrate in the absence of the catalyst and N_2_H_4_·H_2_O (6 equiv.) in methanol at 50 °C for 24 h. The reaction did not proceed (entries 1 and 2, Table 1), as monitored by TLC. Under the next optimized reaction conditions, the catalyst (MoO_3_ and MoO_2_) (5 mol%) and N_2_H_4_·H_2_O (6 equiv.) were added in methanol at 50 °C for 24 h and the yield was 11% and 10%, respectively (entries 3 and 4, Table 1). On increasing the amount of the catalyst (MoO_3_, 10 mol%) and using N_2_H_4_·H_2_O (6 equiv.) in ethanol at 60 °C, a yield of 20% was obtained in 24 h (entry 5, Table 1). Under another optimized reaction condition, the amount of hydrazine hydrate was (4 equiv.) and DMSO was used as a solvent at 120 °C. The obtained yield was 45% in 4 h (entry 6, Table 1). Further, MoO_2_ (10 mol%) was used as a catalyst with hydrazine hydrate (4 equiv.) and DMSO solvent at 120 °C to get a yield of 35% in 6 h (entry 7, Table 1). On enhancing the amount of catalyst (MoO_3_, 15 mol%) and hydrazine hydrate used (6 equiv.) in DMF at 120 °C, 50% yield was obtained in 8 h (entry 8, Table 1). On further increasing the catalyst amount of both MoO_3_ and MoO_2_ using 20 mol%, and hydrazine hydrate (4 and 6 equiv.) in DMSO at 120 °C, the yield obtained was 60% and 42% in 5 h, respectively (entries 9 and 10, Table 1). In DMF solvent, MoO_3_ was used at 25 mol% with 4 equiv. of hydrazine hydrate at 120 °C, and a yield of 80% was obtained in 2 h (entry 11, Table 1). With the same reaction, conditions were applied in DMSO to get a maximum yield of 90% in 15 min (entry 12, Table 1). Moreover, water was used as a solvent with an appropriate reaction condition at 80 °C but no changes were obtained (entry 13, Table 1). MoO_2_ was used as 25 mol% in DMSO solvent at 120 °C to get a yield of 48% in 4 h (entry 14, Table 1). Thus, the maximum yield (90%) was observed in DMSO (3 mL) at 120 °C for 15 min (entry 12, Table 1). The scope for the TH of various nitroarenes was investigated (Table 2). Utilizing the optimized conditions, several *para*- and *meta*-substituted nitroarenes presented a good-to-excellent yield of nitroamines (85–90%). In the formation of compounds 2d, 2e, and 2h, the selective reduction of the nitro group occurred due to its stronger negative inductive effect compared to that of the CHO/COOH group present in the same substrate moiety.

**Table 1:** Optimization for the transfer hydrogenation of nitrobenzene^a^

S. no.	Catalyst amount (mol%)	N_2_H_4_·H_2_O (equiv.)	Solvent	Time	Temp. (^o^C)	Yield^b^ (%)
1	Nil, MoO_3_	6	MeOH	24 h	50	—
2	Nil, MoO_2_	6	MeOH	24 h	50	—
3	5, MoO_3_	6	MeOH	24 h	50	11
4	5, MoO_2_	6	MeOH	24 h	50	10
5	10, MoO_3_	6	EtOH	24 h	60	20
6	10, MoO_3_	4	DMSO	4 h	120	45
7	10, MoO_2_	4	DMSO	6 h	120	35
8	15, MoO_3_	6	DMF	8 h	120	50
9	20, MoO_3_	4	DMSO	5 h	120	60
10	20, MoO_2_	6	DMSO	5 h	120	42
11	25, MoO_3_	4	DMF	2 h	120	80
12	25, MoO_3_	4	DMSO	15 min	120	90
13	25, MoO_3_	4	H_2_O	24 h	80	—
14	25, MoO_2_	4	DMSO	4 h	120	48

a Reaction condition: nitrobenzene (1 mmol), N_2_H_4_·H_2_O (4 equiv.), solvent (3 mL), and catalyst (mol%).

b Isolated yields.

**Table 2:** Substrate scope for the MoO_3_-catalyzed synthesis of amine derivatives^a^^,^^c^

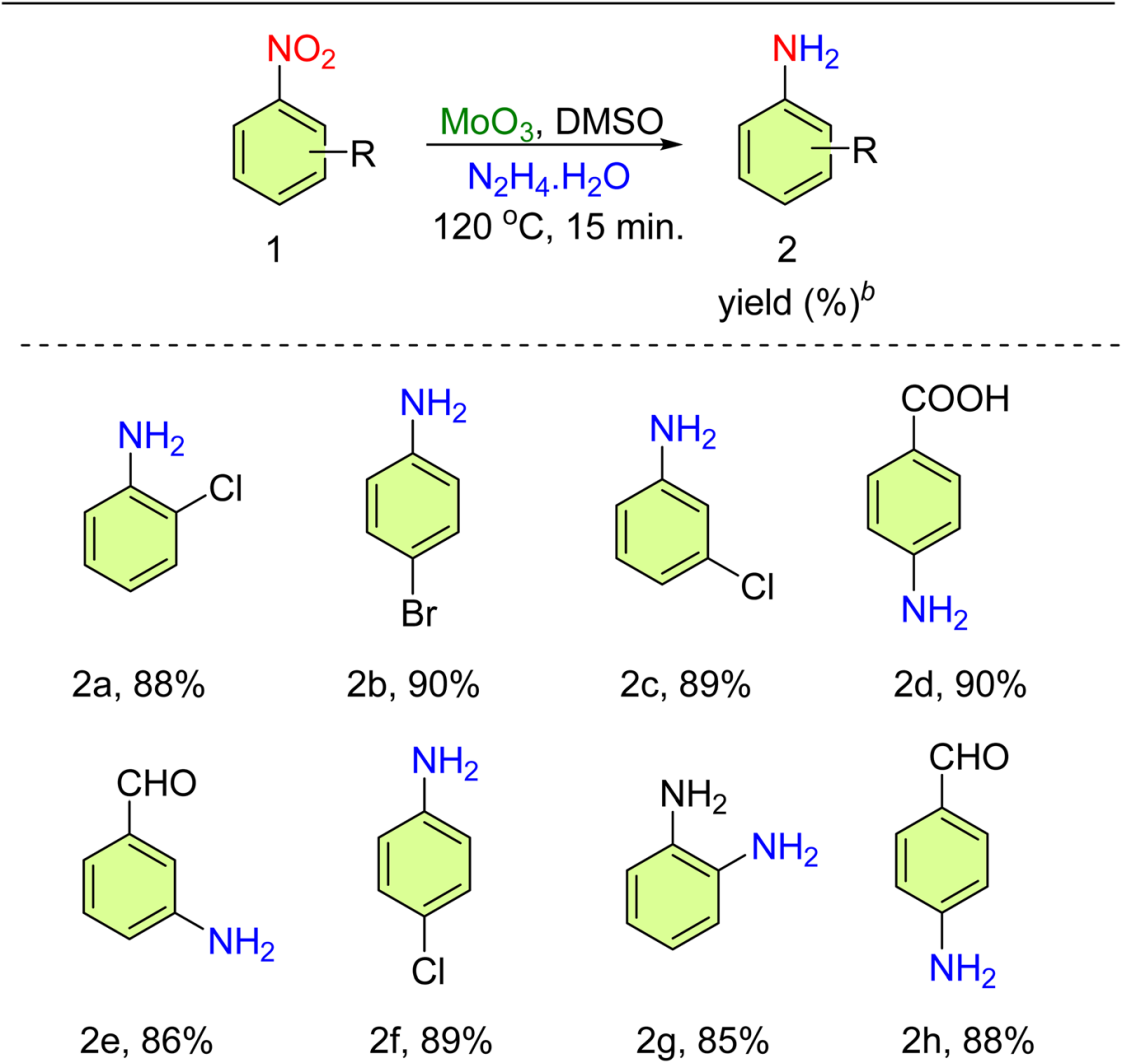

a Reaction condition: nitroarene (1 mmol), MoO_3_ (25 mol%), hydrazine hydrate (4 mmol), DMSO (3 mL), 120 °C, 15 min.

b Isolated yield.

c Products were characterized using ^1^H and ^13^C spectroscopy and HRMS.

**Fig. 3:**
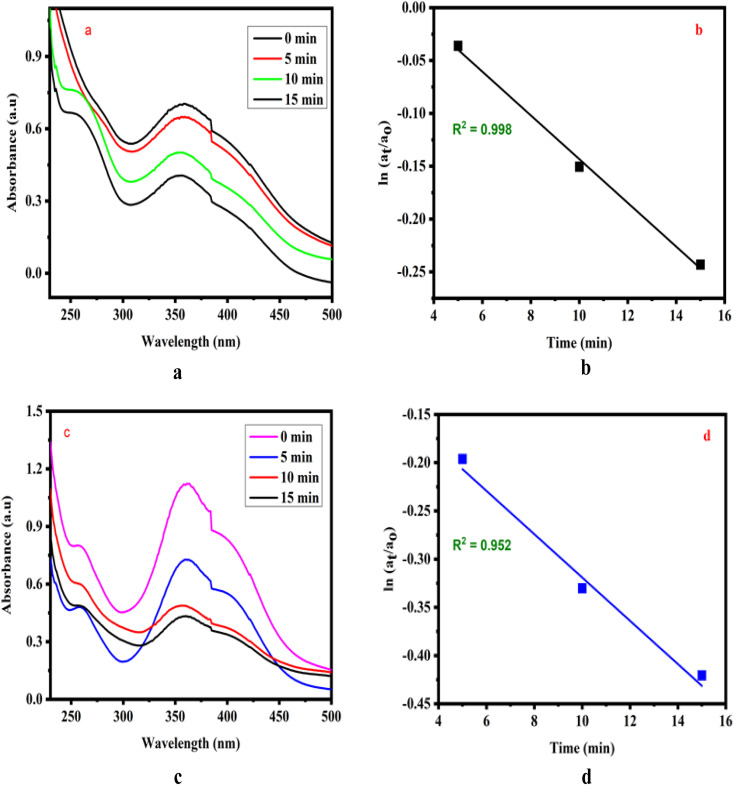
(a) UV-vis spectra for the hydrogenation of 2,4-dinitrophenol and (b) relationship between ln(*a*_t_/*a*_0_) and reaction time (min). (c) UV-vis spectra of the hydrogenation of picric acid and (d) relationship between ln(*a*_t_/*a*_0_) and reaction time (min).

Analogues of 2,4-dinitrophenol and picric acid exhibited pseudo-first-order kinetics, as illustrated in Fig. 3(a–d). Upon comparison, it was observed that the rate constant for 2,4-dinitrophenol was higher than that of 2,4,6-trinitrophenol. This variation in the rate constants is attributed to the structural differences between the compounds, with steric hindrance playing a significant role in the di- and tri-substituted derivatives.

**Fig. 4:**
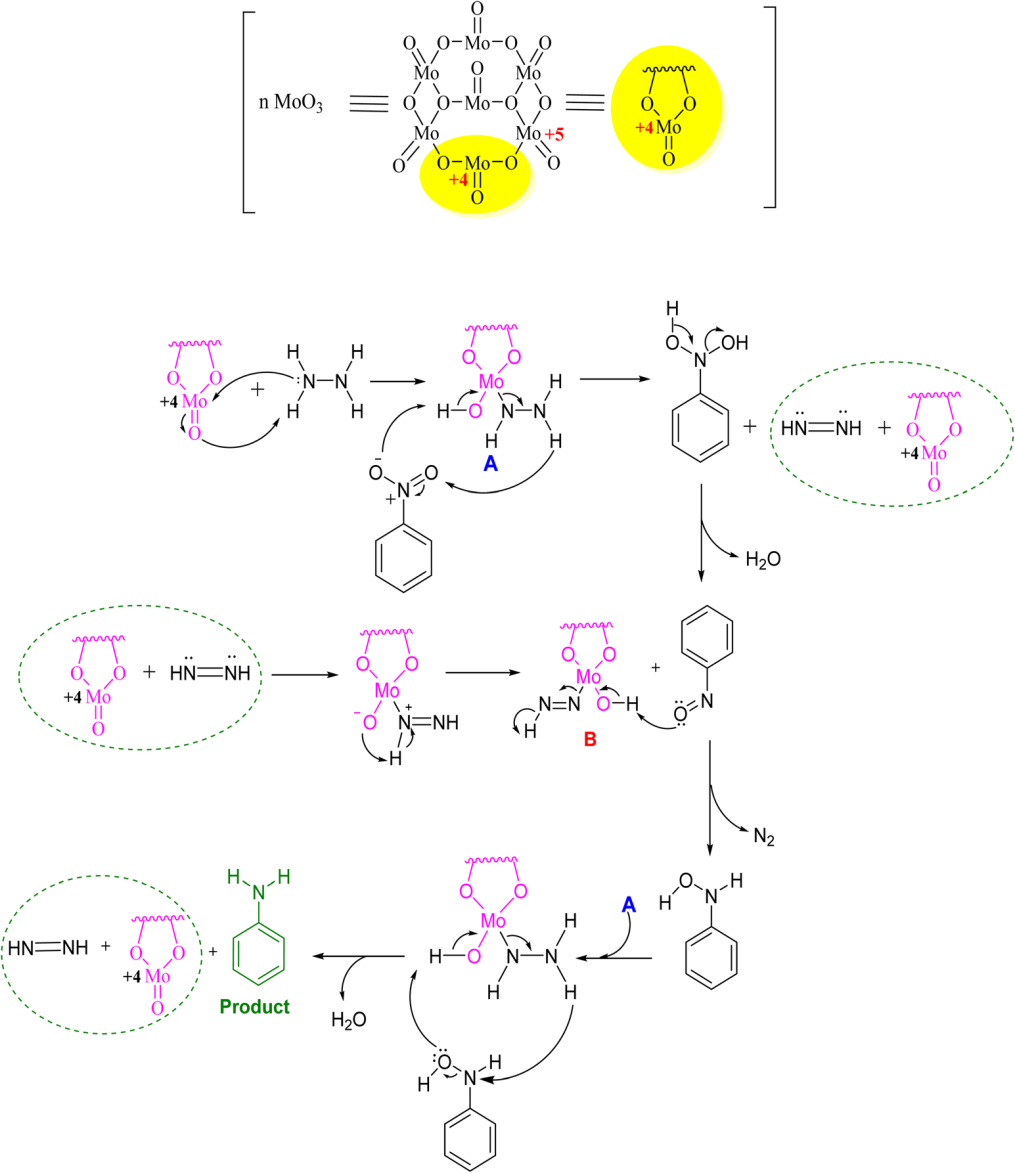
Plausible mechanism for the synthesis of amine derivatives using MoO_3_.

In Fig. 4, the plausible mechanism representing the role of MoO_3_ for synthesizing amine derivatives is shown using nitrobenzene as the representative substrate. The catalytic reduction of nitroarenes to anilines occurs by the reaction of MoO_3_ with hydrazine hydrate. It forms a complex *in situ* that reacts with nitroarene to produce the nitroso derivative and diimide intermediate of MoO_3_. Both the above reacted and formed the hydroxylamine species and nitrogen complex of MoO_3_ and then underwent reduction with hydrazine hydrate to form the final product, *i.e.*, amine derivative.

### 3.2 Optimization of the reaction conditions for the synthesis of 5-phenyl-1*H*-tetrazole

Our goal is to develop an improved catalytic system by evaluating molybdenum dioxide (MoO_2_) and molybdenum trioxide (MoO_3_) for their effectiveness in catalyzing the [3 + 2] cycloaddition between benzonitrile and sodium azide to synthesize 5-phenyl-1*H*-tetrazole in dimethylsulfoxide (DMSO). We optimized the catalyst amount, solvent, and temperature to achieve a high yield of 5-phenyl-1*H*-tetrazole in a shorter reaction time. The outcomes of these experiments are summarized in Table 3. In an initial attempt, the reaction was carried out without any catalyst using DMF and DMSO as solvents. However, no progress was observed after 24 h at 140 °C and 120 °C, respectively (entries 1 and 2, Table 3). Thin-layer chromatography (TLC) was used to monitor the progress of the reaction. The effectiveness of MoO_2_ and MoO_3_ (2.5 mol%) as catalysts was tested in various solvents (DMSO and DMF) at 140 °C. This resulted in a meagre yield (25–30%) of the desired product after 15 and 12 h, respectively (entries 3 and 4, Table 3). Further, the amount of the catalyst was increased (MoO_3_ and MoO_2_) and was taken at 5 mol% in DMSO solvent at 140 °C to get the product; the yield was (40–45%) after 10 and 7 h of the reaction, respectively (entries 5 and 6, Table 3). Again, the reaction was attempted with an increased amount of catalyst (MoO_3_, 10 mol%) in various solvents (ethanol, acetonitrile, water, DMF, and DMSO) at different temperatures (78 °C, 80 °C, 90 °C, and 140 °C, respectively) (entries 7–11, Table 3). Among these conditions, the highest yield of 5-phenyl-1*H*-tetrazole (65%) was obtained when DMSO was used as the solvent at 140 °C for 5 h (entry 11, Table 3). Further, in the optimization process, MoO_2_ (10 mol%) was used as a catalyst in DMSO solvent and a yield of 57% was obtained in 7 h at 140 °C (entry 12, Table 3). In the next step, a larger amount of the catalyst was added to the reaction (MoO_3_, 15 mol%) in DMF and DMSO solvent system at 140 °C, and the desired yield of the product obtained was 72% in 3 h and 91% in 1.5 h (entries 13 and 14, Table 3). Further, MoO_2_ (15 mol%) was used in DMF and DMSO solvents and yields of 42% and 45% in 5 h at 140 °C (entries 15–16, Table 3). Thus, the optimized reaction condition was determined to be MoO_3_ (15 mol%) as a catalyst in DMSO (3 mL) at 140 °C. Under this condition, the product 5-phenyl-1*H*-tetrazole was isolated with 91% yield after 1.5 h of reaction.

**Table 3:** Optimization of the reaction conditions for the synthesis of 5-phenyl-1*H*-tetrazole^a^

S. no.	Catalyst	Catalyst amount (mol%)	Solvent	Temp. (^o^C)	Time (h)	Yield^b^ (%)
1	MoO_3_	Nil	DMF	140	24	—
2	MoO_2_	Nil	DMSO	120	24	—
3	MoO_2_	2.5	DMSO	140	15	30
4	MoO_3_	2.5	DMF	140	12	25
5	MoO_2_	5	DMSO	120	10	40
6	MoO_3_	5	DMSO	120	7	45
7	MoO_3_	10	EtOH	78	24	—
8	MoO_3_	10	CH_3_CN	80	12	20
9	MoO_3_	10	H_2_O	90	12	—
10	MoO_3_	10	DMF	140	6	52
11	MoO_3_	10	DMSO	140	5	65
12	MoO_2_	10	DMSO	140	7	57
13	MoO_3_	15	DMF	140	3	72
14	MoO_3_	15	DMSO	140	1.5	91
15	MoO_2_	15	DMF	140	5	42
16	MoO_2_	15	DMSO	140	5	45

a Reaction condition: benzonitrile (1 mmol), NaN_3_ (1.5 mmol), solvent (3 mL), and catalyst (15 mol%) at 140 °C, 1.5 h.

b Isolated yield.

After establishing the optimal reaction conditions, we extended the substrate scope for synthesizing MoO_3_-catalyzed 5-substituted-1*H*-tetrazole derivatives to include aromatic and heteroaromatic nitriles. The results, summarized in Table 4, show good to excellent yields (85–91%) of 5-substituted-1*H*-tetrazoles. Aromatic nitriles with electron-withdrawing groups yielded higher amounts of the corresponding 5-substituted-1*H*-tetrazoles than those with electron-donating groups. Additionally, heteroaromatic nitriles produced the corresponding 5-substituted-1*H*-tetrazoles with excellent yields (entry 5n, Table 4).

**Table 4:** Substrate scope for the MoO_3_-catalyzed synthesis of 5-substituted-1*H*-tetrazoles^a^^,^^c^

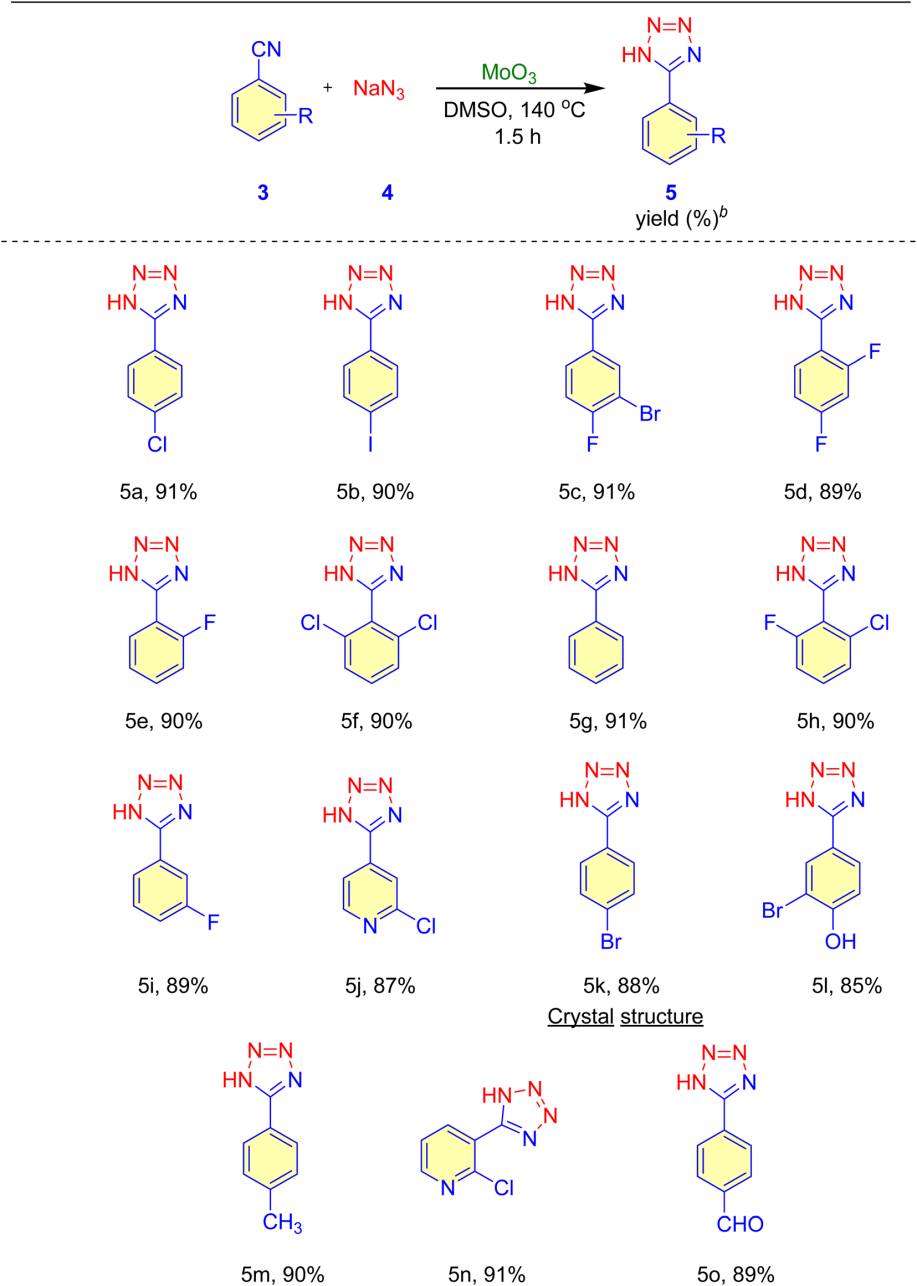

a Reaction condition: nitrile (1 mmol), NaN_3_ (1.5 mmol), DMSO (3 mL) and MoO_3_ (15 mol%) at 140 °C, 1.5 h.

b Isolated yields.

c Products were characterized using ^1^H and ^13^C NMR, IR spectroscopy and HRMS.

**Fig. 5:**
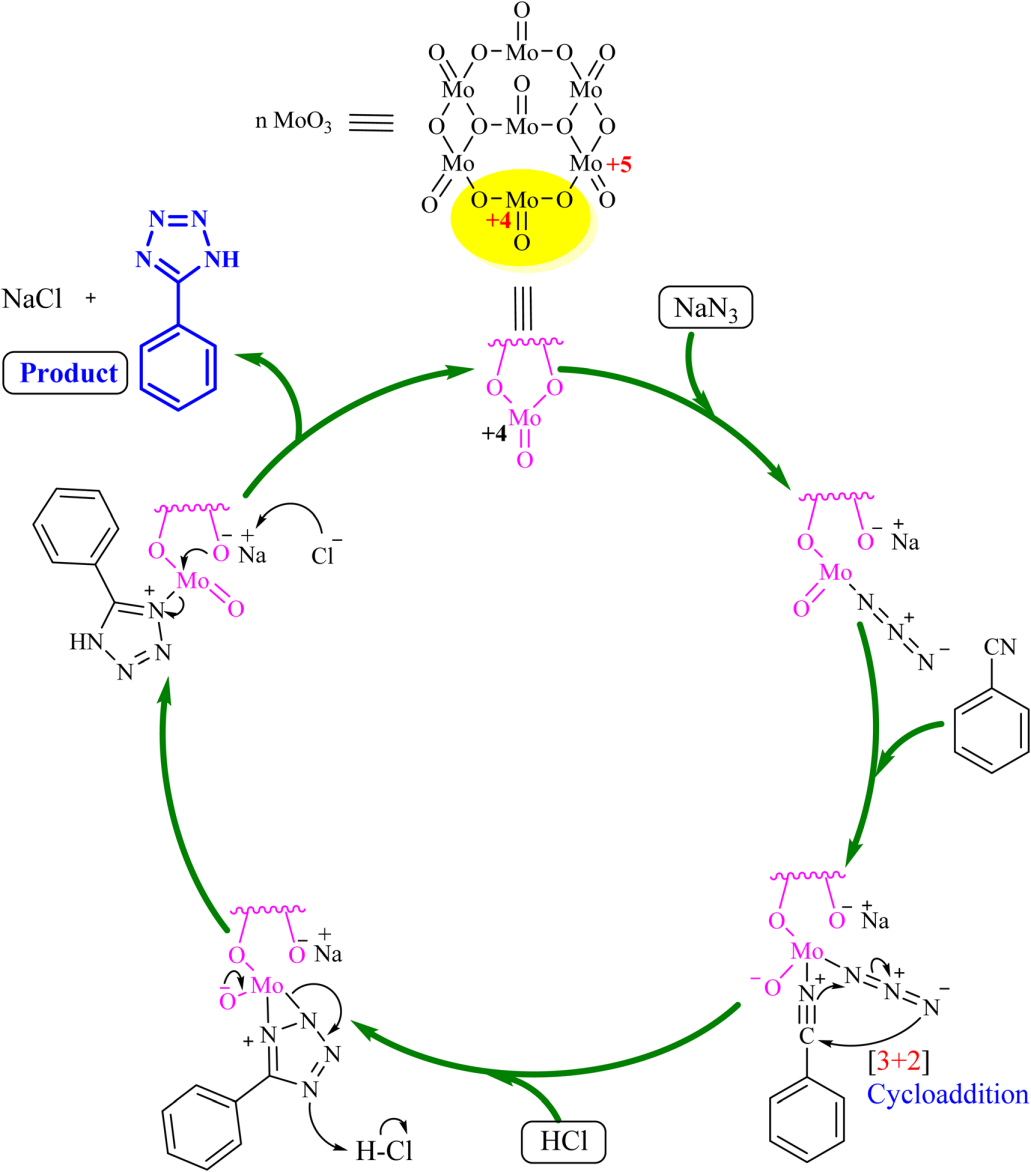
Plausible mechanism for the synthesis of 5-substituted-1*H*-tetrazoles using MoO_3_.

**Fig. 6:**
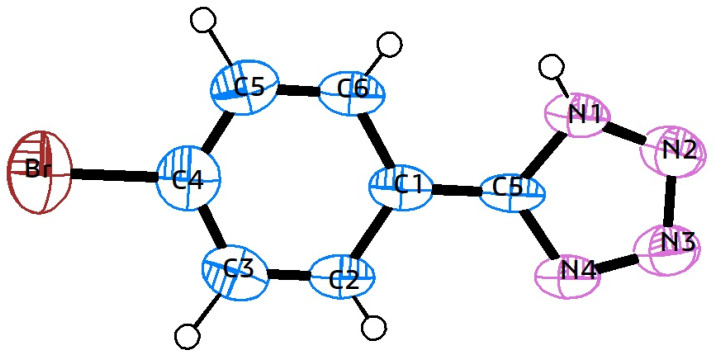
ORTEP diagram of compound 5k.

Fig. 5 illustrates a plausible mechanism for the role of MoO_3_ in synthesizing 5-substituted-1*H*-tetrazoles using benzonitrile as a representative substrate. Initially, the nitrogen atom of the azide compound coordinates with MoO_3_ to form an intermediate. This intermediate facilitates the [3 + 2] cycloaddition between the azide ion and the nitrile compound's (–C≡N) bond, resulting in complex formation. After the reaction, MoO_3_ is separated by centrifugation, and the solution's pH is adjusted to 2 using 3 N HCl to obtain 5-phenyl-1*H*-tetrazole as the final product. The synthesis approach for 5-substituted-1*H*-tetrazoles was further validated using single-crystal X-ray analysis of compound 5k. X-ray quality crystals were grown in methanol using the slow evaporation method. Fig. 6 shows the ORTEP diagram of compound 5k, with additional single-crystal X-ray crystallographic details provided in Table 5.

**Table 5:** Crystal data, data collection, and structure refinement details for compound 5k

CCDC	2288459
Empirical formula	C_7_H_5_BrN_4_
Formula weight	112.53
Temperature/K	135.15
Wavelength	1.54184 Å
Crystal system	Orthorhombic
Space group	*Pbcm*
Hall group	−*P*2*c*2*b*
Unit cell dimensions	*a* = 4.14920(10) Å, *α* = 90°
	*b* = 19.9564(5) Å, *β* = 90°
	*c* = 9.7232(3) Å, *γ* = 90°
Volume	805.11(4)
*Z*	8
Density	1.857
Absorption coefficient	6.521 mm^−1^
*F* (000)	440.0
Theta range for data collection	8.862 to 144.142
Index ranges	−3 ≤ *h* ≤ 5
	−24 ≤ *k* ≤ 24
	−10 ≤ *l* ≤ 12
Reflections collected	4708
Absorption correction	Multi-scan
Refinement method	Full-matrix least-squares on *F*^2^
Goodness-of-fit on *F*^2^	1.088
Final *R* indices [*I* > 2sigma(*I*)]	*R*_1_ = 0.0393, w*R*_2_ = 0.1207

### 3.3 Green chemistry parameters

**Fig. 7:**
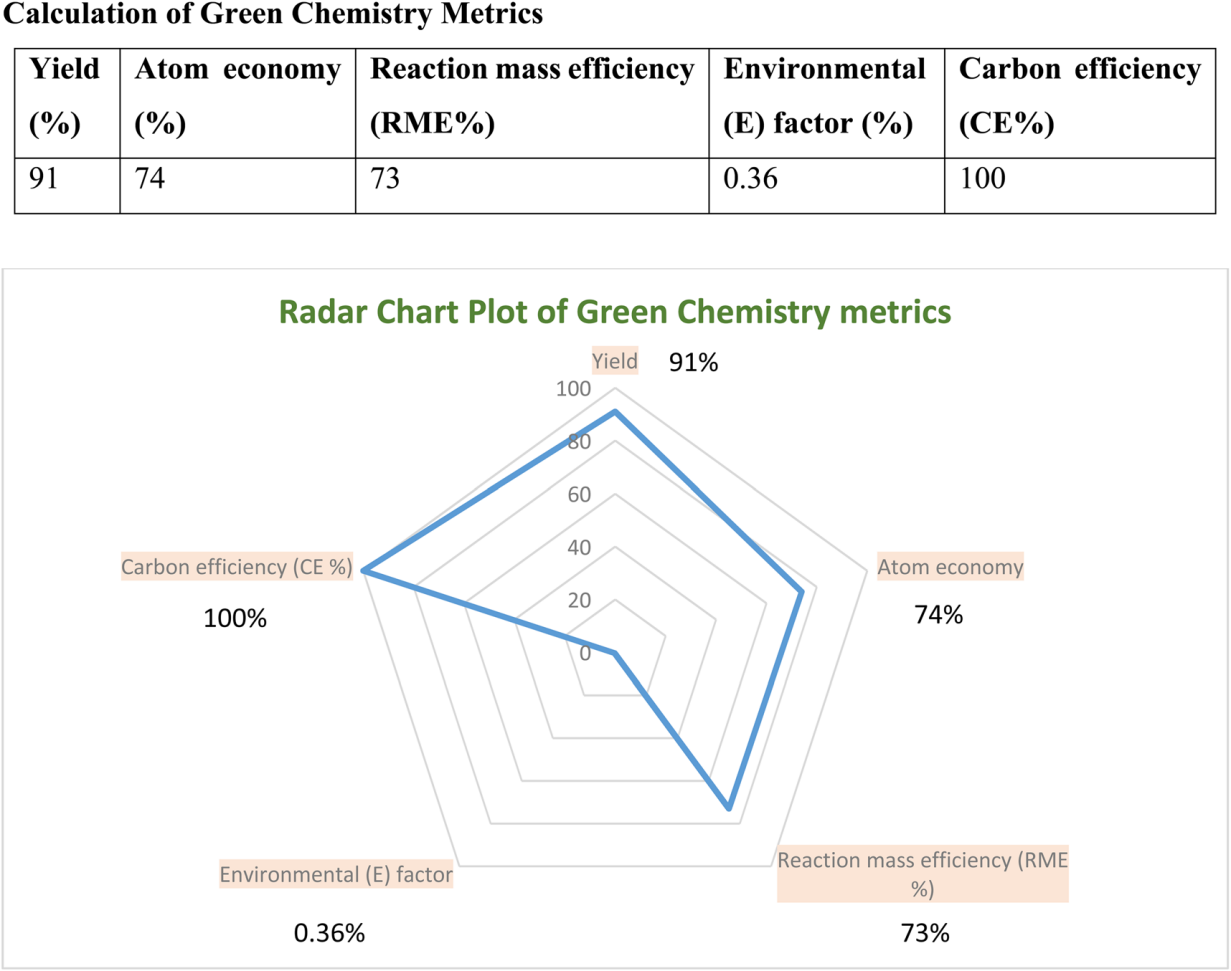
Illustrates the outcomes in a radar plot.

A thorough evaluation of green chemistry metrics was done to assess the synthesis of 5-phenyl-1*H*-tetrazole (5g). Table S1† presents the comprehensive calculations. Fig. 7 illustrates the outcomes in a radar plot, highlighting key variables pivotal in analyzing the cost-effectiveness of green organic synthesis, ideally under optimal conditions. The radar chart visually elucidates the synergistic correlation among parameters such as reaction mass efficiency, carbon efficiency, atom economy, and the *E*-factor, signifying the environment-friendly nature of this approach.

### 3.4 Reusability of the catalyst

**Fig. 8:**
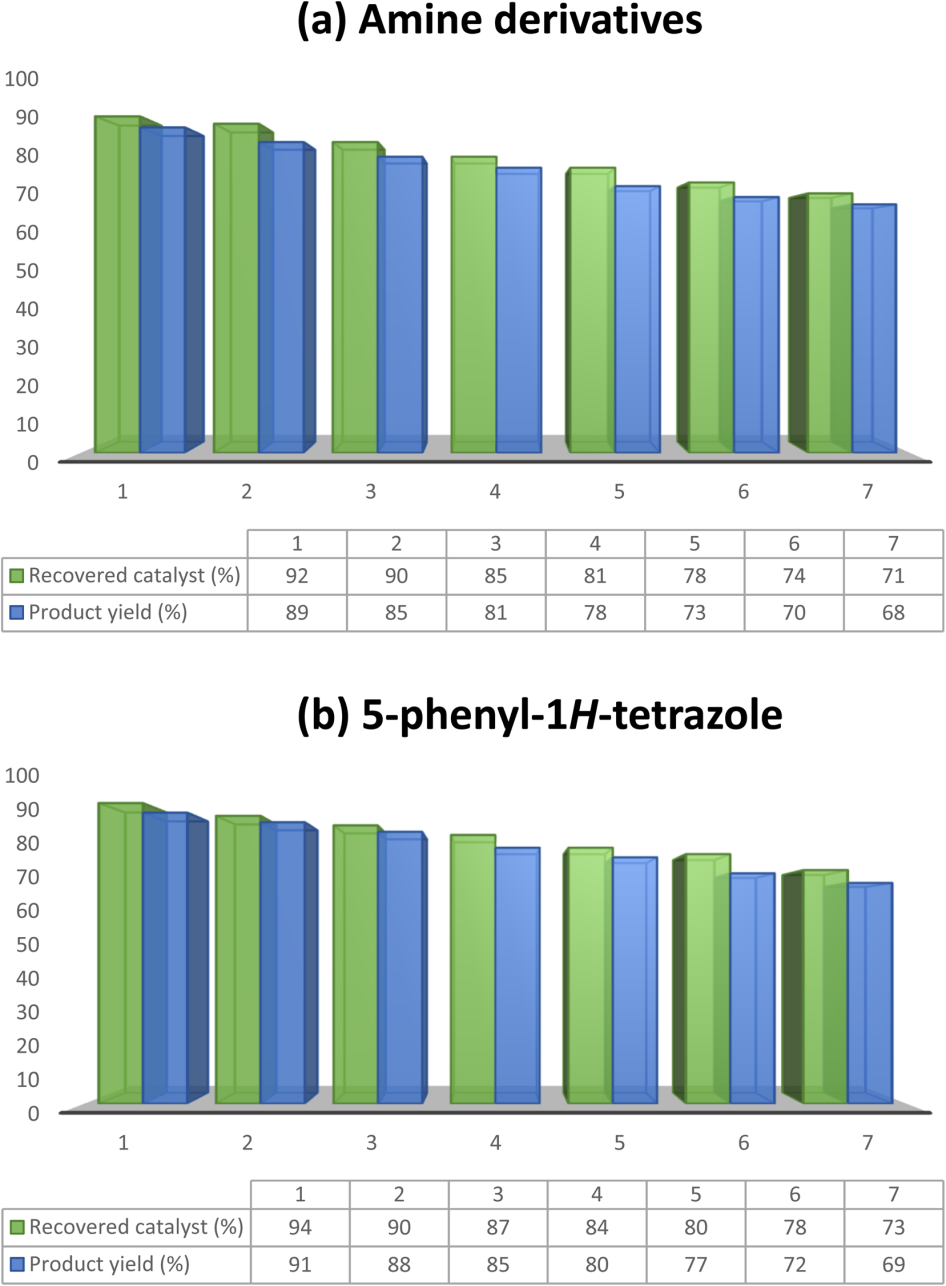
Plot representing the reusability of MoO_3_ for the synthesis of (a) 4-chloroaniline (2f) and (b) 5-phenyl-1*H*-tetrazole (5g) over seven catalytic cycles.

The capability of a heterogeneous catalyst that demonstrates convenient recoverability and efficient reusability is crucial for its practical application in industry. In this present investigation, the recoverability and reusability of MoO_3_ were scrutinized for the synthesis of amine and 5-phenyl-1*H*-tetrazole derivatives under the optimized reaction conditions. Following the completion of each cycle, MoO_3_ could be effortlessly retrieved *via* centrifugation, washed with ethyl alcohol, and subsequently dried at 60 °C in an oven for 1 h, ready for reuse in the subsequent cycle. Remarkably, MoO_3_ retained its catalytic activity over seven consecutive cycles without any significant decline. The yields (%) obtained for the seven catalytic cycles for both reactions are depicted in Fig. 8(a and b). Furthermore, the purity of the products was confirmed by analyzing the ^1^H NMR spectra of 4-chloroaniline (2f) and 5-phenyl-1*H*-tetrazole (5g) synthesized using the recovered catalyst after the seventh cycle.

### 3.5 Previously reported catalyst for the transformation of nitroarenes to arylamine

Based on existing literature, various methodologies have been employed for the transformation of nitroarenes to arylamines (Table 6).

**Table 6:** Previously reported catalysts for the transformation of nitroarenes to arylamine^a^

S. no.	Catalyst system	Catalyst amount	Reaction condition	Limitations	Yield (%)	Ref.
1	MoO_3_	25 mol%	DMSO (3 mL), 120 °C, 15 min	Less reaction time, medium catalyst loading, high yield	90	PW
2	Iron oxide hydroxide	0.2 g	Propan-2-ol, refluxed, 39 min	More reaction time, more catalyst	99	45
3	Fe-500-1 h	0.025 mmol	Alcohol (5 mL), 0.75–12 h, refluxed	More reaction time	96.7–100	46
4	Iron(iii) oxide hydroxide	0.15 g	MeOH (25 mL), 333 K, 1.5 h	More reaction time, more catalyst	96–100	47
5	[Co]	19 mol%	MeOH (0.3 mL), 50 °C, 6 h	More reaction time, low yield	50–60	43
6	MoO_2_	20 mg	EtOH (2 mL), 30–50 °C, 0.5–1 h	More reaction time	90–99	48

a PW = present work.

### 3.6 Previously reported catalyst for the synthesis of 5-substituted-1*H*-tetrazole

Based on existing literature, various synthetic methodologies have been employed for the synthesis of 5-substituted-1*H*-tetrazoles (Table 7).

**Table 7:** Previously reported catalyst for the synthesis of 5-substituted-1*H*-tetrazole^a^

S. no.	Catalyst system	Catalyst amount	Reaction condition	Limitations	Yield (%)	Ref.
1	MoO_3_	15 mol%	DMSO (3 mL), 140 °C, 1.5 h	Less reaction time, medium catalyst loading, high yield	91	PW
2	PbCl_2_	10 mg	DMF, 120 °C, 8–14 h	More reaction time, low yield	81	44
3	CuO-NrGO	10 mg	DMF (3 mL), 140 °C, 4 h	More reaction time	91	49
4	Cu(OAc)_2_	25 mol%	DMF, 120 °C, 12 h	More catalyst, more reaction time	98	50
5	CuFe_2_O_4_	40 mol%	DMF, 120 °C, 12 h	More catalyst, more reaction time, low yield	82	51
6	Fecl_3_-SiO_2_	50 mg	DMF, 120 °C, 12 h	More catalyst, more reaction time, low yield	79	52
7	Mesoporous-Zns	1.0 mmol	DMF, 120 °C, 36 h	More catalyst, more reaction time, low yield	86	53
8	Graphene	0.03 g	DMF (5 mL), 120 o°C, 36 h	More catalyst, more reaction time, low yield	63	54
9	SiO_2_–H_2_SO_4_	500 mg	DMF (10 mL), refluxed, 5 h	More catalyst, more reaction time, low yield	88	55
10	AgNO_3_	10 mmol	DMF (5 mL), 120 °C, 5 h	Low yield	83	56
11	GO/ZnO	0.03 g	DMF (5 mL), 120 °C, 30 h	More catalyst, more reaction time, low yield	78	57

a PW = present work.

## 4 Conclusion

In this study, molybdenum dioxide (MoO_2_) and molybdenum trioxide (MoO_3_) were utilized as catalysts for two reactions: the transfer hydrogenation of nitrobenzene to arylamine derivatives in DMSO at 120 °C for 15 min and the [3 + 2] cycloaddition between benzonitrile and sodium azide in dimethyl sulfoxide (DMSO) at 140 °C for 1.5 h. MoO_3_ emerged as the superior catalyst, delivering higher yields and shorter reaction times for the synthesis of the target compounds. The green chemistry metrics for these reactions revealed an *E*-factor of 0.36 (the ideal value is 0). The identity and purity of the synthesized compounds were verified using various analytical techniques, including ^1^H and ^13^C NMR, HRMS, FT-IR, and single-crystal XRD. The key advantages of this methodology include reduced reaction times, a wide range of applicable substrates, milder reaction conditions, and the use of cost-effective catalysts, making it highly appealing for industrial applications.

## Data availability

All relevant data are within the manuscript and its additional files. The data are available from the corresponding author on reasonable request.

## Author contributions

Anand Maurya: conceptualization; methodology; formal analysis; investigation; writing – original draft; and editing. Upendra Kumar Patel: formal analysis; single-crystal validation. Sanjeev Kumar: formal analysis. Alka Agarwal: conceptualization; resources; supervision; funding acquisition; and editing.

## Conflicts of interest

The authors declare that they have no known competing financial interests or personal relationships that could have appeared to influence the work reported in this paper.

## Supplementary Material

RA-014-D4RA05443A-s001

RA-014-D4RA05443A-s002
