# Supplementary material for: Molybdenum trioxide as a newer diversified economic catalyst for the transformation of nitroarenes to arylamine and 5-substituted-1H-tetrazole
Source: RSC Adv. 2024 Sep 18;14(40):29505–17. doi: 10.1039/d4ra05443a (PMC11409451; doi:10.1039/d4ra05443a)

Supplementary Information (SI) for RSC Advances.
This journal is © The Royal Society of Chemistry 2024

Supplementary Data

Molybdenum trioxide as a newer diversified economic catalyst for the transformation of nitroarenes to arylamine and 5-substituted-1*H*-tetrazole

Anand Maurya, Upendra Kumar Patel, Sanjeev Kumar, & Alka Agarwal*

Department of Medicinal Chemistry, Institute of Medical Sciences, Banaras Hindu University, Varanasi-221005, Uttar Pradesh, India

*Email: agarwal.dralka@gmail.com

Contents-

1. The calculation for green chemistry metrics for compound 5g**.**

2. ^{1}H & ^{13}C NMR data of amine derivatives.

3. ^{1}H & ^{13}C NMR data of 5-substituted-1*H*-tetrazoles derivatives.

4. Physical data of synthesized arylamine derivatives.

5. Physical data of synthesized 5-substituted-1*H*-tetrazole derivatives.

6. Physical data of 4-chloroaniline and 5-phenyl-1*H*-tetrazole after the seventh cycle.

1. **The calculation for green chemistry metrics for compound 5g.**

Table S1: The calculation for green chemistry metrics for compound **5g**.

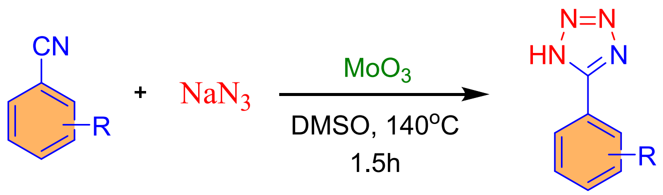

Details	Nitrile	Sodium azide	Product
**Molecular weight (g/mol)**	103.12	65.01	146.15
**Mmol**	1.0	1.5	0.91
**Amount in mg**	0.103	0.094	0.144

S. No.	Parameters	Formula	Characteristics	Ideal value	Calculated value for compound
**1**	Environmental (E) factor	[Total mass of raw materials - the total mass of product]/ mass of product	E-factor shows the total amount of waste generated in a chemical reaction.	0	[(0.103+0.094)-0.144]/0.144 =0.36
**2**	Process mass intensity (PMI)	Σ (mass of materials)/[Total mass of isolated product]	PMI takes into account reaction efficiency, stoichiometry, amount of solvent, and all	1	(0.103+0.094)/0.144 =1.36

			reagent used in the chemical reaction.		
**3**	Reaction mass efficiency (RME %)	[mass of product/Σ (mass of stoichiometric reactants)] × 100	RME accounts into atom economy, chemical yield, and stoichiometry.	100%	[0.144/(0.103+0.094)] X100 =73.09%
**4**	Atom economy	[MW of product] ÷ Σ(MW of stoichiometric reactants) × 100	Atom economy signifies the percentage of atoms wasted in a chemical reaction. The higher the value of AE, the greener is the reaction. The maximum value of atom economy is 100% which indicates that all the atoms present in reactants lie in the product.	100%	[(146.15)/(103.12+94.25)]X100 =74%
**5**	Carbon efficiency	[Amount of carbon in	CE signifies the percentage of	100%	[7/(7)]X100 =100%

(CE %)	product/Total carbon present in reactants]×100	carbons in the reactants that are left in the product.	

2. NMR (^1H & ^{13}C) data of amine derivatives

2-chloroaniline (2a)

Colorless liquid. Yield: 88 % (71 mg). ^{1}H NMR (600 MHz, $CDCl_3$) δ 7.37 (d, *J* = 7.1 Hz, 1H, Ar-H), 7.06 (t, *J* = 7.1 Hz, 1H, Ar-H), 6.70 (d, *J* = 6.8 Hz, 1H, Ar-H), 6.59 – 6.57 (m, 1H, Ar-H), 4.02 (s, 2H, $-NH_2$); ^{13}C NMR (151 MHz, $CDCl_3$) δ 142.97, 129.44, 127.68, 119.31, 119.06, 115.95.

4-bromoaniline (2b)

Light yellowish solid. Yield: 90 % (77 mg); mp 66-68 ^{o}C. ^{1}H NMR (600 MHz, $CDCl_3$) δ 7.22 (d, *J* = 8.5 Hz, 2H, Ar-H), 6.54 (d, *J* = 8.6 Hz, 2H, Ar-H), 3.65 (s, 2H, $-NH_2$); ^{13}C NMR (151 MHz, $CDCl_3$) δ 145.42, 131.97, 116.70, 110.12.

3-chloroaniline (2c)

Yellowish liquid. Yield: 89 % (72 mg). ^{1}H NMR (600 MHz, $CDCl_3$) δ 7.00 (t, *J* = 8.0 Hz, 1H, Ar-H), 6.68 (dd, *J* = 8.0, 1.7 Hz, 1H, Ar-H), 6.57 (s, 1H, Ar-H), 6.45 (dd, *J* = 8.1, 2.1 Hz, 1H, Ar-H), 3.65 (s, 2H, $-NH_2$); ^{13}C NMR (151 MHz, $CDCl_3$) δ 147.94, 134.81, 130.52, 118.40, 114.99, 113.42.

4-aminobenzoic acid (2d)

Yellow solid. Yield: 90 % (74 mg); mp 182-184 ^{o}C.^{1}H NMR (600 MHz, $CDCl_3$) δ 7.91 (d, *J* = 8.6 Hz, 2H, Ar-H), 6.66 (d, *J* = 8.6 Hz, 2H, Ar-H), 4.12 (s, 2H, $-NH_2$); ^{13}C NMR (151 MHz, $CDCl_3$) δ 168.77, 153.06, 151.70, 151.65, 131.59, 118.92, 113.36.

3-amino benzaldehyde (2e)

Light yellow solid. Yield: 86 % (69 mg); mp 30-32 ^{o}C.^{1}H NMR (600 MHz, DMSO-d_6) δ 8.49 (s, 1H, -CHO), 7.16 – 7.08 (m, 2H, Ar-H), 6.98 (d, *J* = 7.3 Hz, 1H, Ar-H), 6.72 (d, *J* = 7.5 Hz, 1H, Ar-H), 5.28 (s, 2H, $-NH_2$); ^{13}C NMR (151 MHz, DMSO-d_6) δ 162.24, 149.48, 134.84, 129.78, 117.61, 117.44, 112.80; HRMS: m/z calcd. for C_7H_7NO: 121.05, $[M+H]^+$ found: 122.06.

4-chloroaniline (2f)

Yellow solid. Yield: 89 % (72 mg); mp 75-77 ^{o}C.^{1}H NMR (600 MHz, $CDCl_3$) δ 7.07 (d, *J* = 8.6 Hz, 2H, Ar-H), 6.56 (d, *J* = 8.6 Hz, 2H, Ar-H), 3.63 (s, 2H, $-NH_2$); ^{13}C NMR (151 MHz, $CDCl_3$) δ 145.08, 129.14, 123.06, 116.29; HRMS: m/z calcd. for C_6H_6ClN: 127.02, $[M+H]^+$ found: 128.03.

benzene-1,2-diamine (2g)

Light brown solid. Yield: 85 % (67 mg); mp 103-105 °C.^{1}H NMR (600 MHz, $CDCl_3$) δ 6.78 – 6.60 (m, 4H, Ar-H), 3.36 (s, 4H, $-NH_2$); ^{13}C NMR (151 MHz, $CDCl_3$) δ 134.79, 120.30, 116.79.

4-aminobenzaldehyde (2h)

Yellow solid. Yield: 88 % (70 mg); mp 73-75 °C. ^{1}H NMR (600 MHz, DMSO-d_6) δ 8.46 (s, 1H, -CHO), 7.54 (d, J = 8.5 Hz, 2H, Ar-H), 6.66 (d, J = 8.5 Hz, 2H, Ar-H), 5.77 (s, 2H, $-NH_2$); ^{13}C NMR (151 MHz, DMSO-d_6) δ 160.25, 152.10, 130.20, 121.99, 113.97.

3. NMR (^{1}H & ^{13}C) data of 5-substituted-1*H*-tetrazoles derivatives

5-(4-chlorophenyl)-1H-tetrazole (5a)

Light green solid. Yield: 91 %, (119 mg); mp 236-238 °C. ^{1}H NMR (δ ppm) (600 MHz, DMSO-d_6) δ 8.06 (d, J = 7.7 Hz, 2H, Ar-H), 7.69 (d, J = 7.7 Hz, 2H, Ar-H); ^{13}C NMR (δ ppm) (151 MHz, DMSO-d_6) 155.38, 136.37, 130.21, 129.18, 123.70; FTIR (cm^{-1}): 3400, 2200, 1600, 1100, 730; HRMS: m/z calcd. for $C_7H_5ClN_4$: 180.02, $[M+H]^+$ found: 181.02.

5-(4-iodophenyl)-1H-tetrazole (5b)

Light brown solid. Yield: 90 % (106 mg); mp 244-246 °C. ^{1}H NMR (δ ppm) (600 MHz, DMSO-d_6) δ 8.00 (d, J = 7.7 Hz, 2H, Ar-H), 7.82 (d, J = 7.7 Hz, 2H, Ar-H); ^{13}C NMR (δ ppm) (151 MHz, DMSO-d_6) δ 155.67138.77, 134.09, 129.17, 98.92; FTIR (cm^{-1}): 3400, 2500, 1800, 1120, 621.

5-(3-bromo-4-fluorophenyl)-1H-tetrazole (5c)

Off-white solid. Yield: 91 % (110 mg); mp 158-160 °C. ^{1}H NMR (δ ppm) (600 MHz, DMSO-d_6) δ 8.22 (s, 1H, Ar-H), 7.91 (d, J = 8.3 Hz, 1H, Ar-H), 7.59 (d, J = 8.3 Hz, 1H, Ar-H); ^{13}C NMR (δ ppm) (151 MHz, DMSO-d_6) δ 144.04137.36, 133.44, 121.74, 117.77, 113.40, 108.75; FTIR (cm^{-1}): 3400, 3050, 2150, 1600, 1110.

5-(2,4-difluorophenyl)-1H-tetrazole (5d)

Brown solid. Yield: 89 % (116 mg); mp 149-151 °C. ^{1}H NMR (δ ppm) (600 MHz, DMSO-d_6) δ 7.88 (d, *J* = 8.4 Hz, 1H, Ar-H), 7.28 (s, 1H, Ar-H), 7.12 (d, *J* = 8.4 Hz, 1H, Ar-H); ^{13}C NMR (δ ppm) (151 MHz, DMSO-d_6) δ 166.41164.72, 145.17, 135.73, 116.83, 111.13, 108.50; FTIR (cm^{-1}): 3400, 3020, 2220, 1600, 820.

5-(2-fluorophenyl)-1H-tetrazole (5e)

Light brown solid. Yield: 90 % (122 mg); mp 129-131 °C. ^{1}H NMR (δ ppm) (600 MHz, DMSO-d_6) δ 7.81 (d, *J* = 7.6 Hz, 1H, Ar-H), 7.75 (t, *J* = 7.8 Hz, 1H, Ar-H), 7.53 (d, *J* = 8.2 Hz, 1H, Ar-H), 7.33 (t, *J* = 7.5 Hz, 1H, Ar-H); ^{13}C NMR (δ ppm) (151 MHz, DMSO-d_6) δ 143.38, 135.23, 134.35, 125.83, 120.29, 116.23, 103.00.

5-(2,6-dichlorophenyl)-1H-tetrazole (5f)

Cream solid. Yield: 90 % (113 mg); mp 118-120 °C. ^{1}H NMR (δ ppm) (600 MHz, DMSO-d_6) δ 7.79 – 7.71 (m, 1H, Ar-H), 7.55 (d, *J* = 8.3 Hz, 1H, Ar-H), 7.51 (d, *J* = 8.1 Hz, 1H, Ar-H); ^{13}C NMR (δ ppm) (151 MHz, DMSO-d_6) δ 145.99 ,137.08, 135.85, 129.34, 126.08, 119.27, 116.01; FTIR (cm^{-1}): 3400, 3040, 2200, 1600, 1350. HRMS: m/z calcd. for $C_7H_4Cl_2N_4$: 213.98, $[M+H]^+$ found: 214.91.

5-phenyl-1H-tetrazole (5g)

Snow white solid. Yield: 91 % (127 mg); mp 204-206 °C. ^{1}H NMR (δ ppm) (600 MHz, DMSO-d_6) δ 8.05 (d, *J* = 7.0 Hz, 2H, Ar-H), 7.60 (t, *J* = 9.4 Hz, 3H, Ar-H); ^{13}C NMR (δ ppm) (151 MHz, DMSO-d_6) δ 155.77131.72, 129.89, 127.43, 124.62; FTIR (cm^{-1}): 3400, 2720, 2530, 1600, 1050, 740.

5-(2-chloro-6-fluorophenyl)-1H-tetrazole (5h)

Light brown solid. Yield: 90 % (114 mg); mp 148-150 °C. ^{1}H NMR (δ ppm) (600 MHz, DMSO-d_6) δ 7.75 (t, *J* = 8.2 Hz, 1H, Ar-H), 7.55 (d, *J* = 8.3 Hz, 1H, Ar-H), 7.50 (d, *J* = 8.1 Hz, 1H, Ar-H); ^{13}C NMR (δ ppm) (151 MHz, DMSO-d_6) δ 145.98137.08, 135.84, 126.07, 119.26, 116.00, 113.60; FTIR (cm^{-1}): 3400, 3020, 1800, 1600, 710.

5-(3-fluorophenyl)-1H-tetrazole (5i)

Cream solid. Yield: 89 % (120 mg); mp 128-130 °C. ^{1}H NMR (δ ppm) (600 MHz, DMSO-d_6) δ 7.90 (d, J = 7.6 Hz, 1H, Ar-H), 7.83 (d, J = 9.6 Hz, 1H, Ar-H), 7.69 – 7.42 (m, 2H, Ar-H); ^{13}C NMR (δ ppm) (151 MHz, DMSO-d_6) δ 163.60161.98, 155.64, 132.24, 132.19, 127.31, 127.26; FTIR (cm^{-1}): 3400, 3020, 2110, 1600, 1120, 751.

2-chloro-4-(1H-tetrazol-5-yl)pyridine (5j)

Off-white solid. Yield: 87 % (114 mg); mp 183-185 °C. ^{1}H NMR (δ ppm) (600 MHz, DMSO-d_6) δ 8.66 (d, J = 4.6 Hz, 1H, Ar-H), 8.06 (s, 1H, Ar-H), 8.01 (d, J = 4.9 Hz, 1H, Ar-H); ^{13}C NMR (δ ppm) (151 MHz, DMSO-d_6) δ 155.25151.87, 151.78, 136.12, 121.71, 120.76; FTIR (cm^{-1}): 3420, 3030, 1850, 1610.

5-(4-bromophenyl)-1H-tetrazole (5k)

Grey solid. Yield: 88 % (108 mg); mp 237-239 °C. ^{1}H NMR (δ ppm) (600 MHz, DMSO-d_6) δ 7.99 (d, J = 7.8 Hz, 2H, Ar-H), 7.84 (d, J = 7.7 Hz, 2H, Ar-H); ^{13}C NMR (δ ppm) (151 MHz, DMSO-d_6) δ 134.60, 133.12, 132.97, 129.36, 125.18, 124.04; FTIR (cm^{-1}): 3400, 3120, 2200, 1600, 1020.

2-bromo-4-(1H-tetrazol-5-yl)phenol (5l)

Yellow solid. Yield: 85 % (103 mg); mp 200-202 °C. ^{1}H NMR (δ ppm) (600 MHz, DMSO-d_6) δ 11.08 (s, 1H, Ar-H), 8.16 (s, 1H, Ar-H), 7.88 (d, J = 8.4 Hz, 1H, Ar-H), 7.14 (d, J = 8.4 Hz, 1H, Ar-H); ^{13}C NMR (δ ppm) (151 MHz, DMSO-d_6) δ 159.05, 157.18, 133.85, 131.98, 128.32, 117.44, 110.42; FTIR (cm^{-1}): 3450, 3020, 2615, 1700, 1600, 1100. HRMS: m/z calcd. for $C_7H_5BrN_4O$: 239.96, $[M+H]^+$ found: 240.97.

5-(p-tolyl)-1H-tetrazole (5m)

White solid. Yield: 90 % (122 mg); mp 240-242 °C. ^{1}H NMR (δ ppm) (600 MHz, DMSO-d_6) δ 7.93 (d, J = 7.6 Hz, 2H, Ar-H), 7.42 (d, J = 7.7 Hz, 2H, Ar-H), 2.39 (s, 3H, -CH_3); ^{13}C NMR (δ ppm) (151 MHz, DMSO-d_6) δ 141.71, 132.56, 130.42, 127.36, 121.73, 21.50; FTIR (cm^{-1}): 3400, 3020, 1900, 1600, 1450, 760.

2-chloro-3-(1H-tetrazol-5-yl)pyridine (5n)

Light green solid. Yield: 91 % (119 mg); mp 210-212 °C. ^{1}H NMR (δ ppm) (600 MHz, DMSO-d_6) δ 9.65 (d, *J* = 6.9 Hz, 1H, Ar-H), 8.61 (d, *J* = 7.1 Hz, 1H, Ar-H), 7.62 (t, *J* = 7.0 Hz, 1H, Ar-H); ^{13}C NMR (δ ppm) (151 MHz, DMSO-d_6) δ 147.79, 141.70, 131.93, 117.41, 114.21, 100.02; FTIR (cm^{-1}): 3400, 3050, 2650, 1600, 1200, 740.

4-(1H-tetrazol-5-yl)benzaldehyde (5o)

Green solid. Yield: 89 % (118 mg); mp 215-217 °C. ^{1}H NMR (δ ppm) (600 MHz, DMSO-d_6) δ 10.09 (s, 1H, -CHO), 8.25 (d, *J* = 7.1 Hz, 2H, Ar-H), 8.12 (d, *J* = 7.2 Hz, 2H, Ar-H); ^{13}C NMR (δ ppm) (151 MHz, DMSO-d_6) δ 193.13,155.96, 138.06, 130.82, 130.72, 128.22, 128.06; FTIR (cm^{-1}): 3400, 3020, 2650, 1600, 1190. HRMS: m/z calcd. for $C_8H_6N_4O$: 174.05, $[M+H]^+$ found: 175.06.

4. Physical data of synthesized amine derivatives.

1H NMR of 2-chloroaniline (2a)

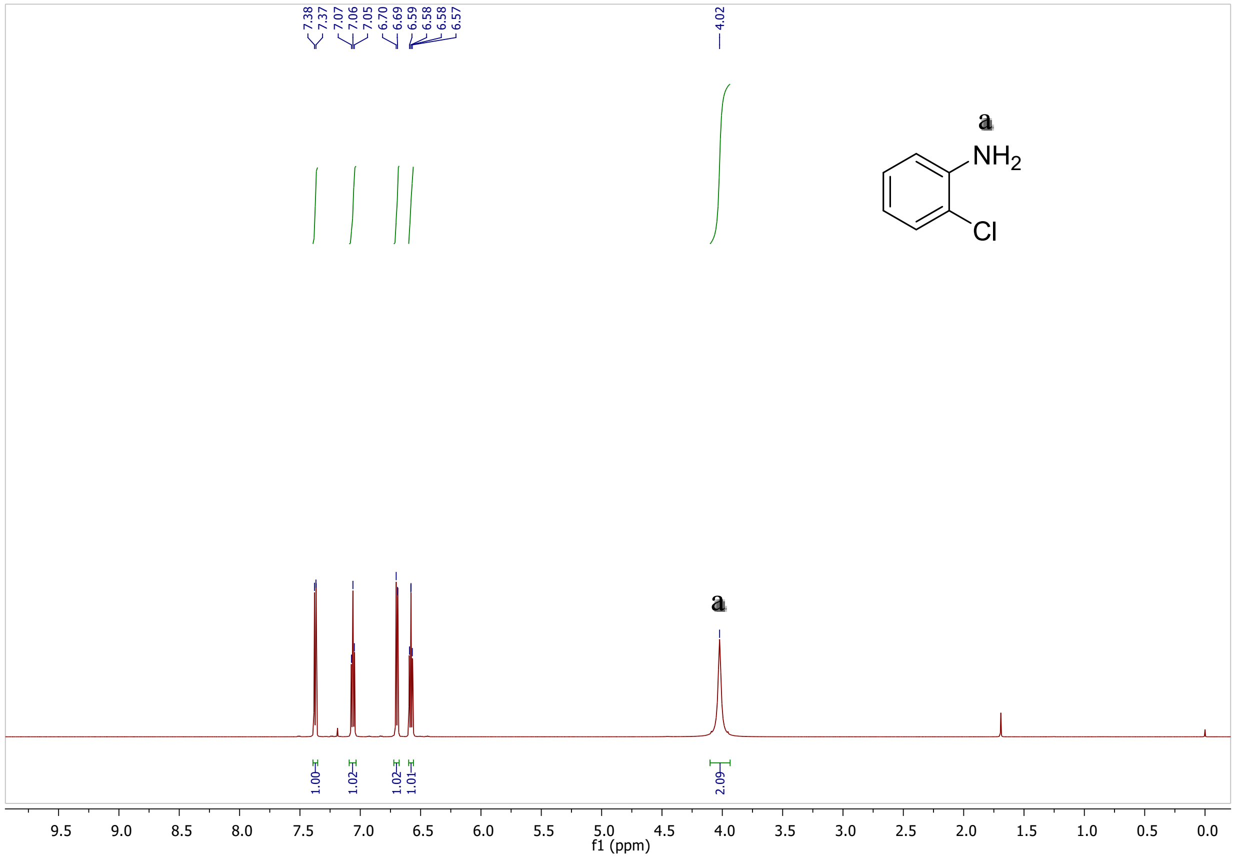

^{13}C NMR of 2-chloroaniline (2a)

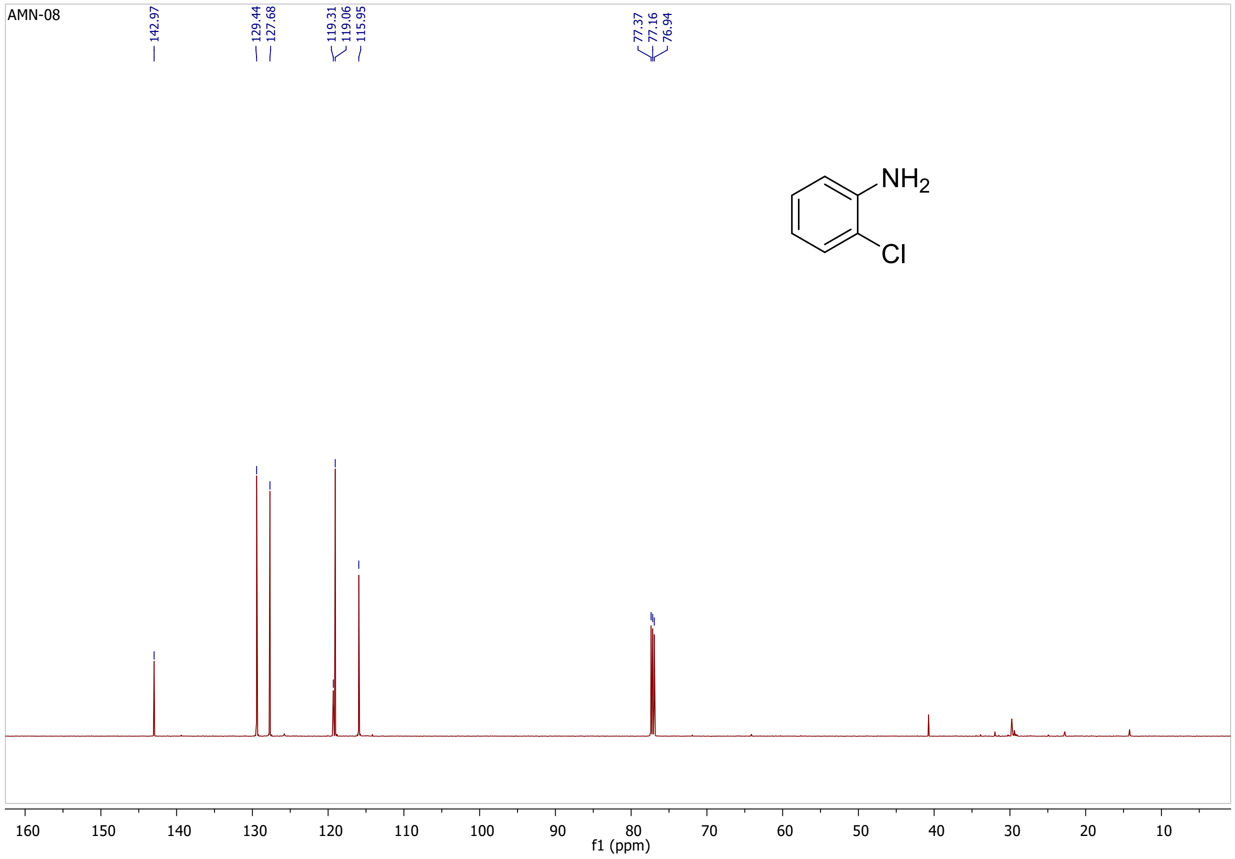

^{1}H NMR of 4-bromoaniline (2b)

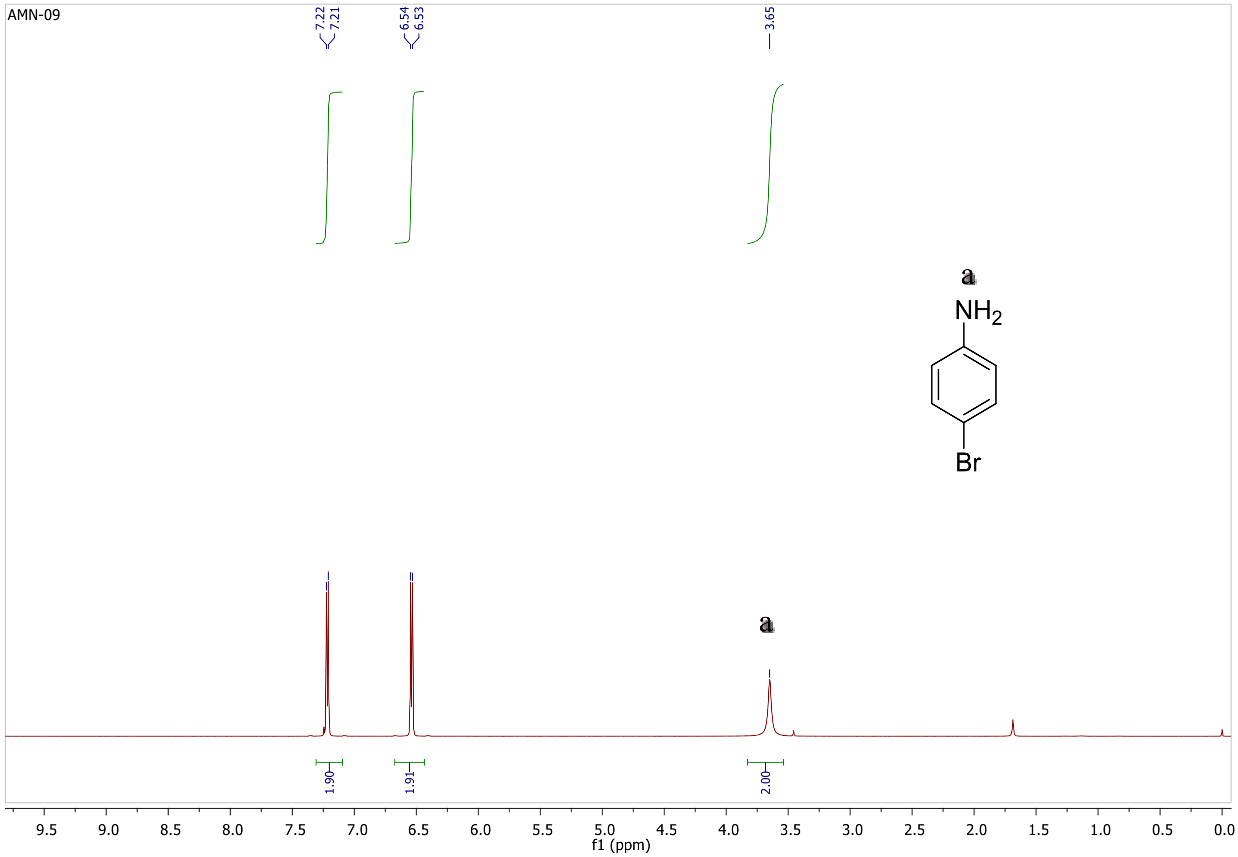

^{13}C NMR of 4-bromoaniline (2b)

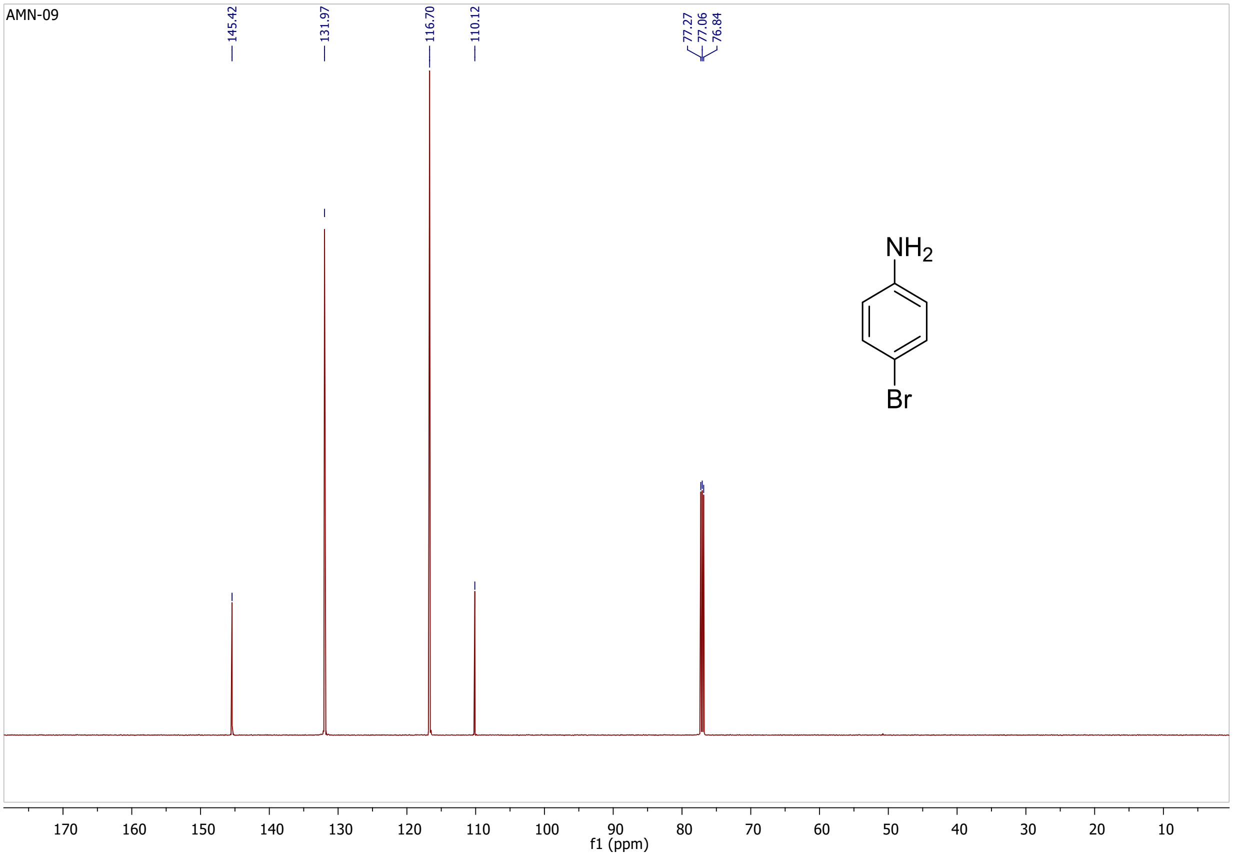

^{1}H NMR of 3-chloroaniline (2c)

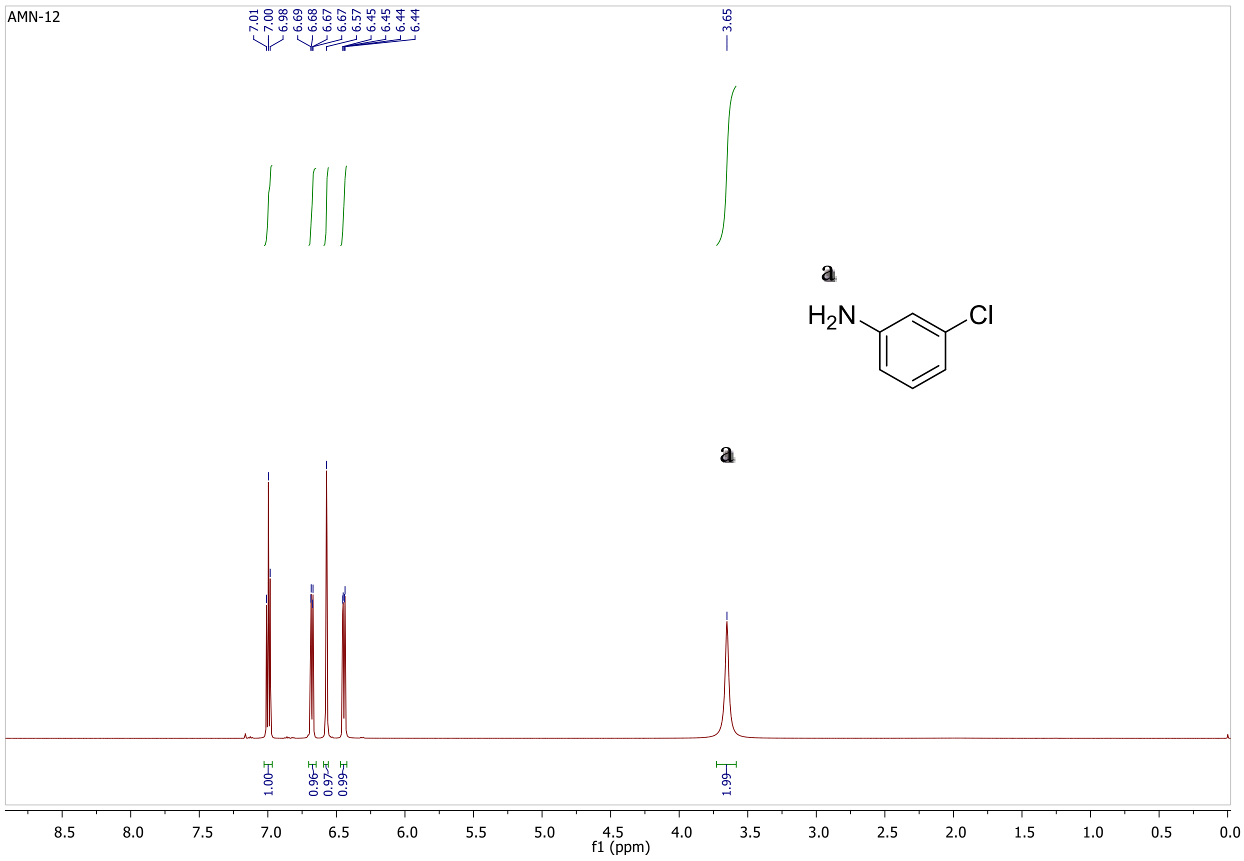

^{13}C NMR of 3-chloroaniline (2c)

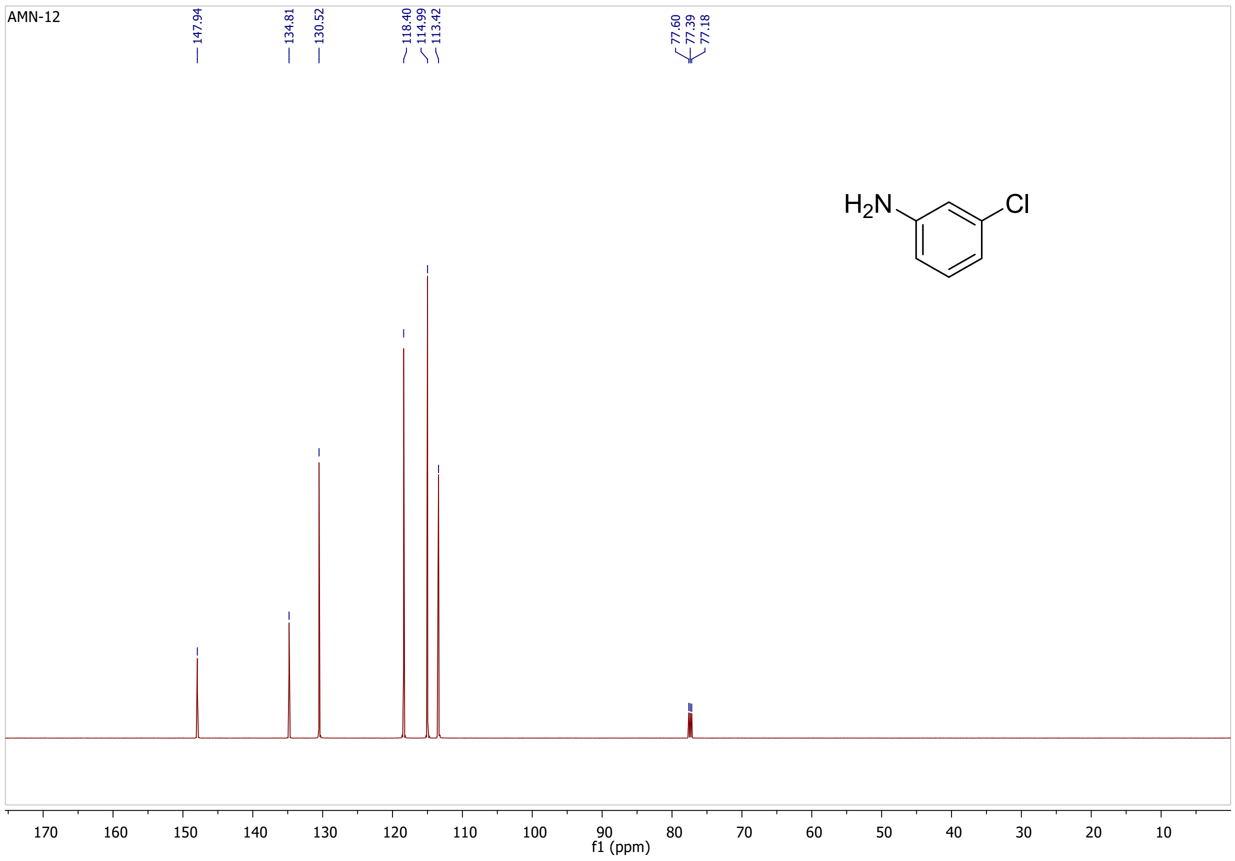

^{1}H NMR of 4-aminobenzoic acid (2d)

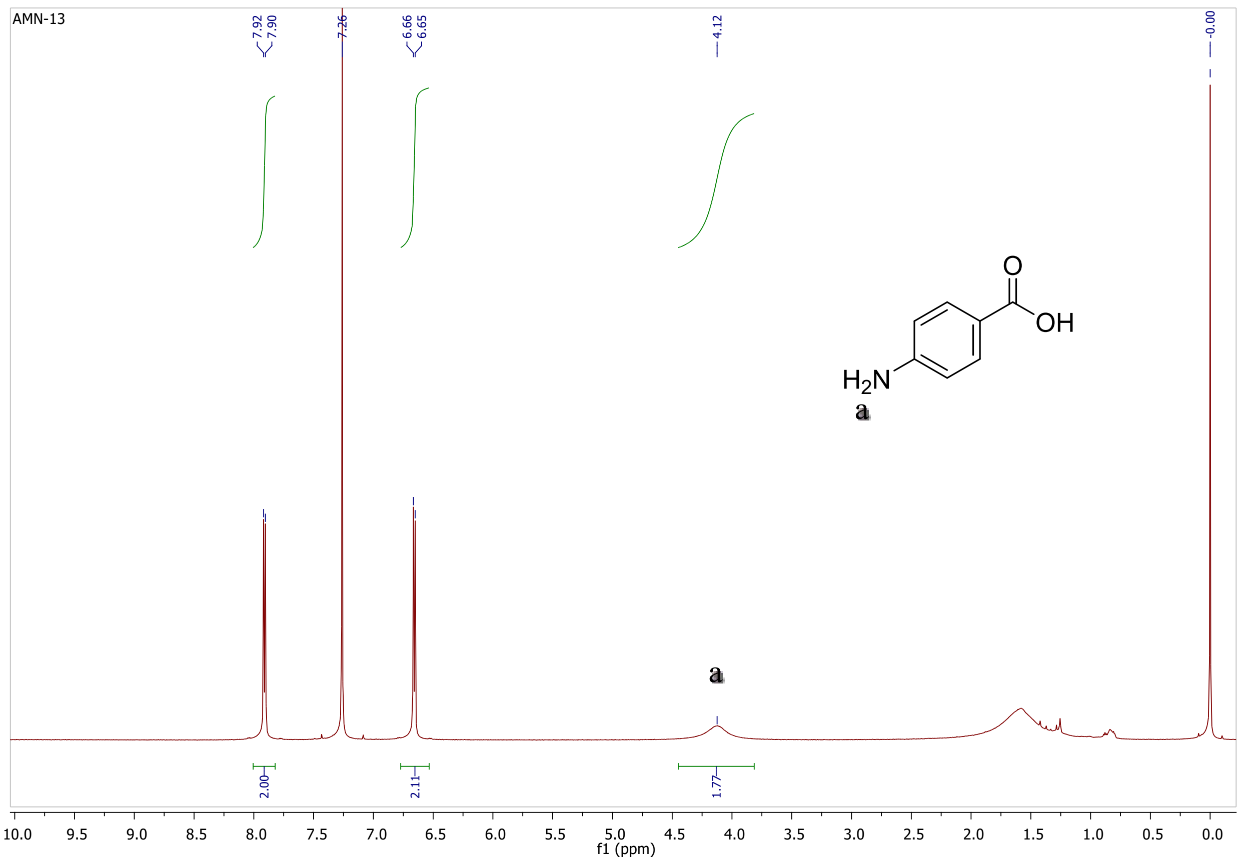

^{13}C NMR of 4-aminobenzoic acid (2d)

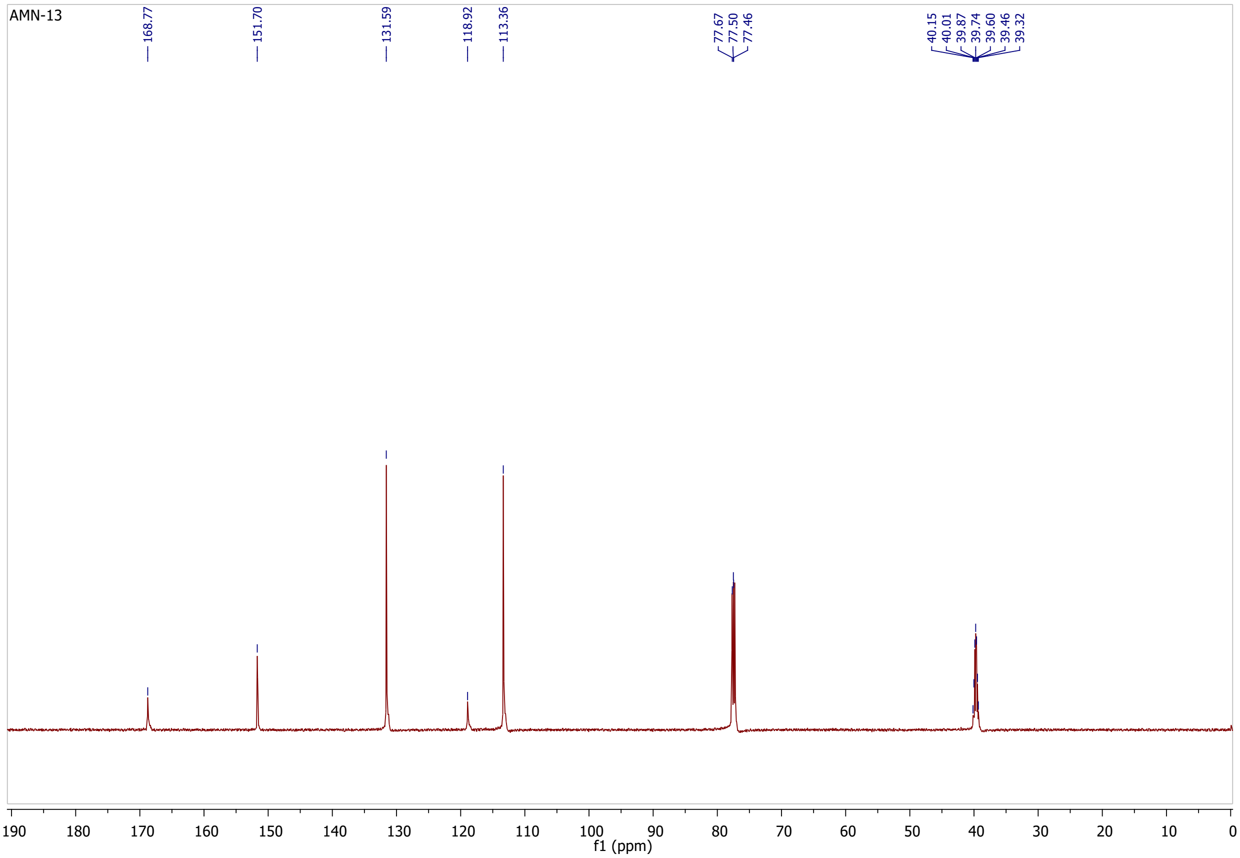

^{1}H NMR of 3-aminobenzaldehyde (2e)

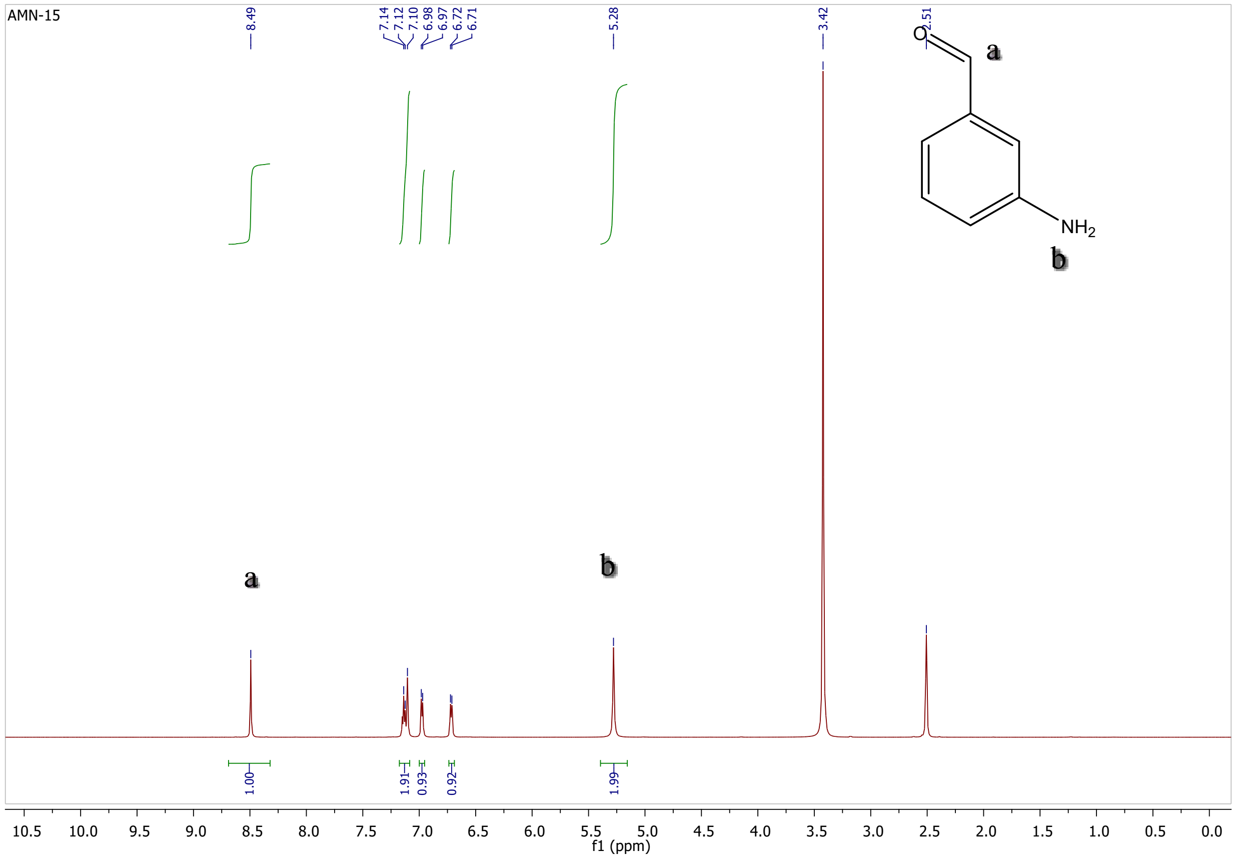

^{13}C NMR of 3-aminobenzaldehyde (2e)

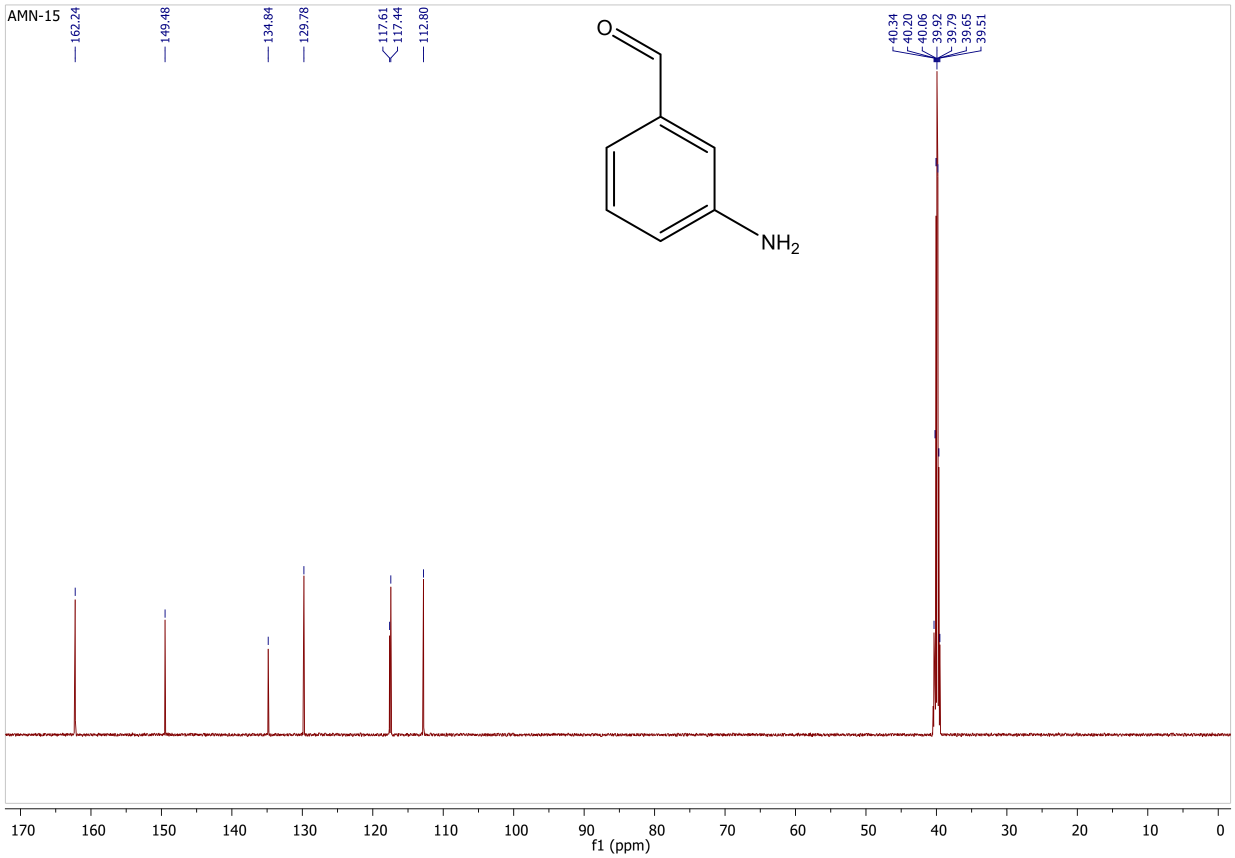

HRMS of 3-aminobenzaldehyde (2e)

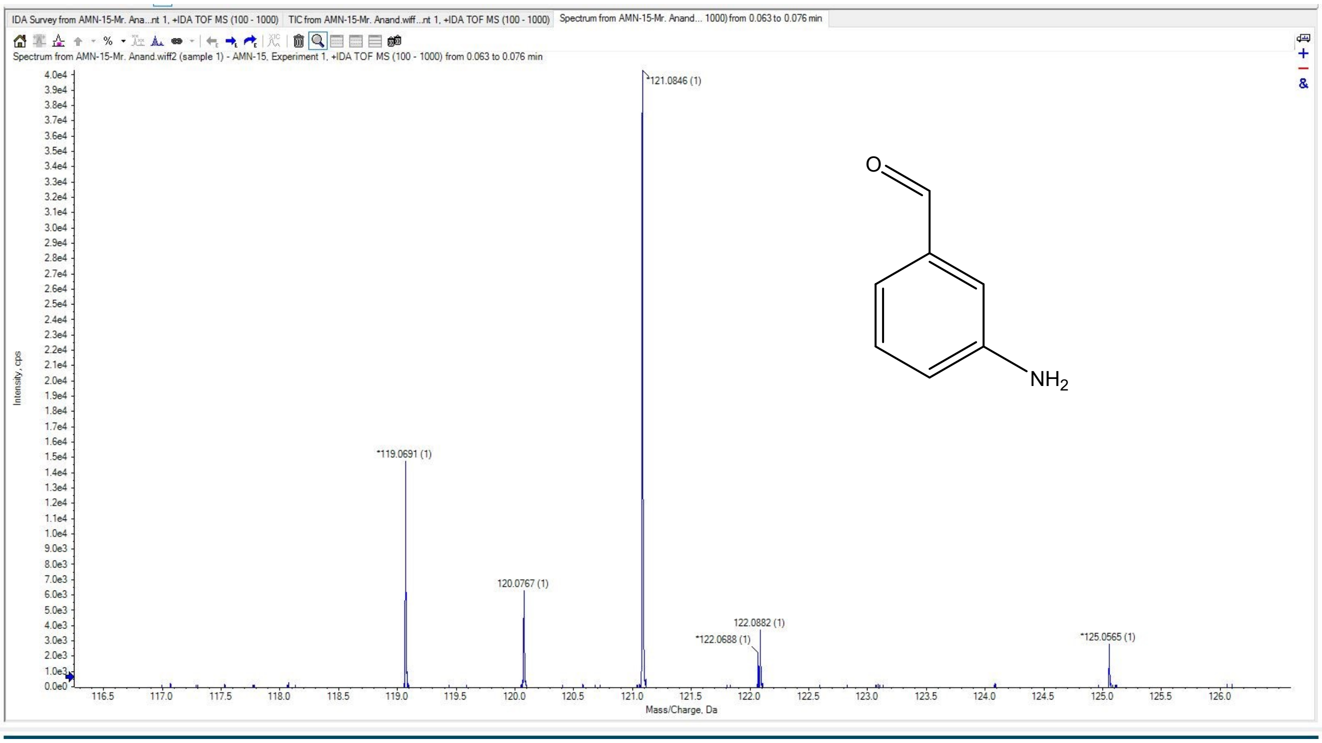

^{1}H NMR of 4-chloroaniline (2f)

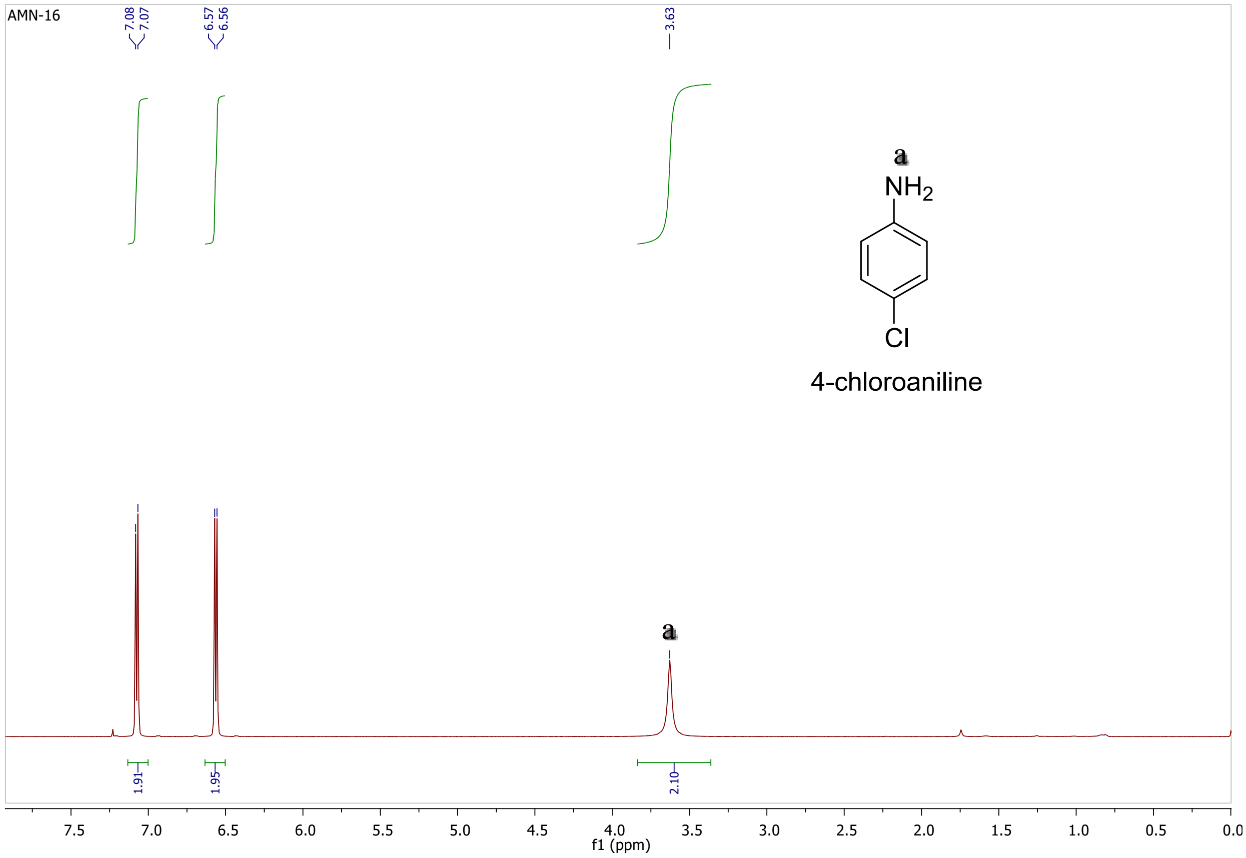

^{13}C NMR of 4-chloroaniline (2f)

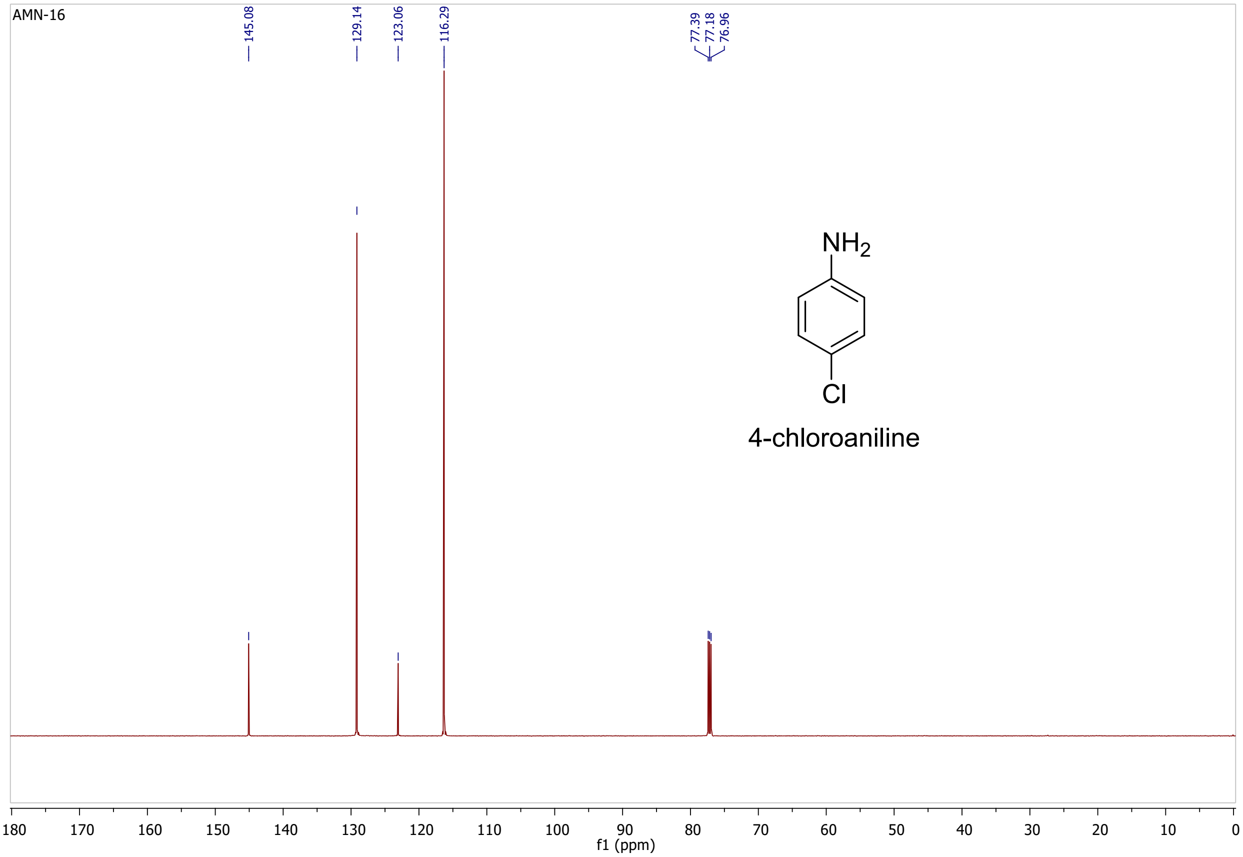

HRMS of 4-chloroaniline (2f)

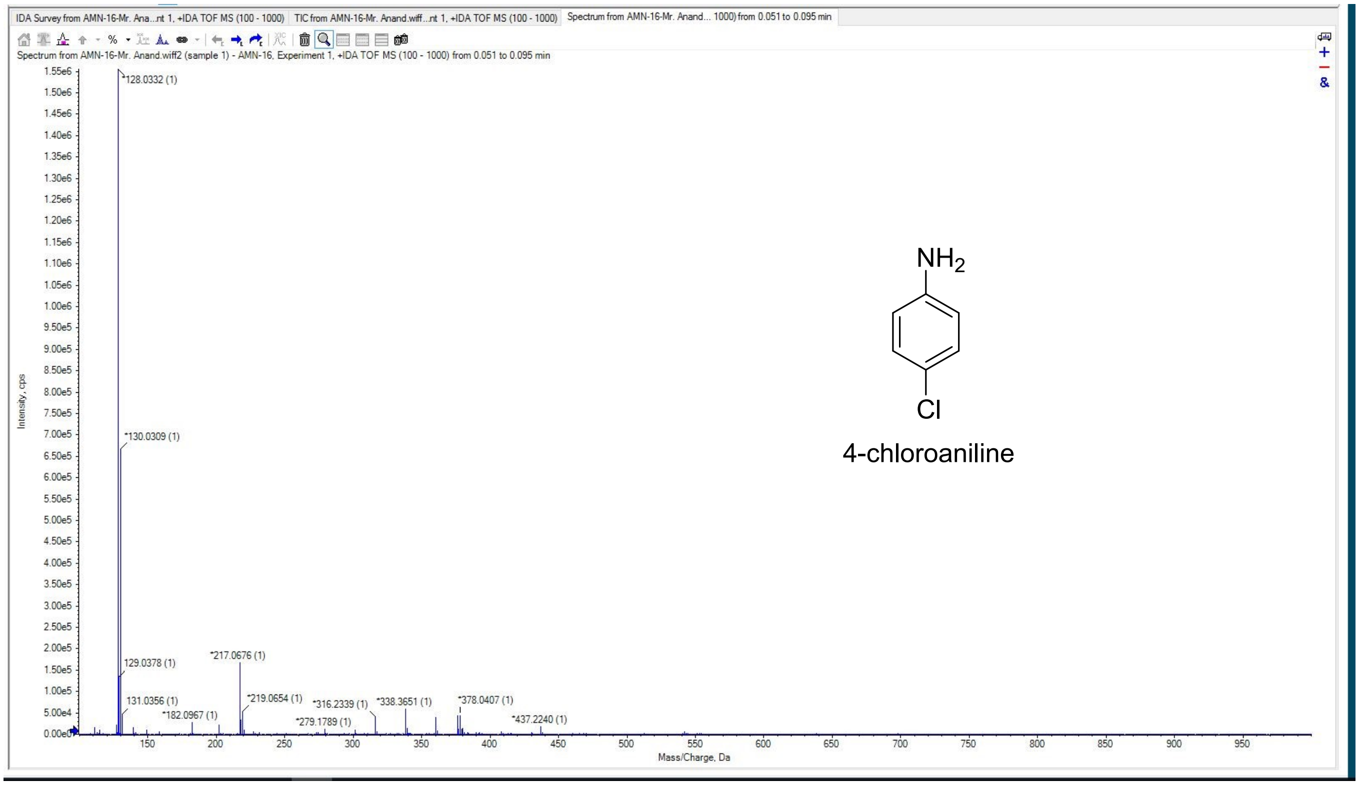

^{1}H NMR of benzene-1,2-diamine (2g)

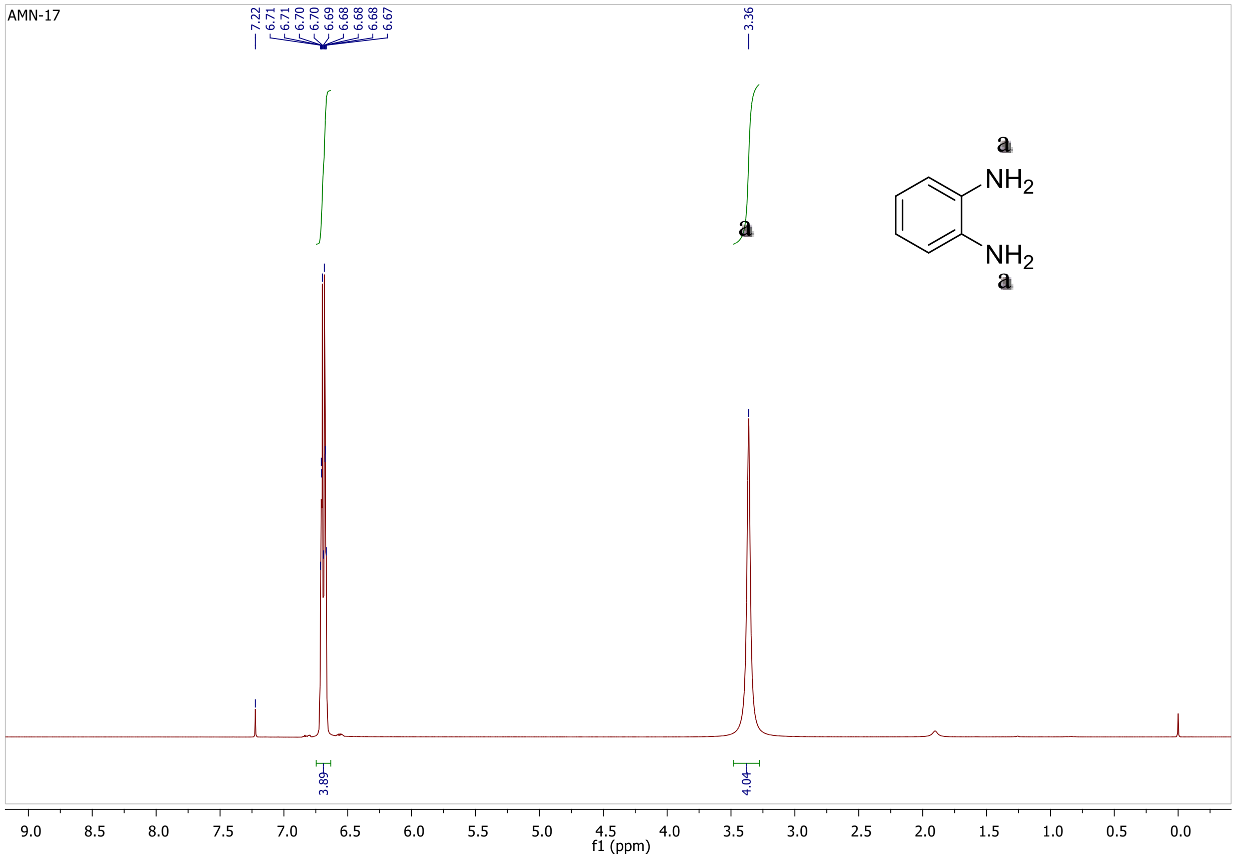

^{13}C NMR of benzene-1,2-diamine (2g)

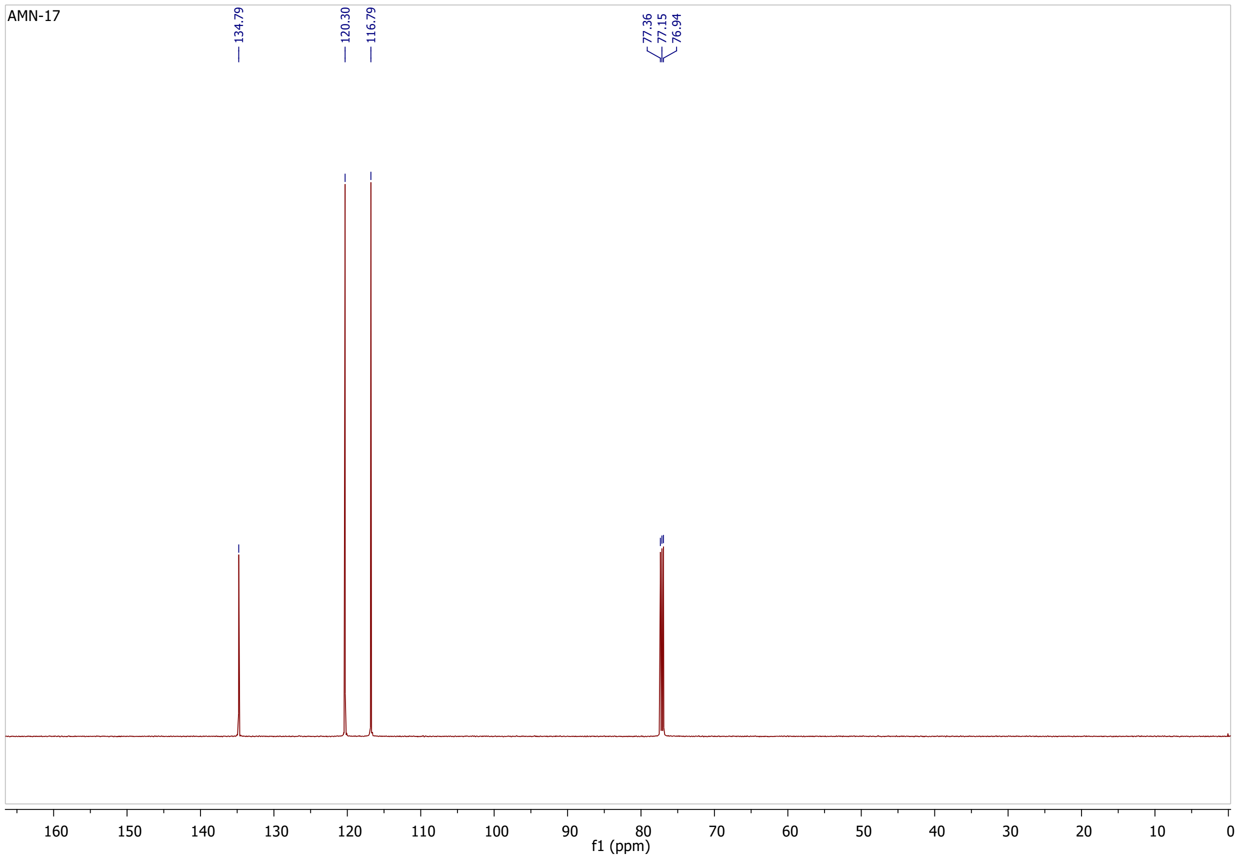

^{1}H NMR of 4-aminobenzaldehyde (2h)

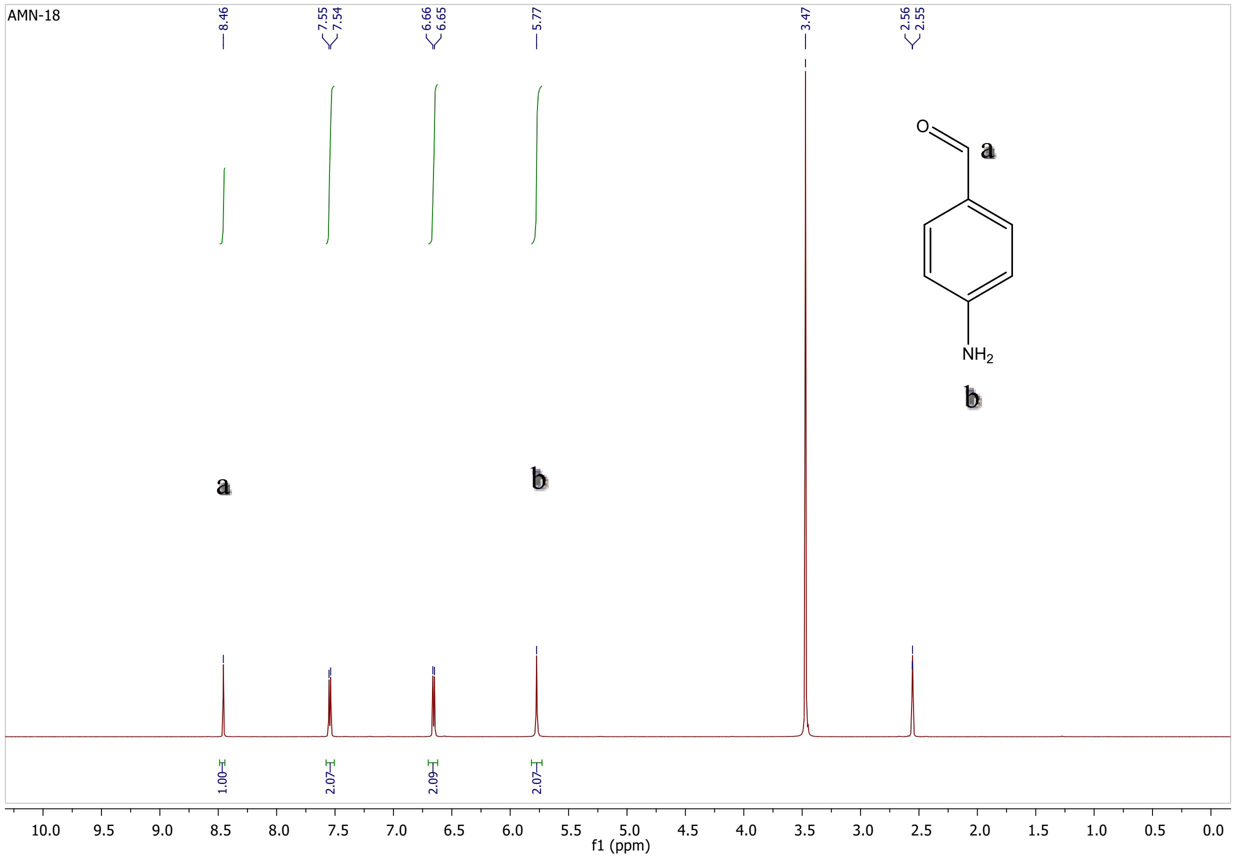

^{13}C NMR of 4-aminobenzaldehyde (2h)

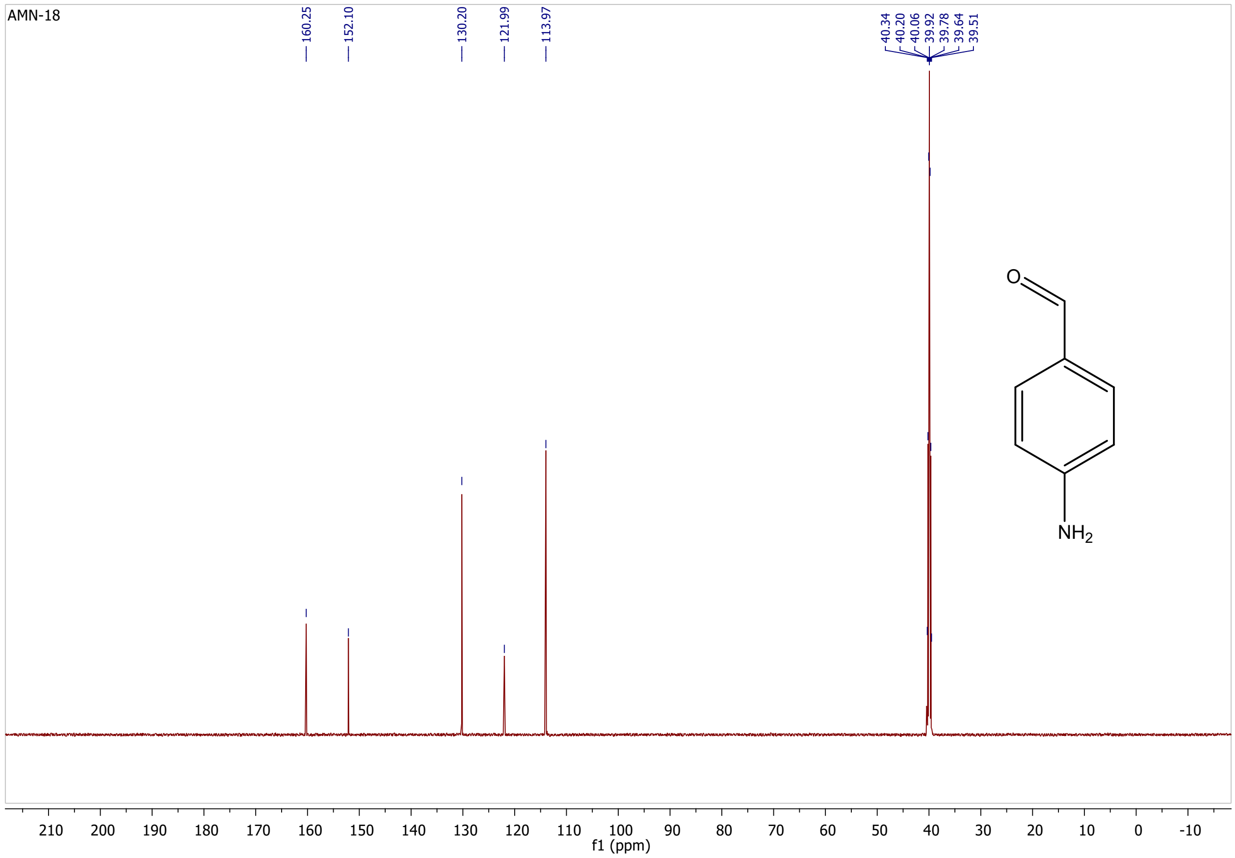

5. Physical data of synthesized 5-substituted-1*H*-tetrazole derivatives.

^{1}H NMR of 5-(4-chlorophenyl)-1H-tetrazole (5a)

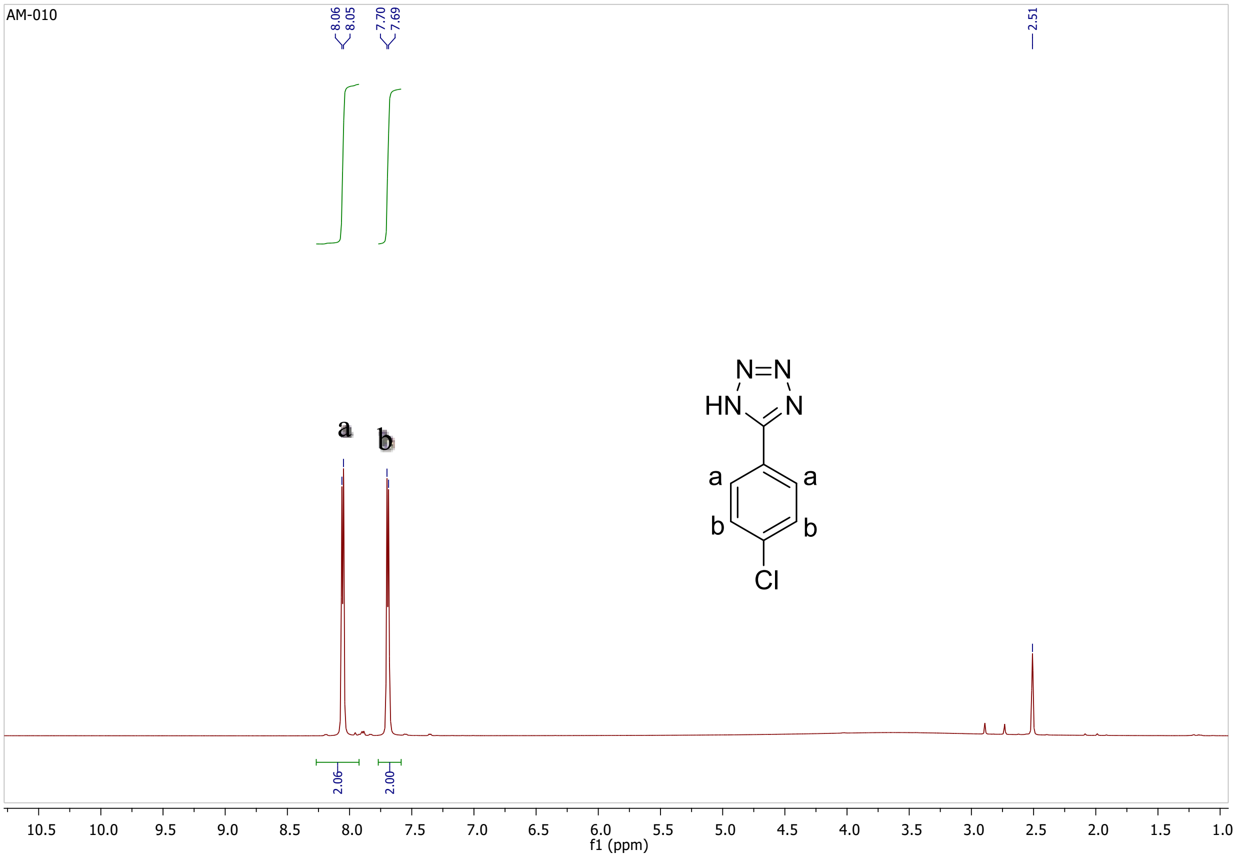

^{13}C NMR of 5-(4-chlorophenyl)-1H-tetrazole (5a)

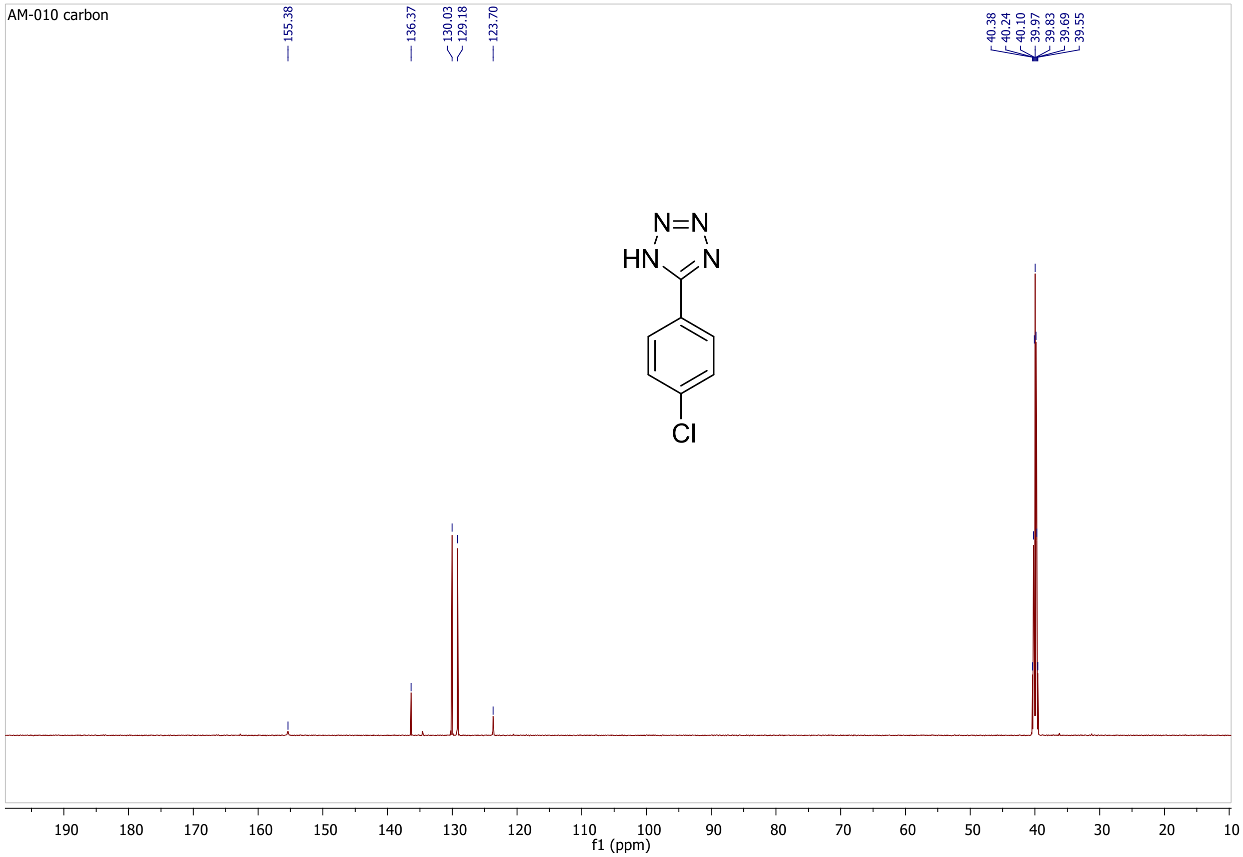

HRMS of 5-(4-chlorophenyl)-1H-tetrazole (5a)

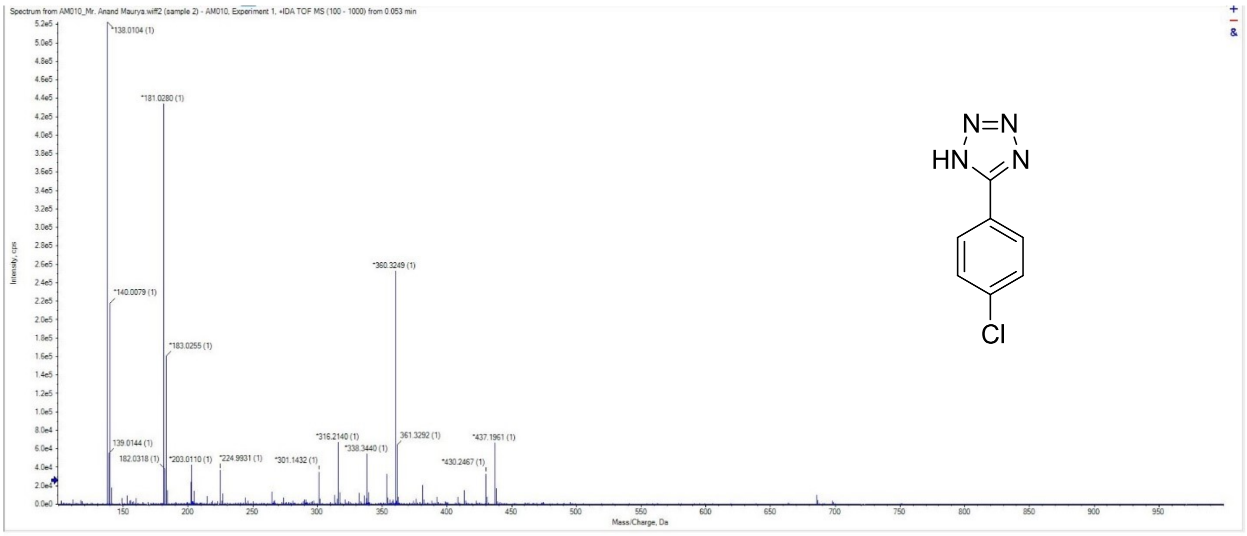

FT-IR of 5-(4-chlorophenyl)-1H-tetrazole (5a)

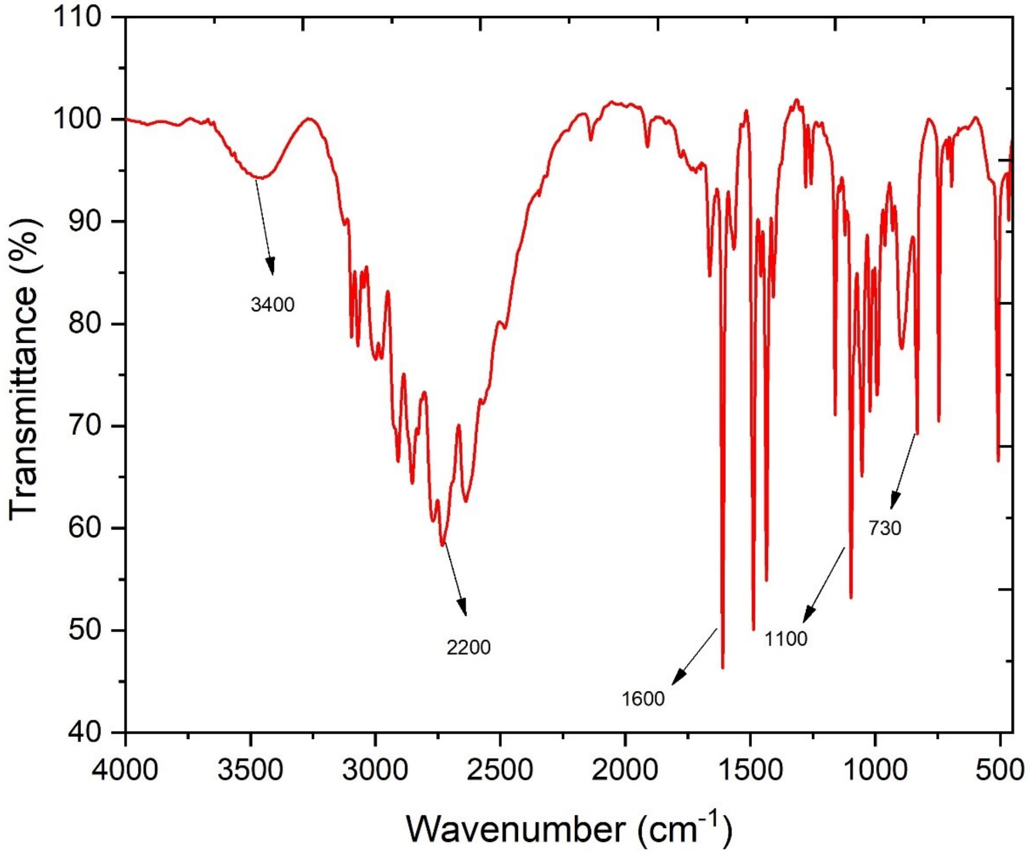

^{1}H NMR of 5-(4-iodophenyl)-1H-tetrazole (5b)

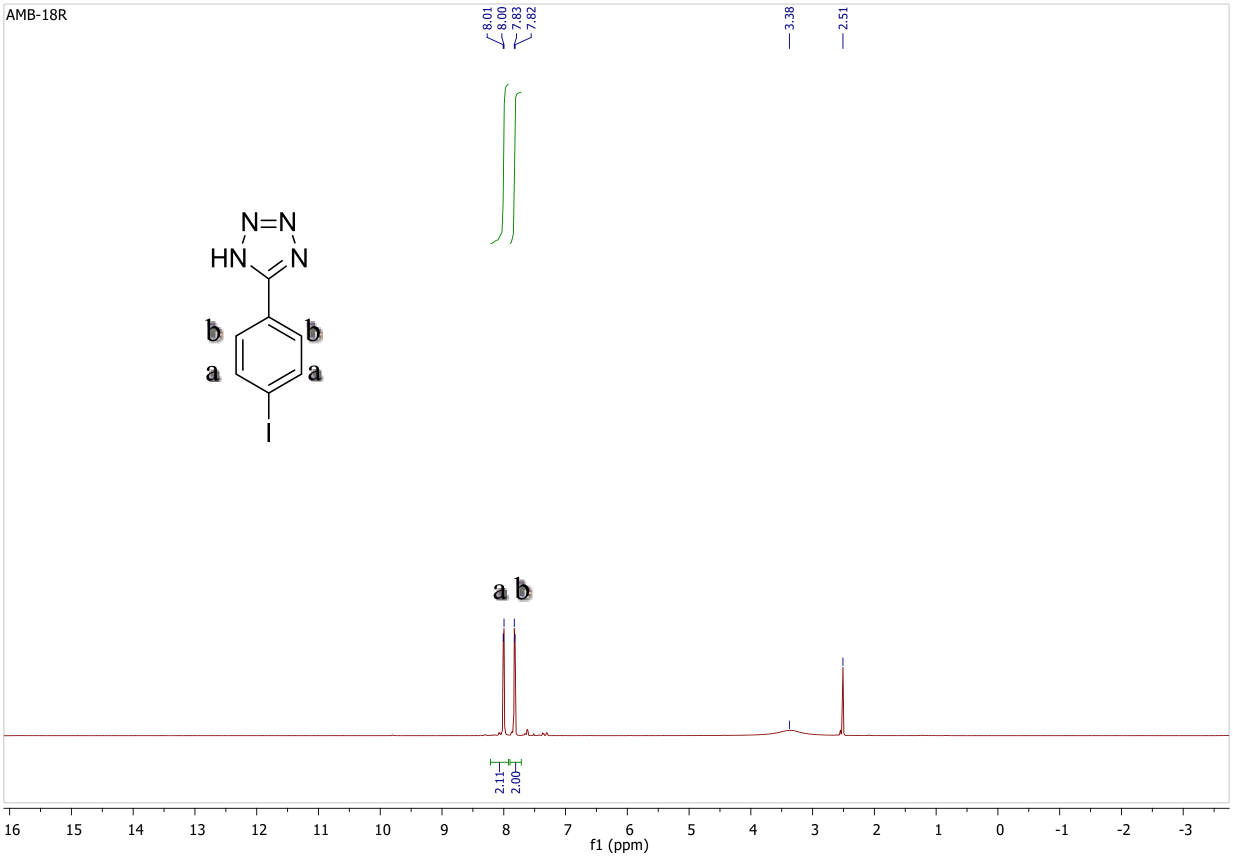

^{13}C NMR of 5-(4-iodophenyl)-1H-tetrazole (5b)

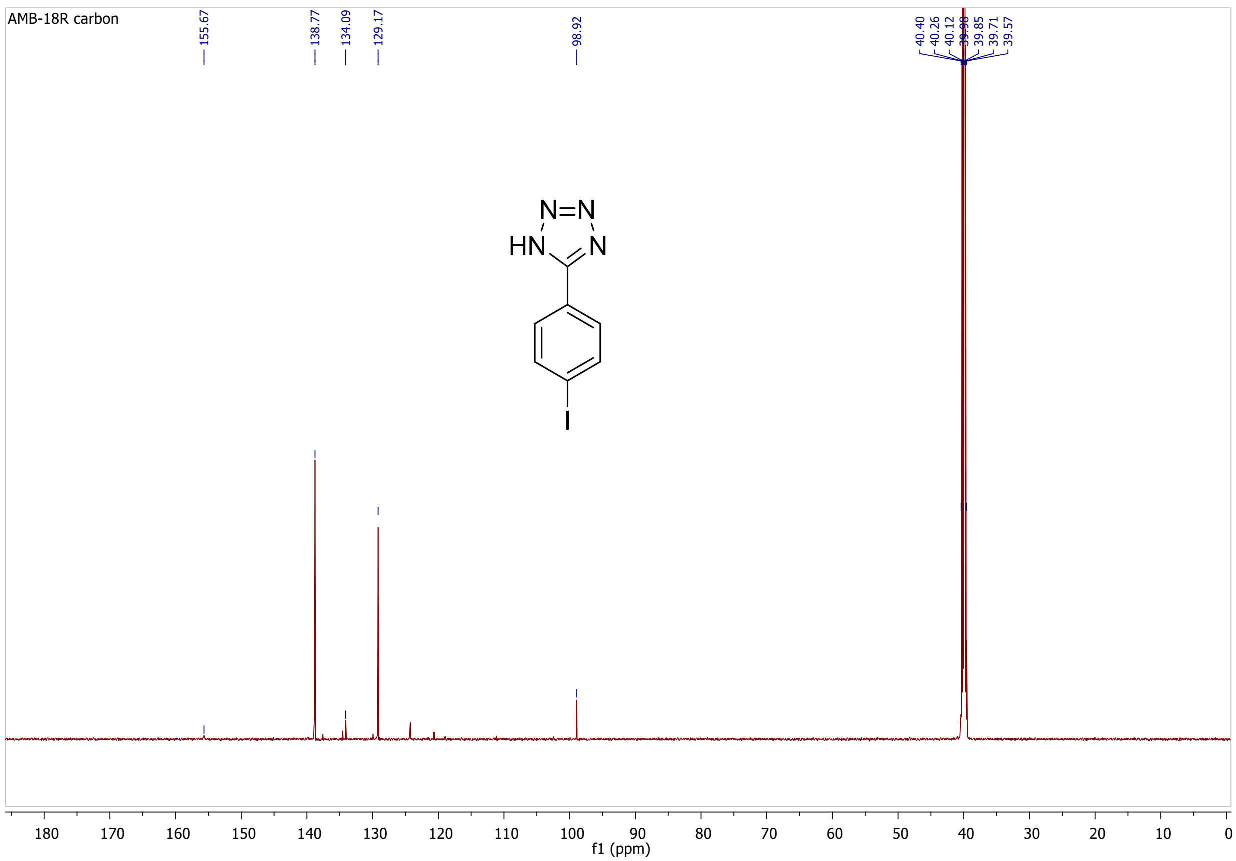

FT-IR of 5-(4-iodophenyl)-1H-tetrazole (5b)

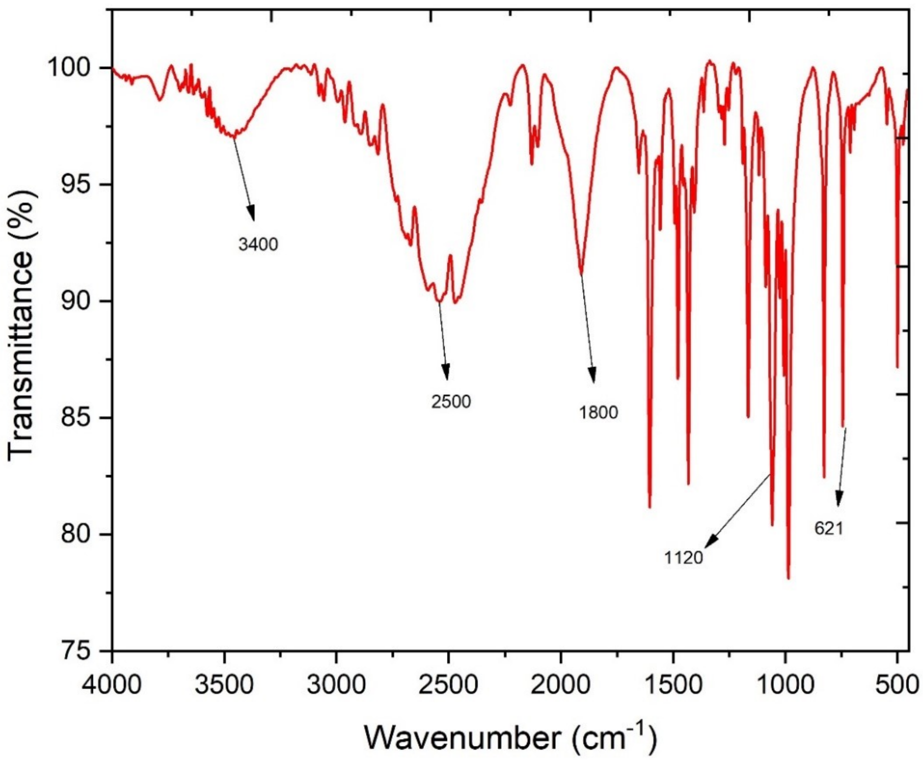

^{1}H NMR of 5-(3-bromo-4-fluorophenyl)-1H-tetrazole (5c)

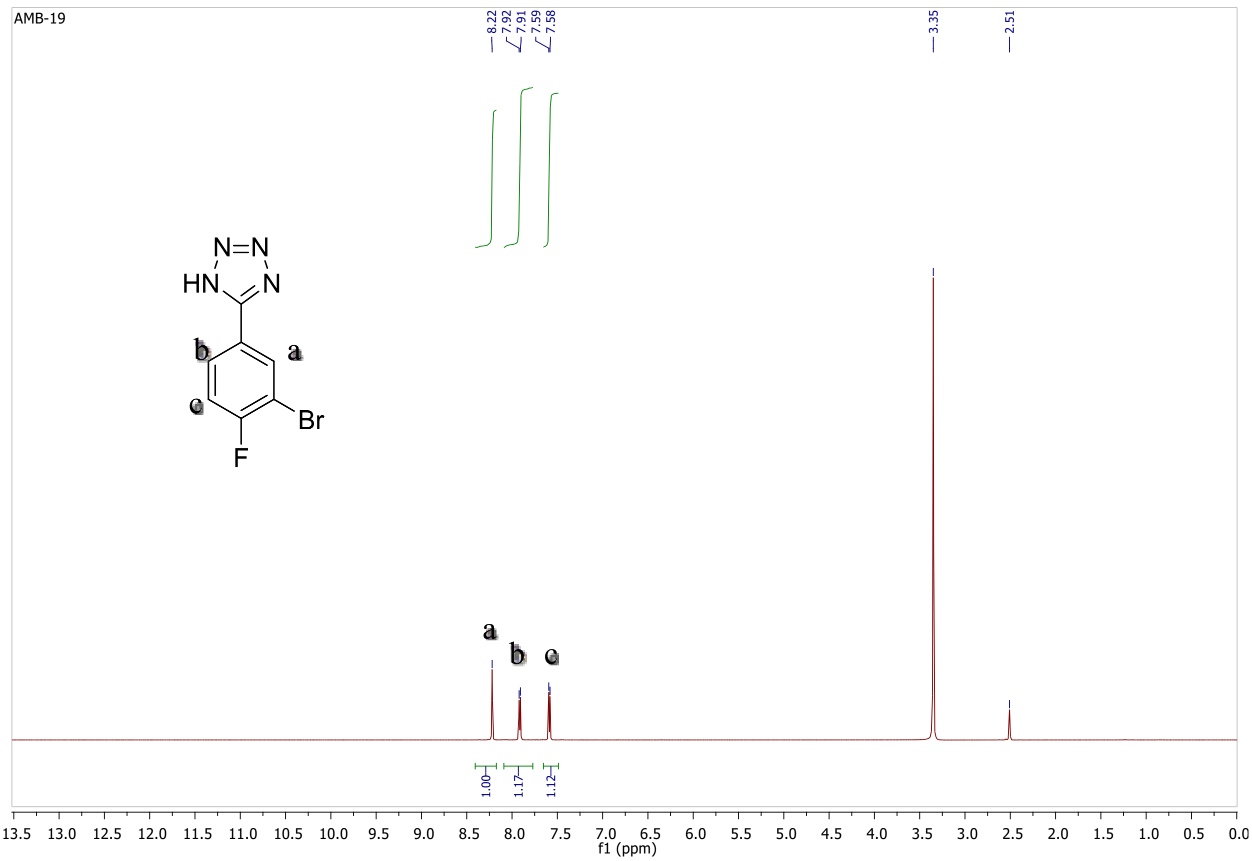

^{13}C NMR of 5-(3-bromo-4-fluorophenyl)-1H-tetrazole (5c)

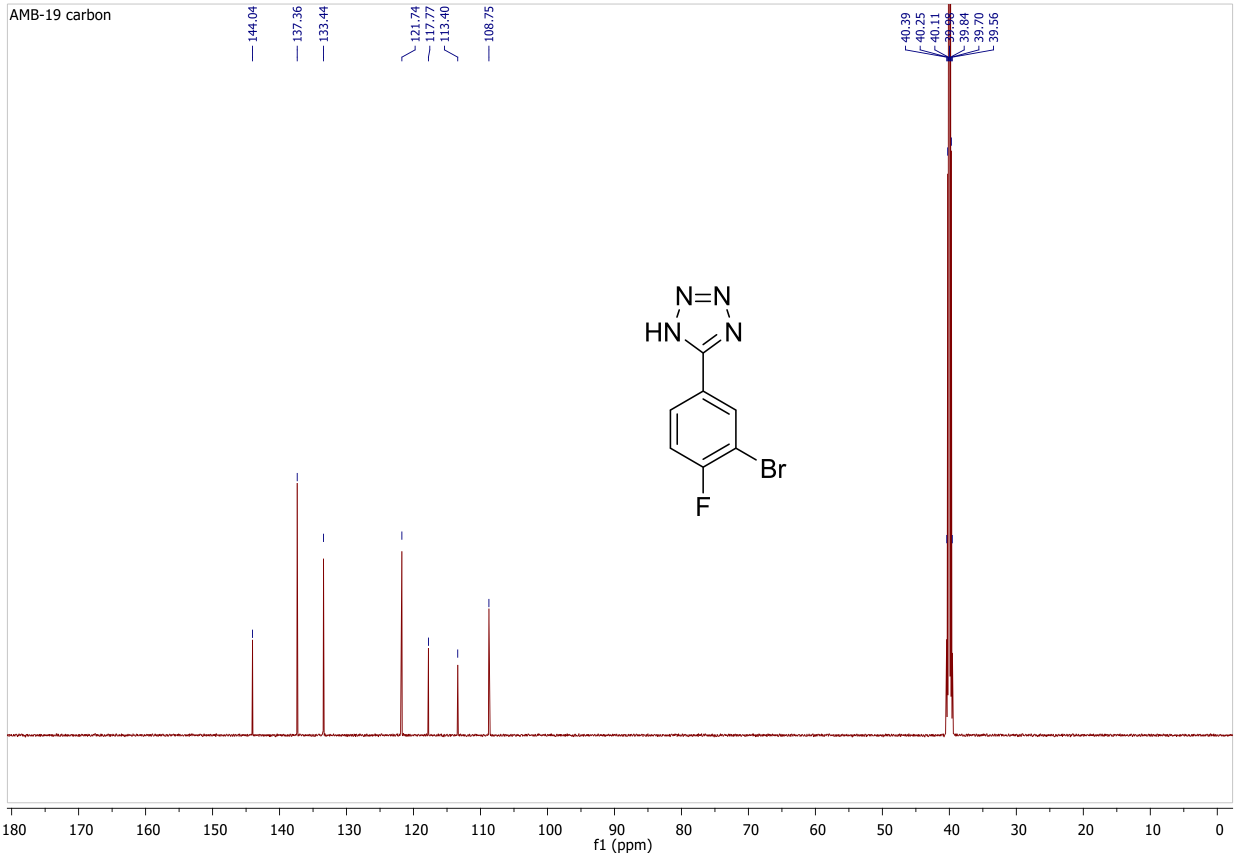

FT-IR of 5-(3-bromo-4-fluorophenyl)-1H-tetrazole (5c)

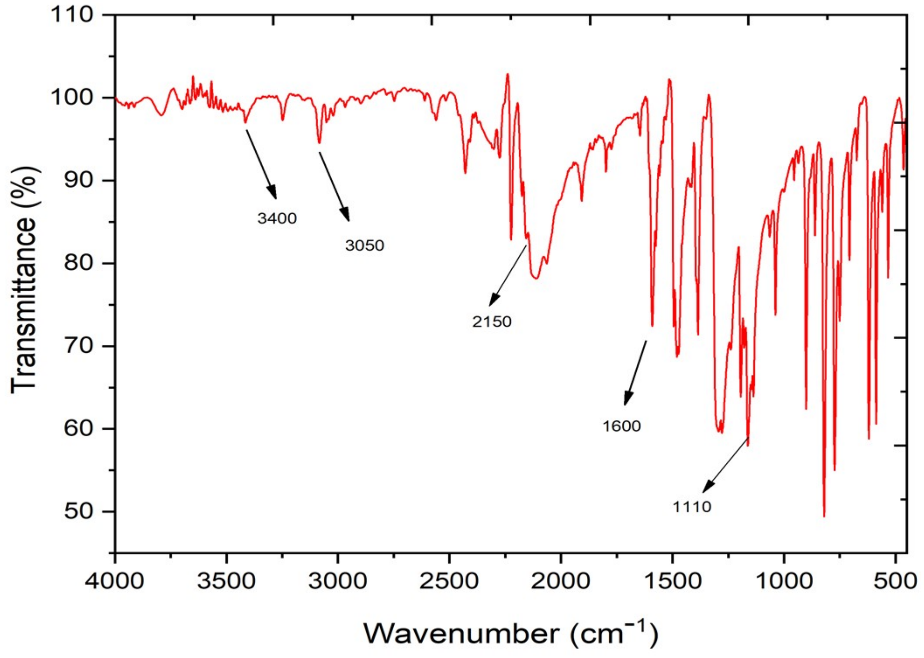

^{1}H NMR of 5-(2,4-difluorophenyl)-1H-tetrazole (5d)

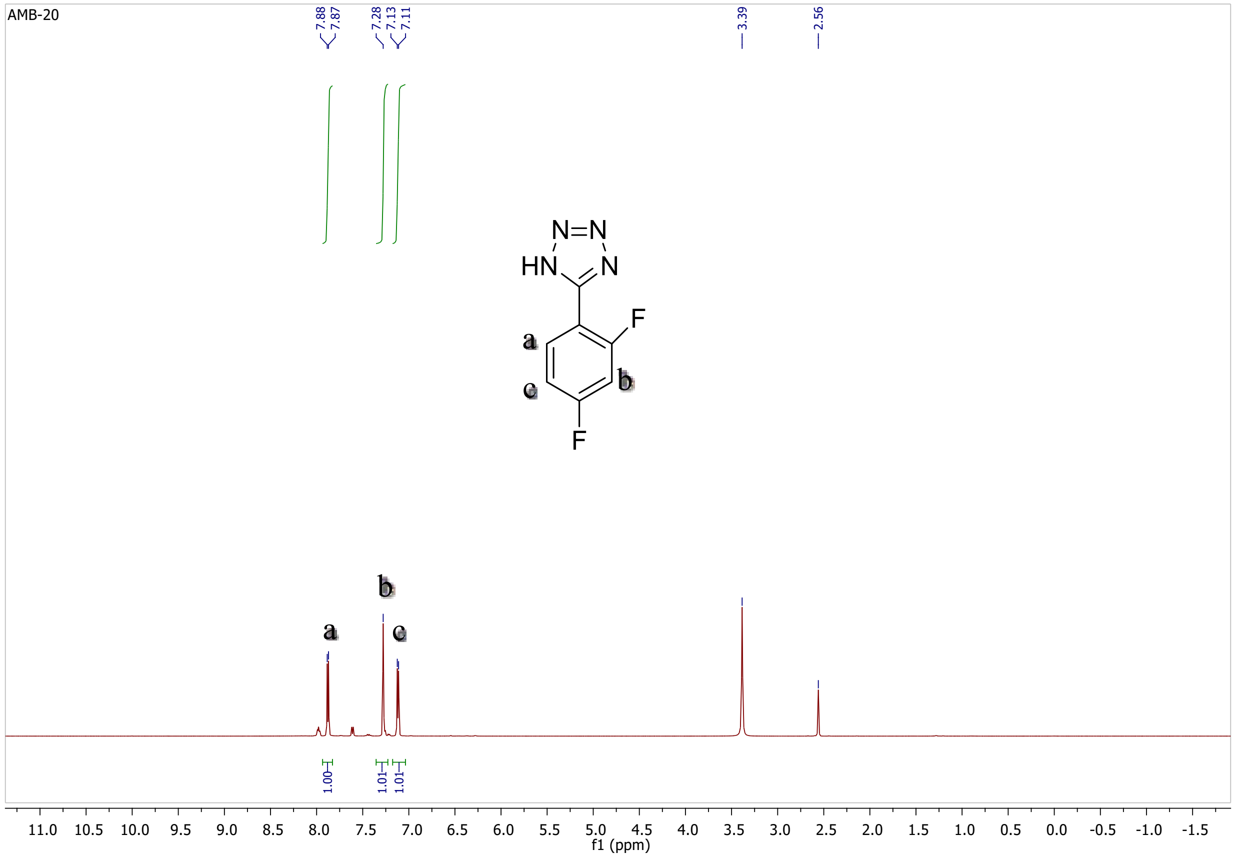

^{13}C NMR of 5-(2,4-difluorophenyl)-1H-tetrazole (5d)

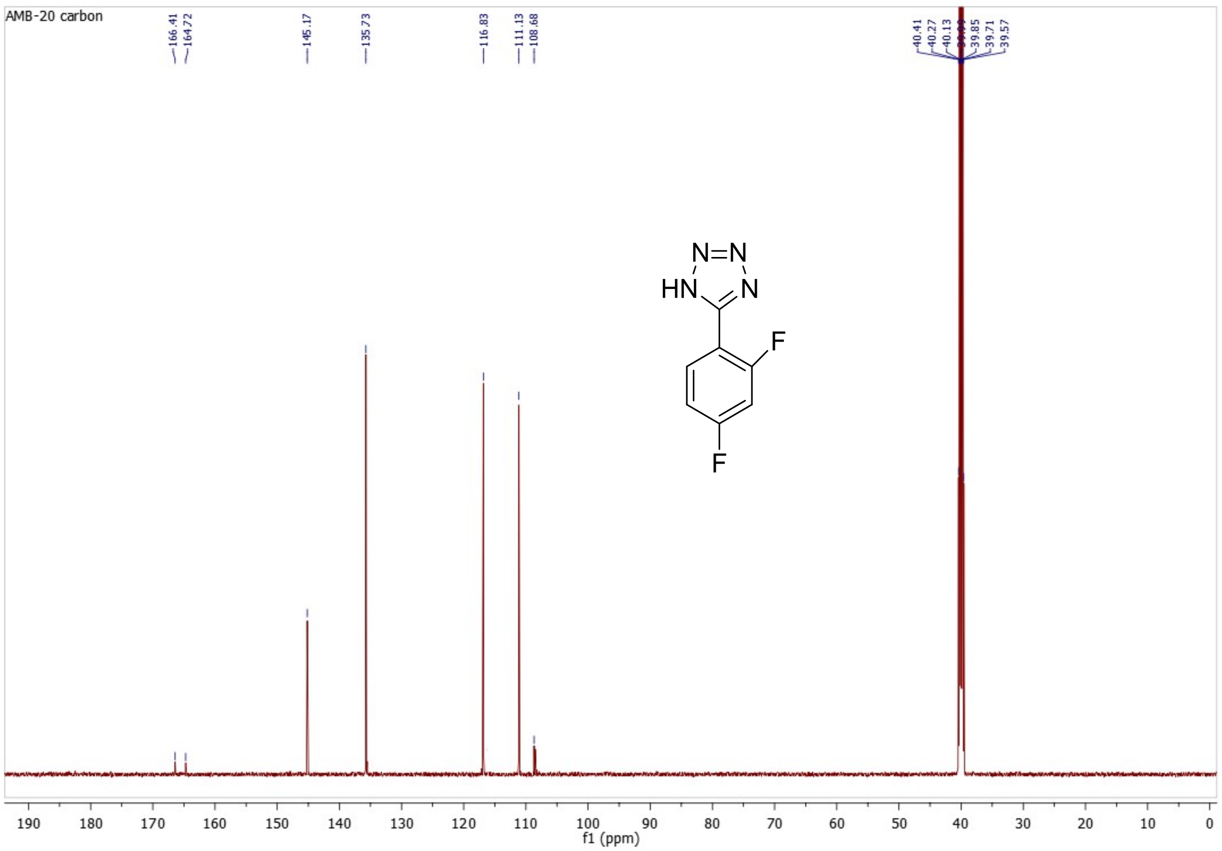

FT-IR of 5-(2,4-difluorophenyl)-1H-tetrazole (5d)

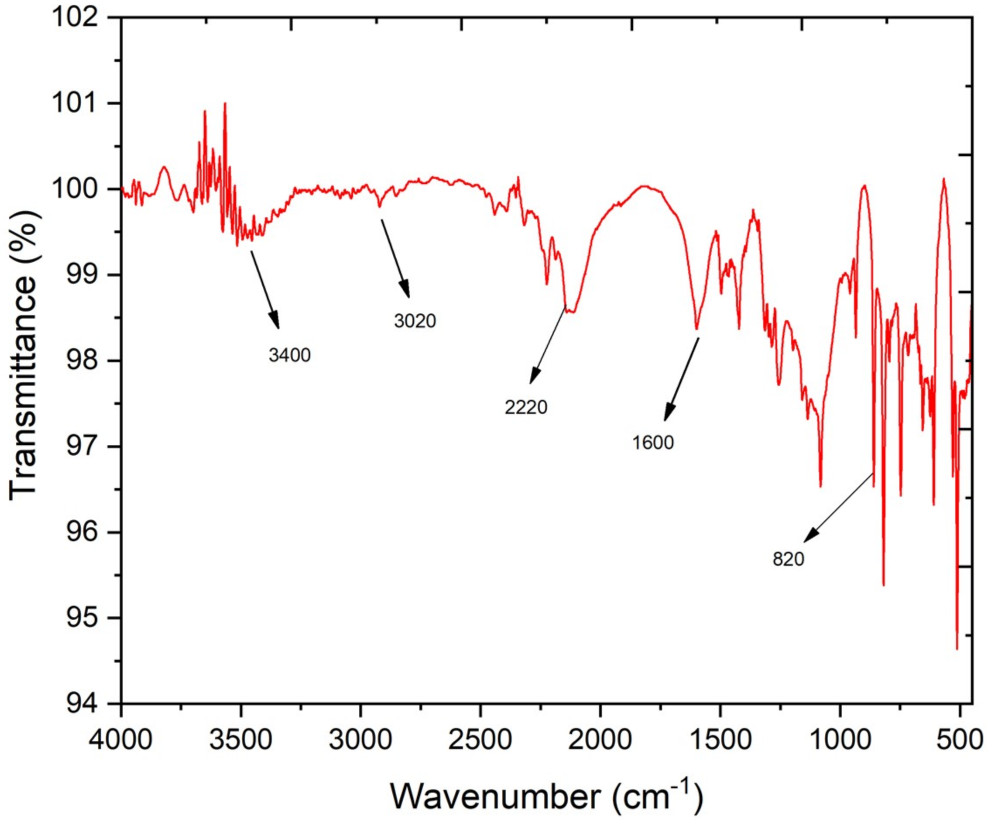

^{1}H NMR of 5-(2-fluorophenyl)-1H-tetrazole (5e)

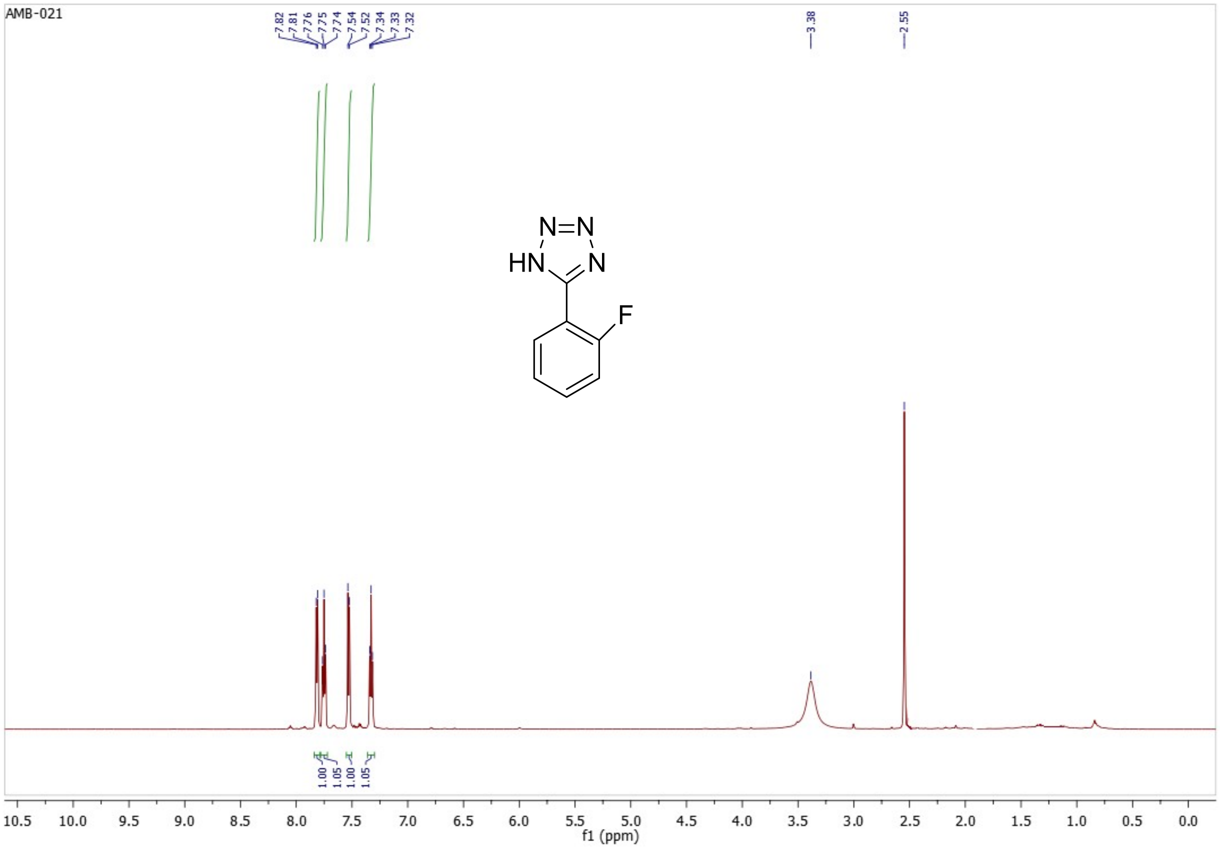

^{13}C NMR of 5-(2-fluorophenyl)-1H-tetrazole (5e)

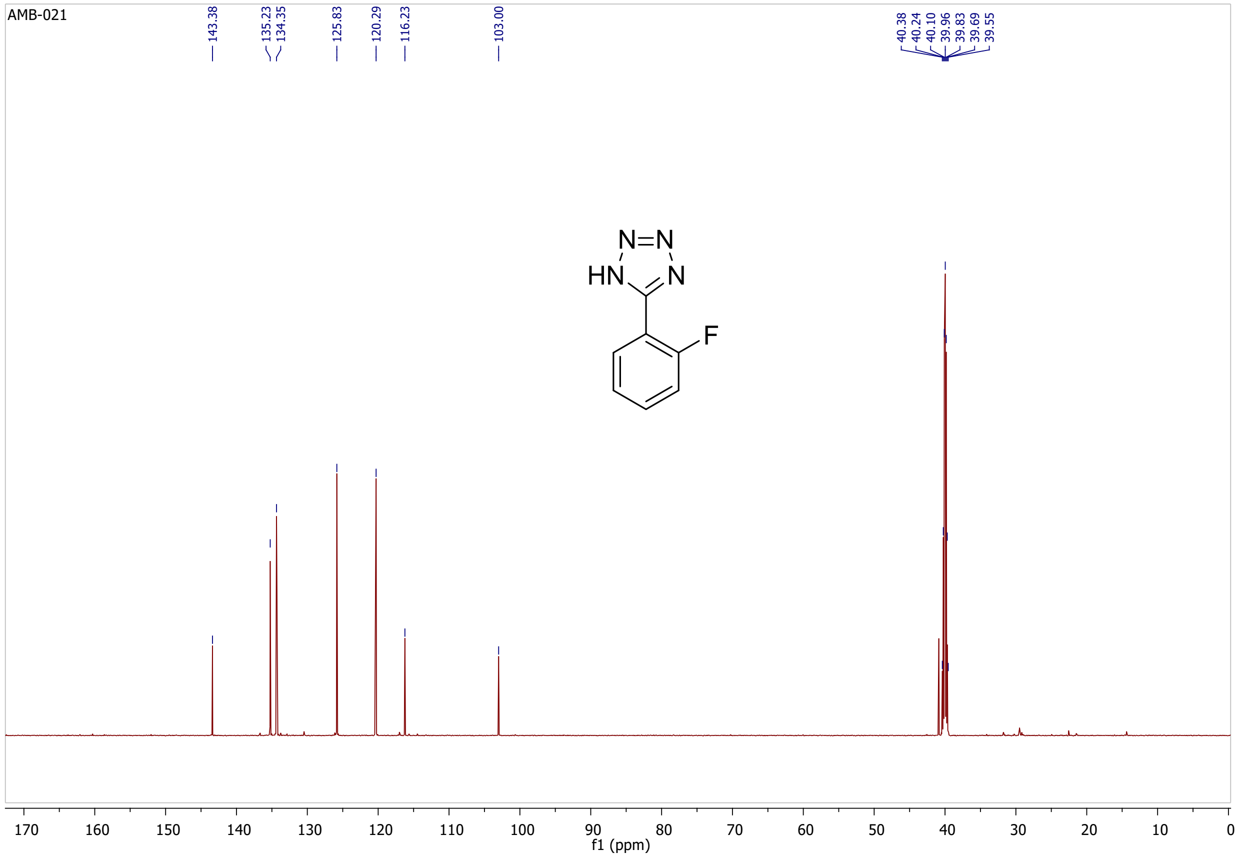

^{1}H NMR of 5-(2,6-dichlorophenyl)-1H-tetrazole (5f)

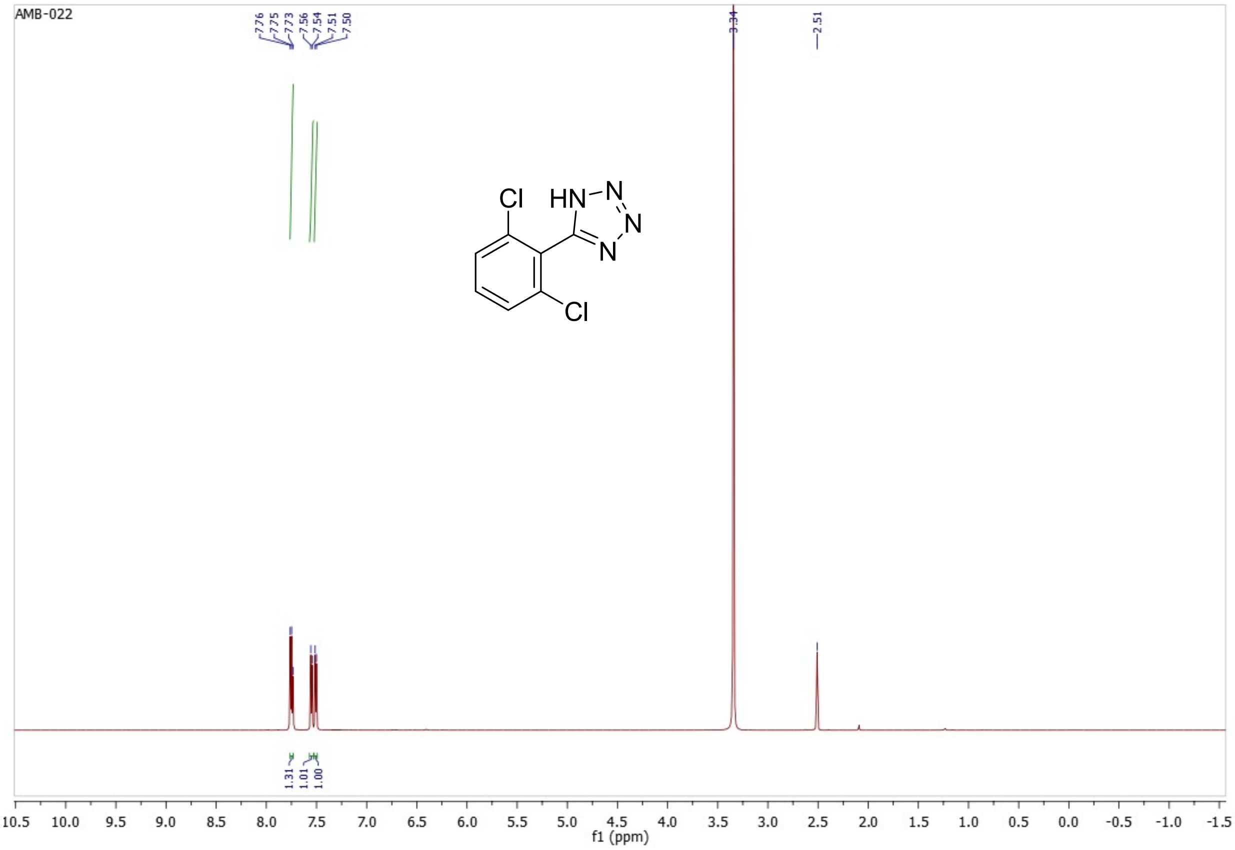

^{13}C NMR of 5-(2,6-dichlorophenyl)-1H-tetrazole (5f)

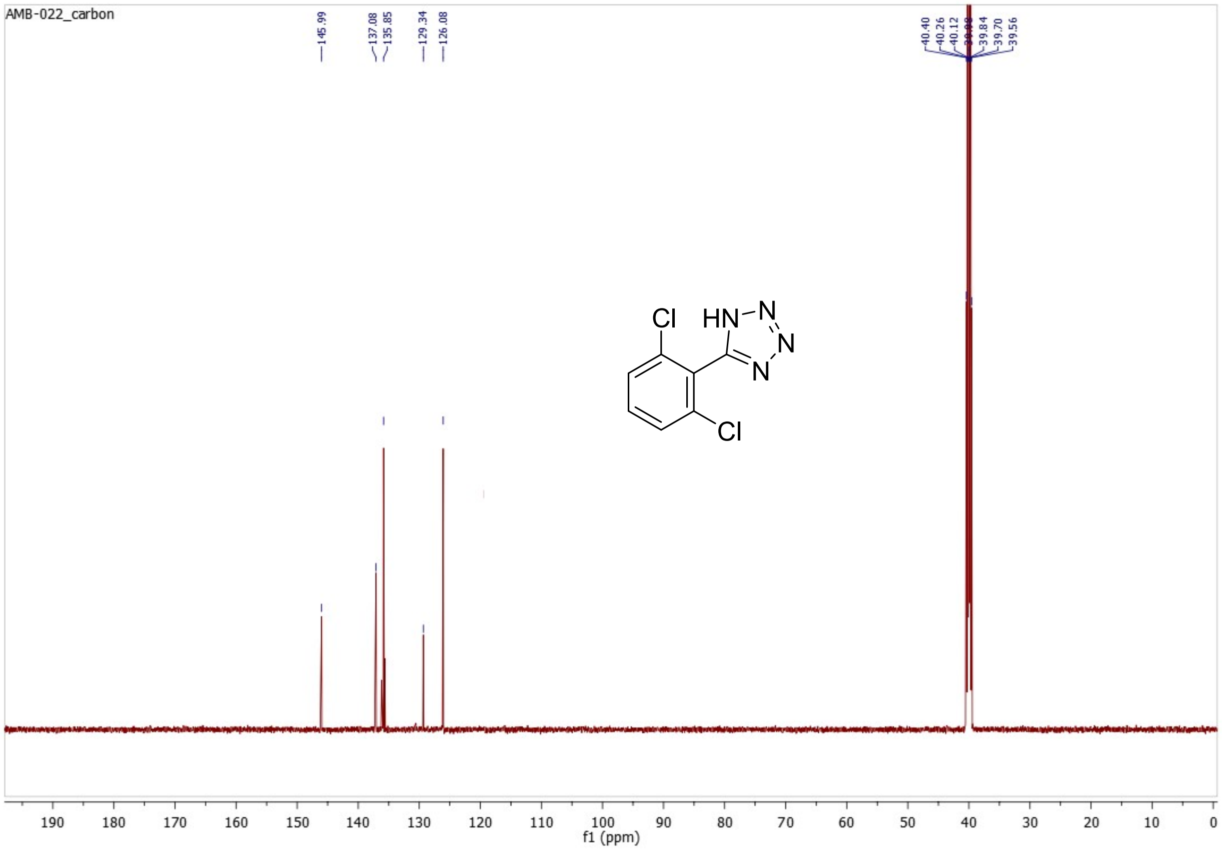

HRMS of 5-(2,6-dichlorophenyl)-1H-tetrazole (5f)

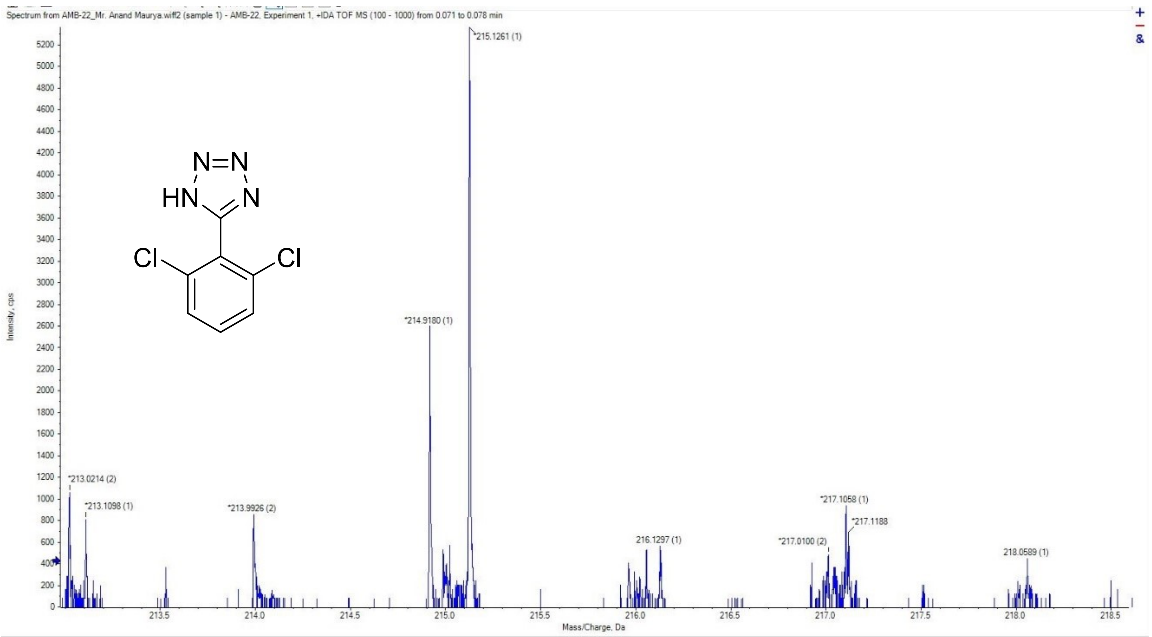

FT-IR of 5-(2,6-dichlorophenyl)-1H-tetrazole (5f)

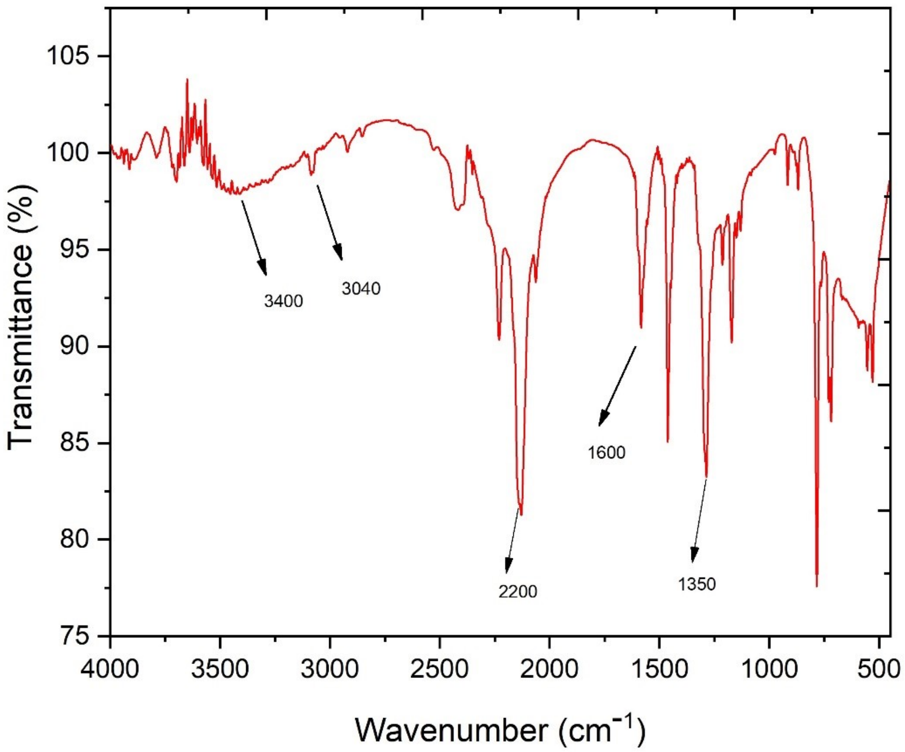

^{1}H NMR of 5-phenyl-1H-tetrazole (5g)

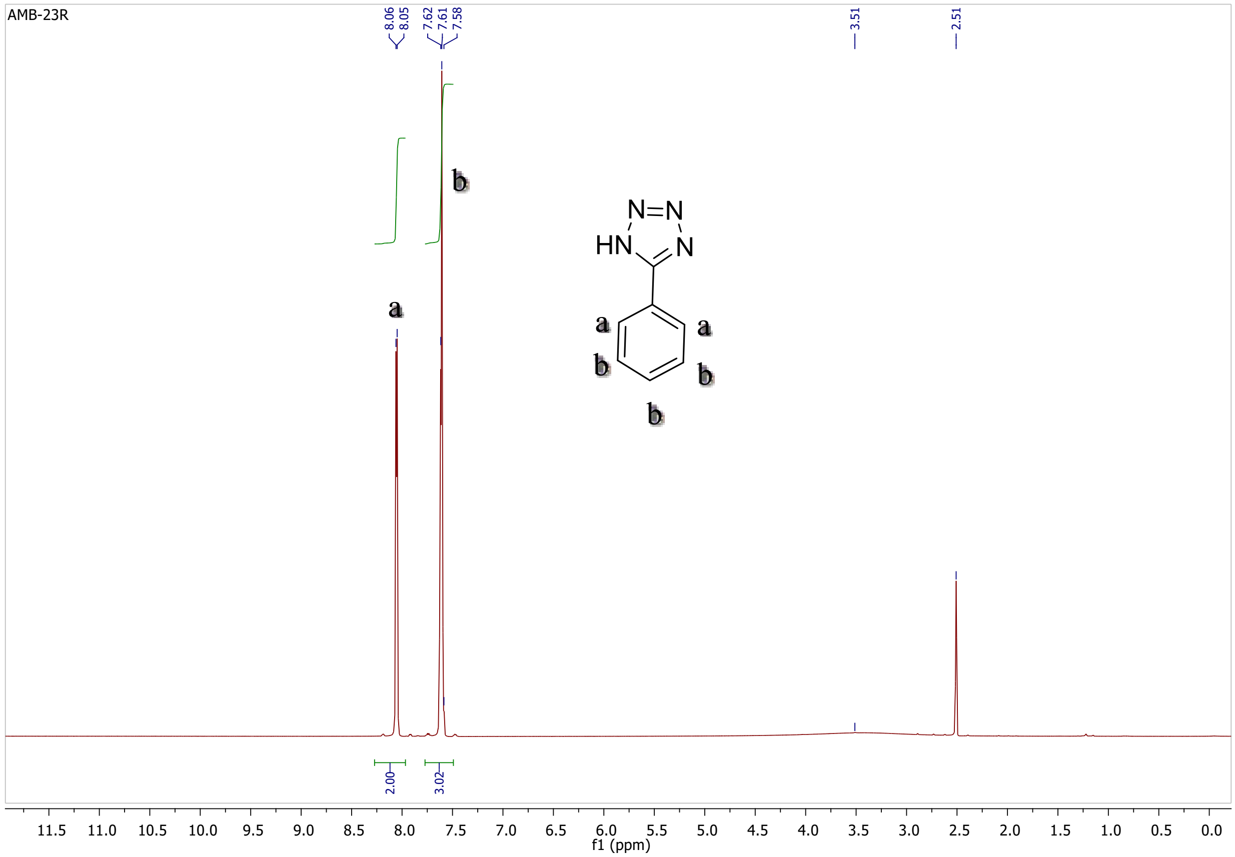

^{13}C NMR of 5-phenyl-1H-tetrazole (5g)

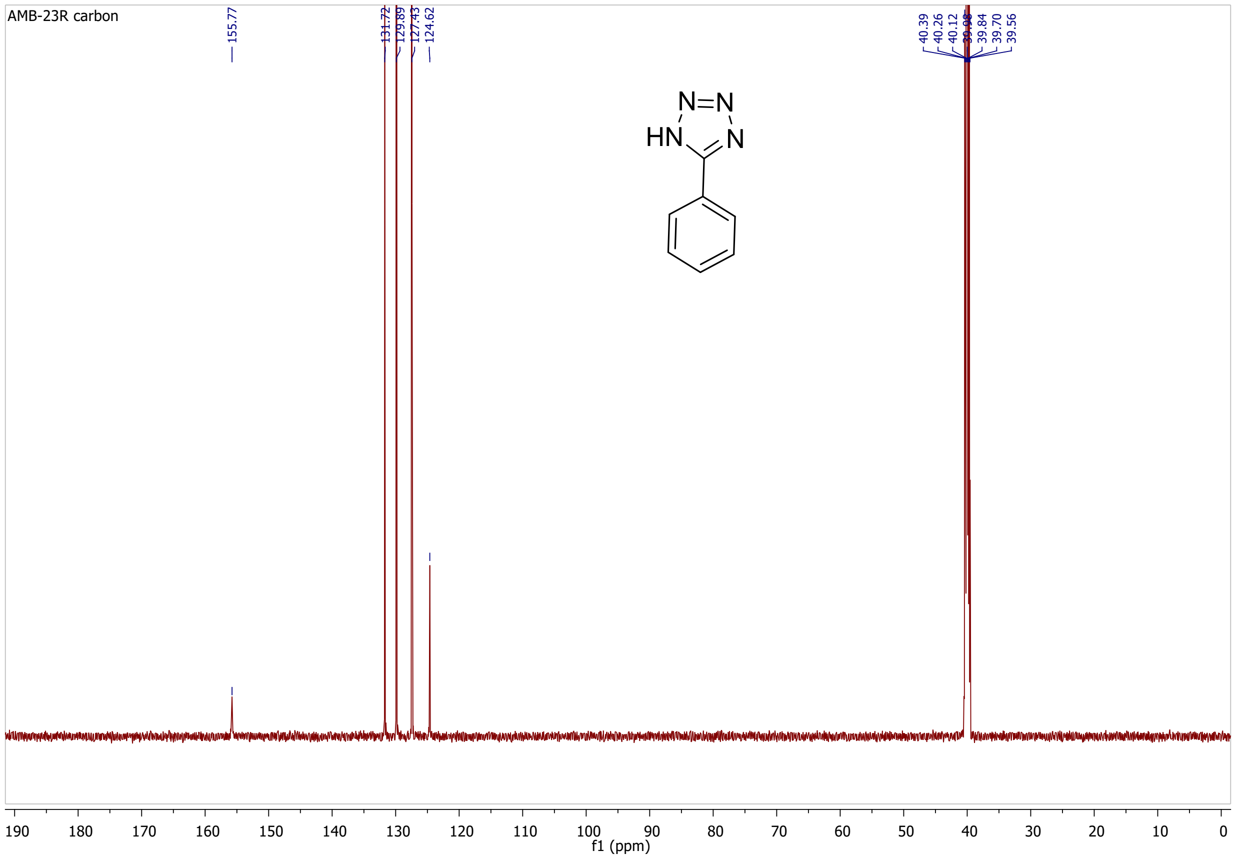

FT-IR of 5-phenyl-1H-tetrazole (5g)

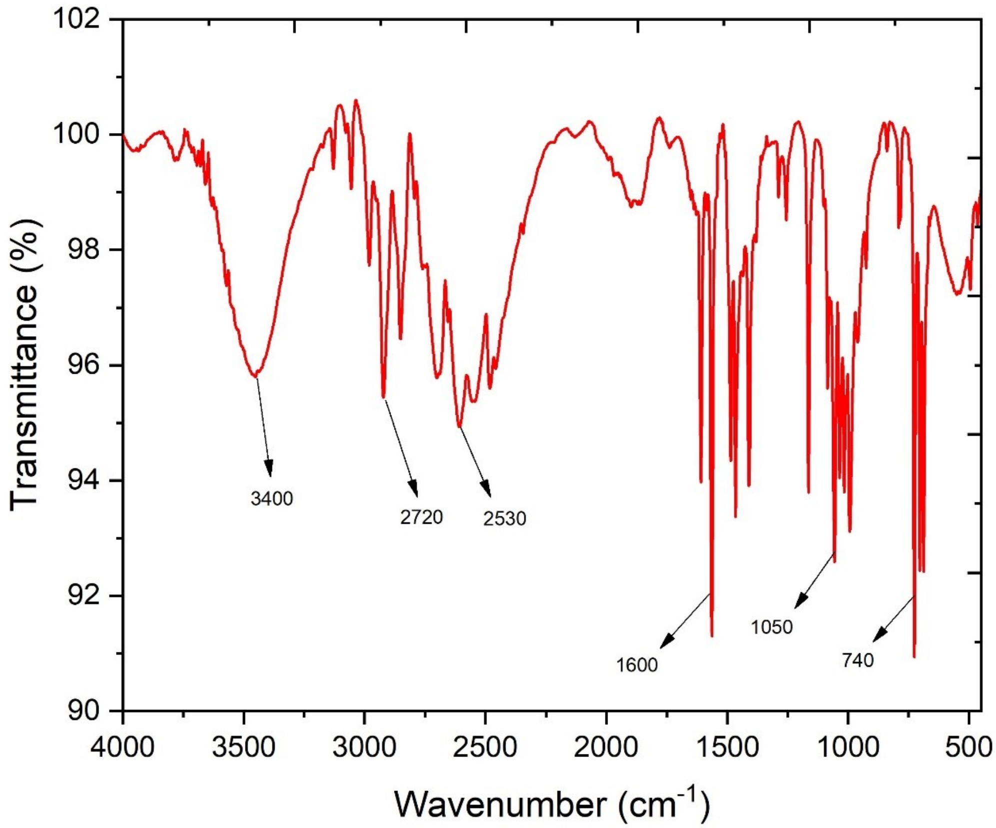

^{1}H NMR of 5-(2-chloro-6-fluorophenyl)-1H-tetrazole (5h)

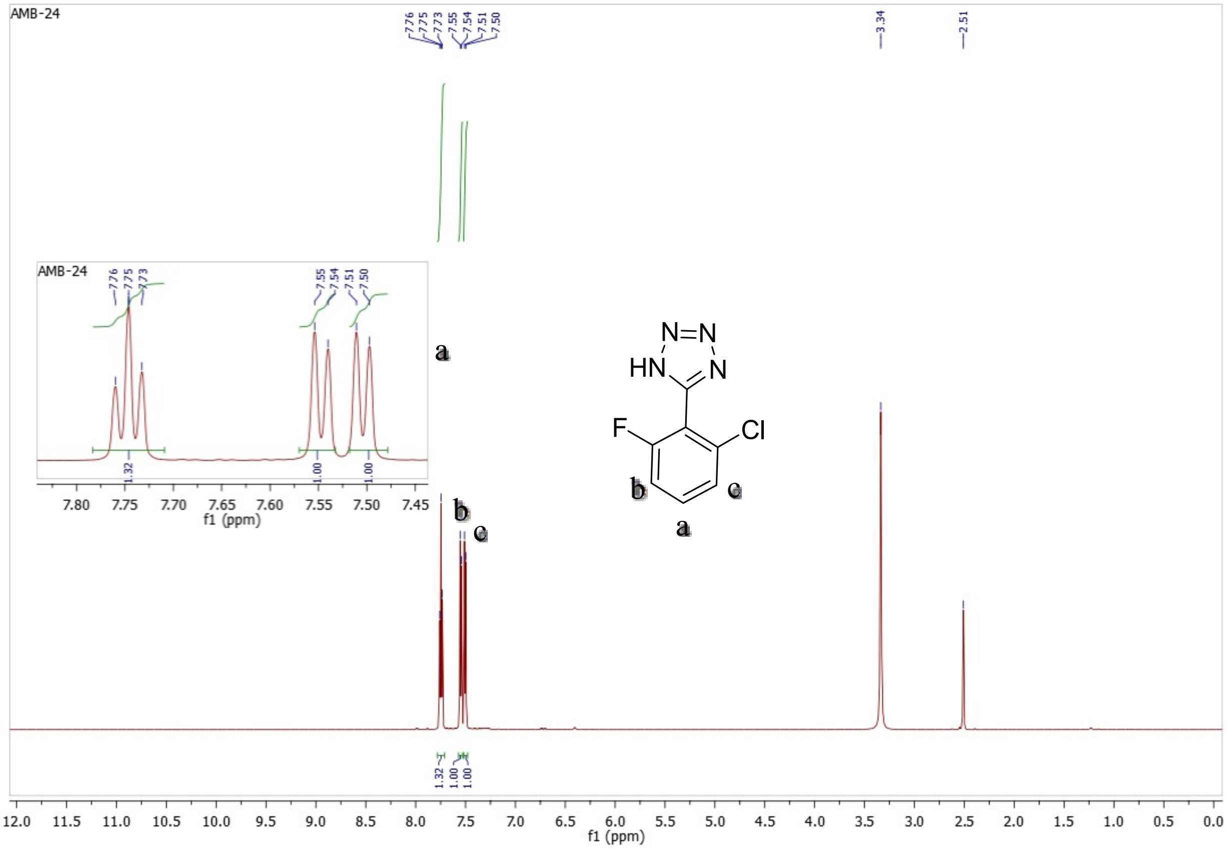

^{13}C NMR of 5-(2-chloro-6-fluorophenyl)-1H-tetrazole (5h)

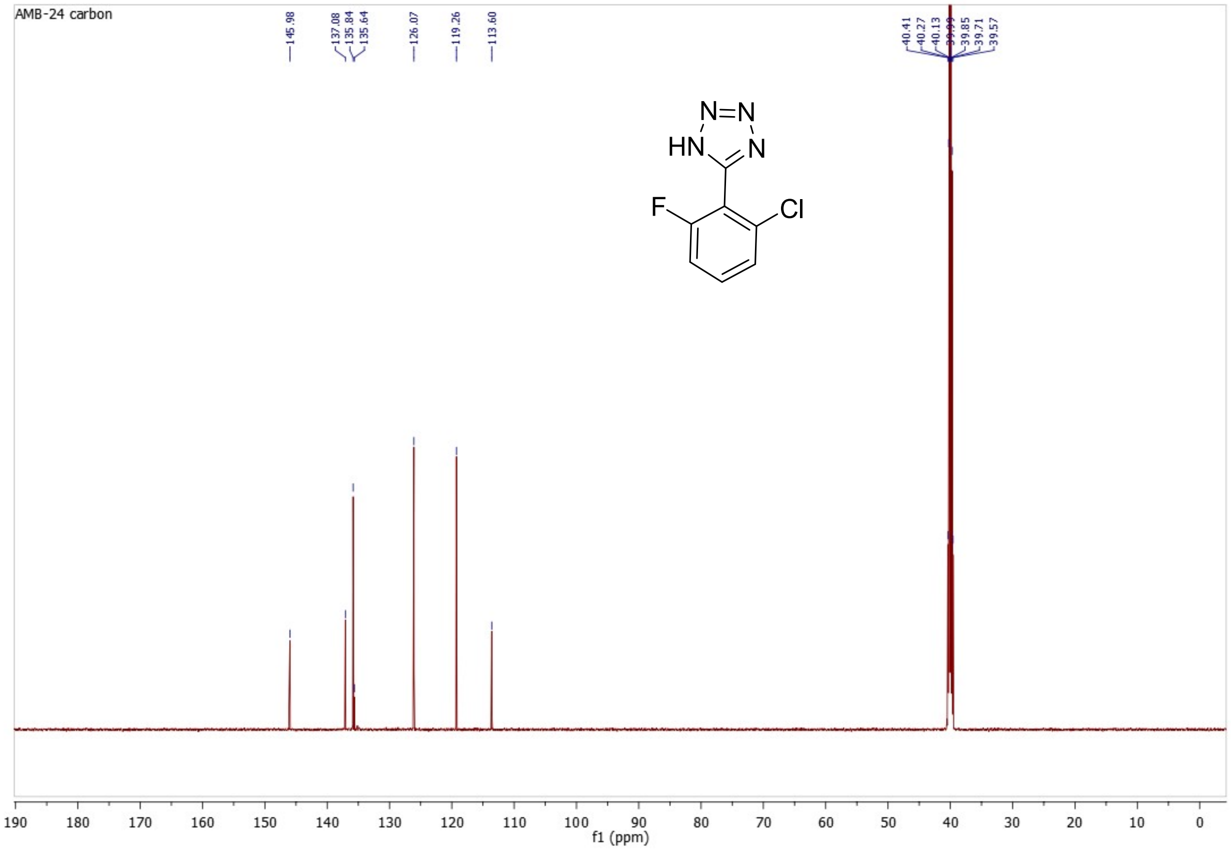

FT-IR of 5-(2-chloro-6-fluorophenyl)-1H-tetrazole (5h)

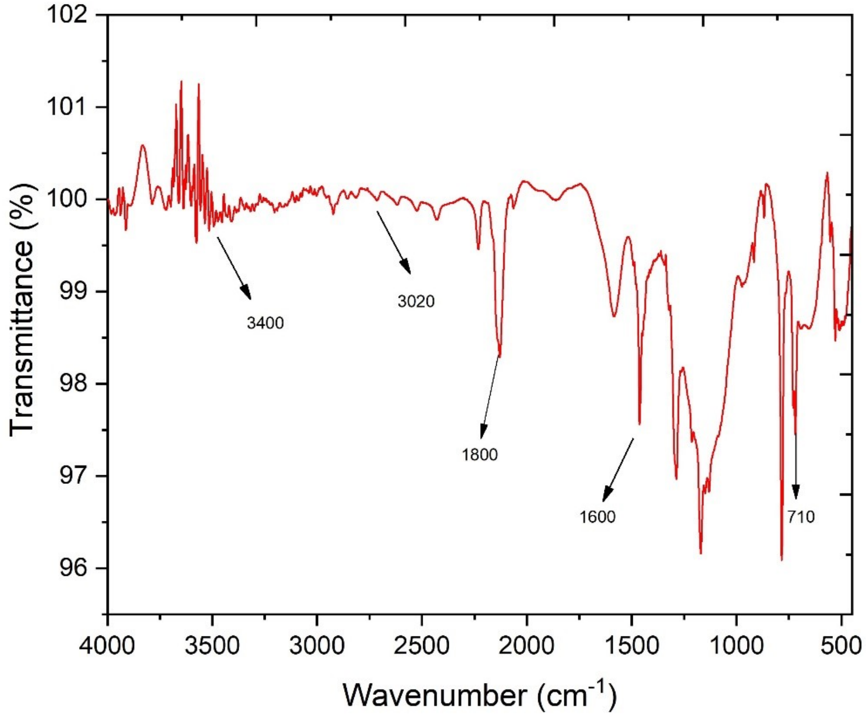

^{1}H NMR of 5-(3-fluorophenyl)-1H-tetrazole (5i)

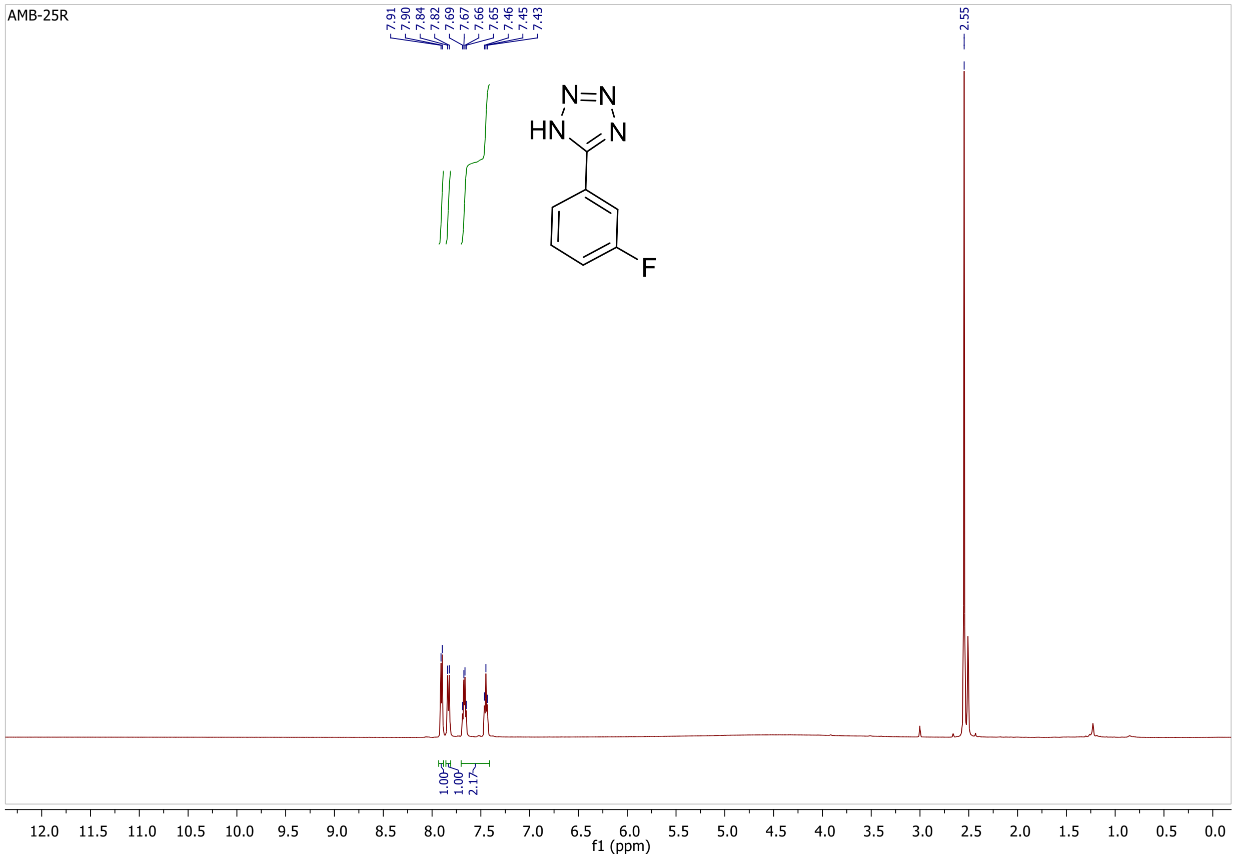

^{13}C NMR of 5-(3-fluorophenyl)-1H-tetrazole (5i)

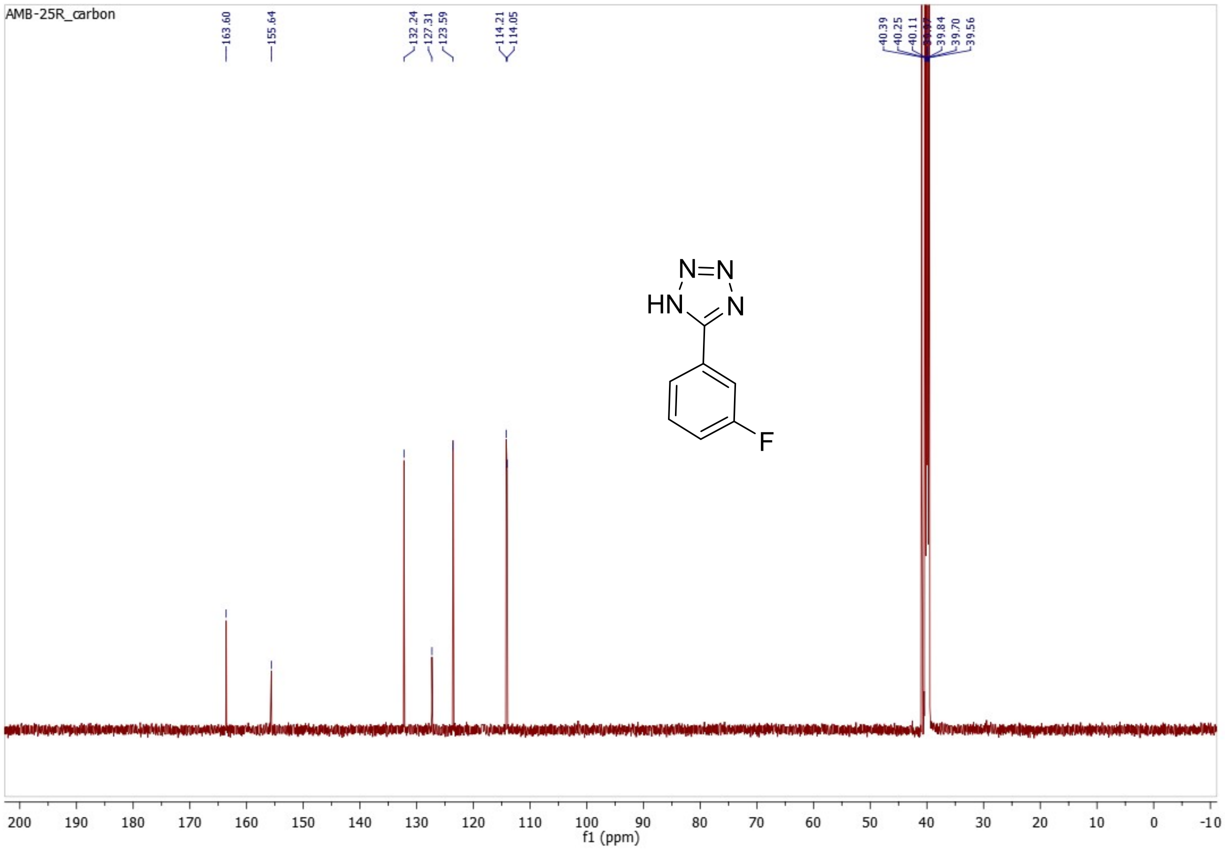

FT-IR of 5-(3-fluorophenyl)-1H-tetrazole (5i)

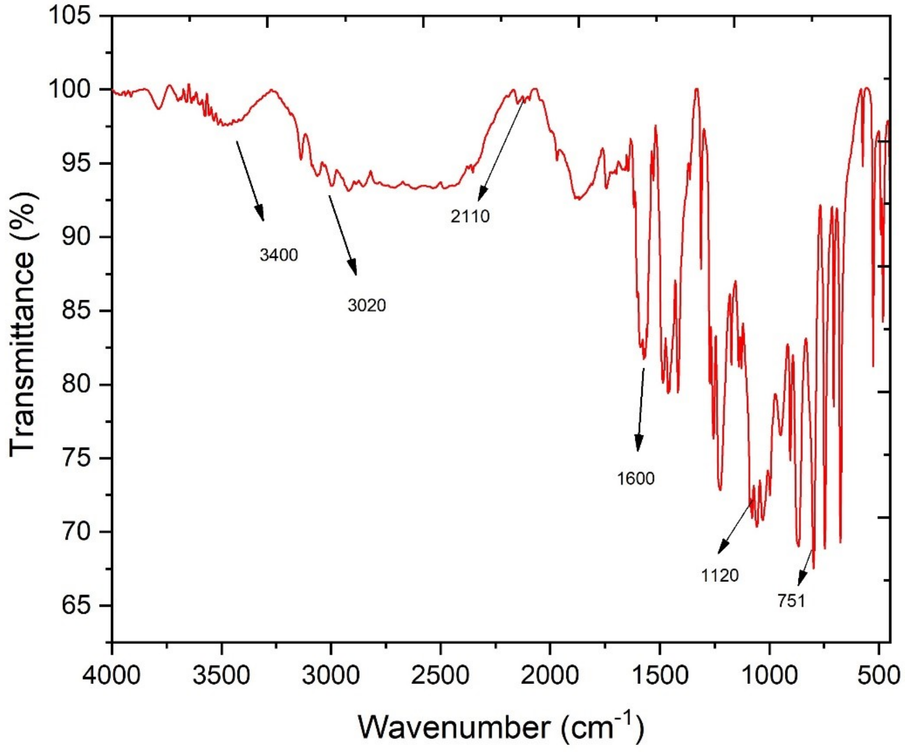

^{1}H NMR of 2-chloro-4-(1H-tetrazol-5-yl)pyridine (5j)

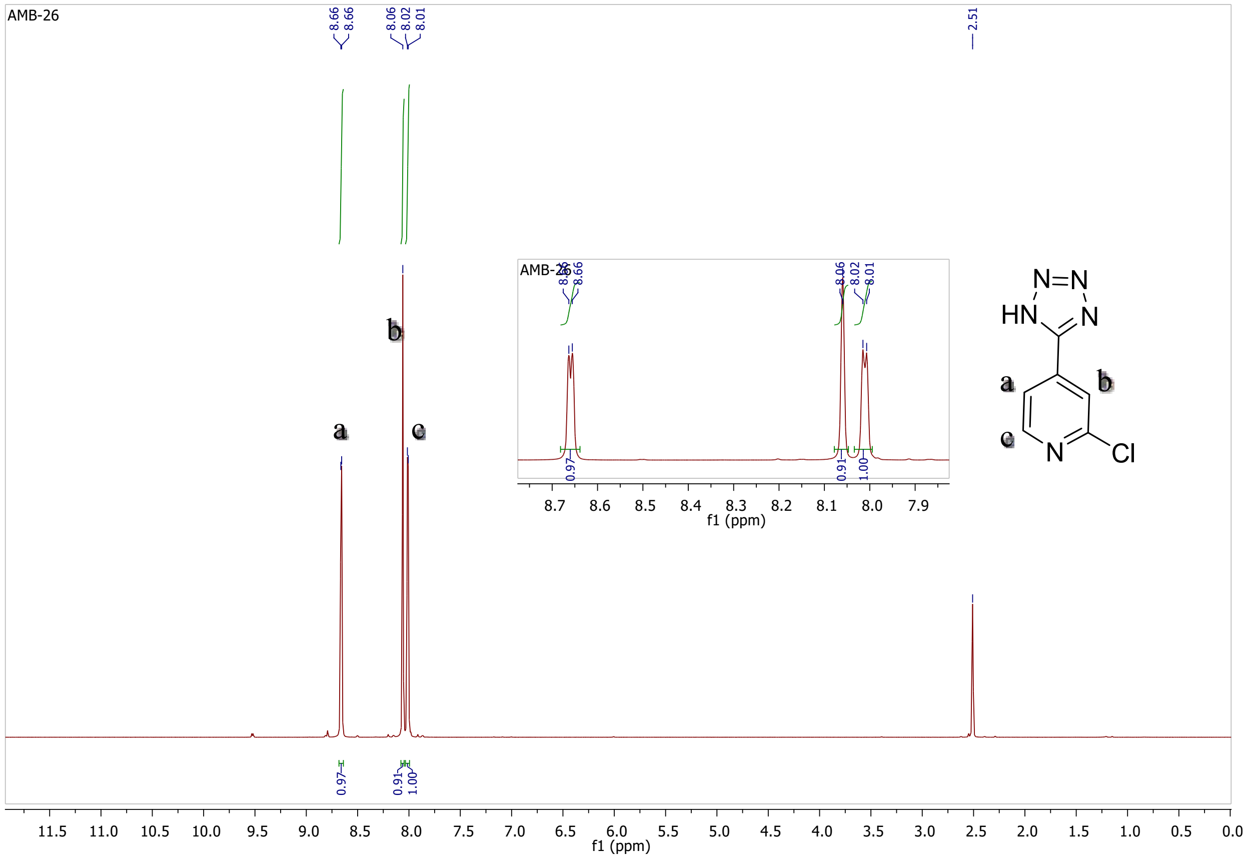

^{13}C NMR of 2-chloro-4-(1H-tetrazol-5-yl)pyridine (5j)

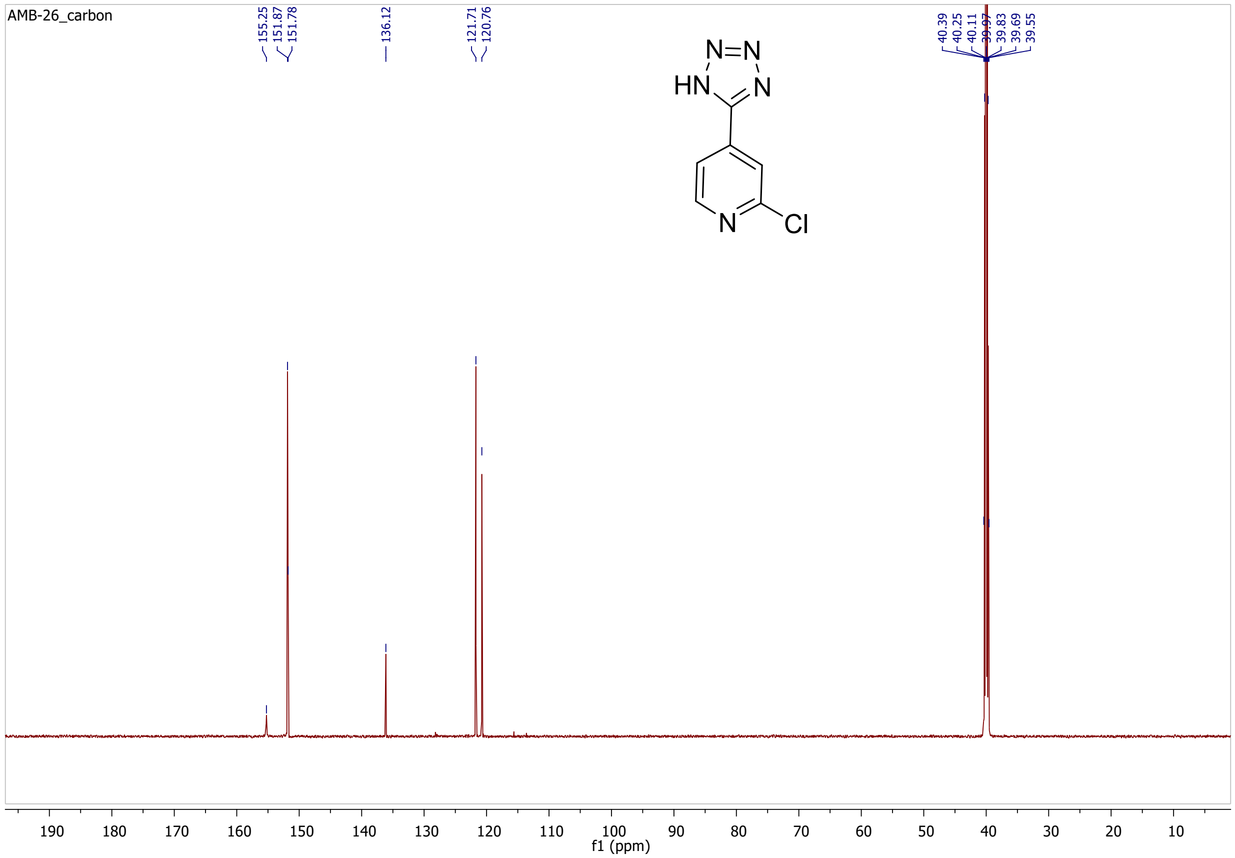

FT-IR of 2-chloro-4-(1H-tetrazol-5-yl)pyridine (5j)

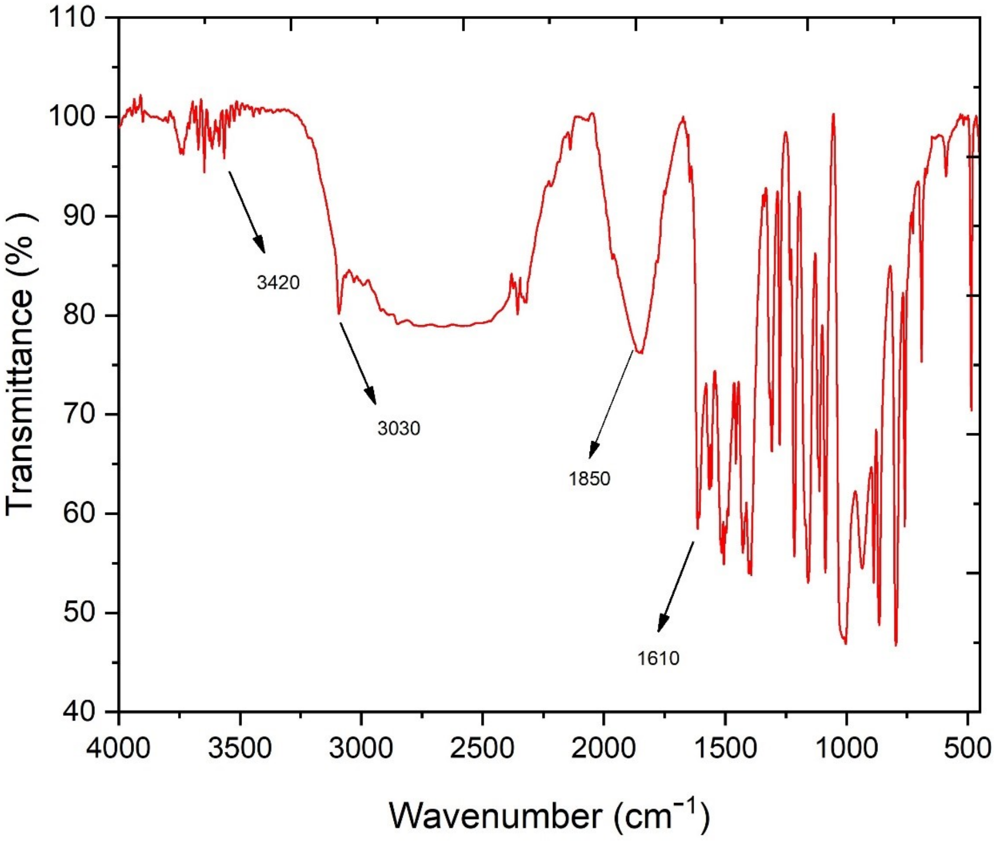

^{1}H NMR of 5-(4-bromophenyl)-1H-tetrazole (5k)

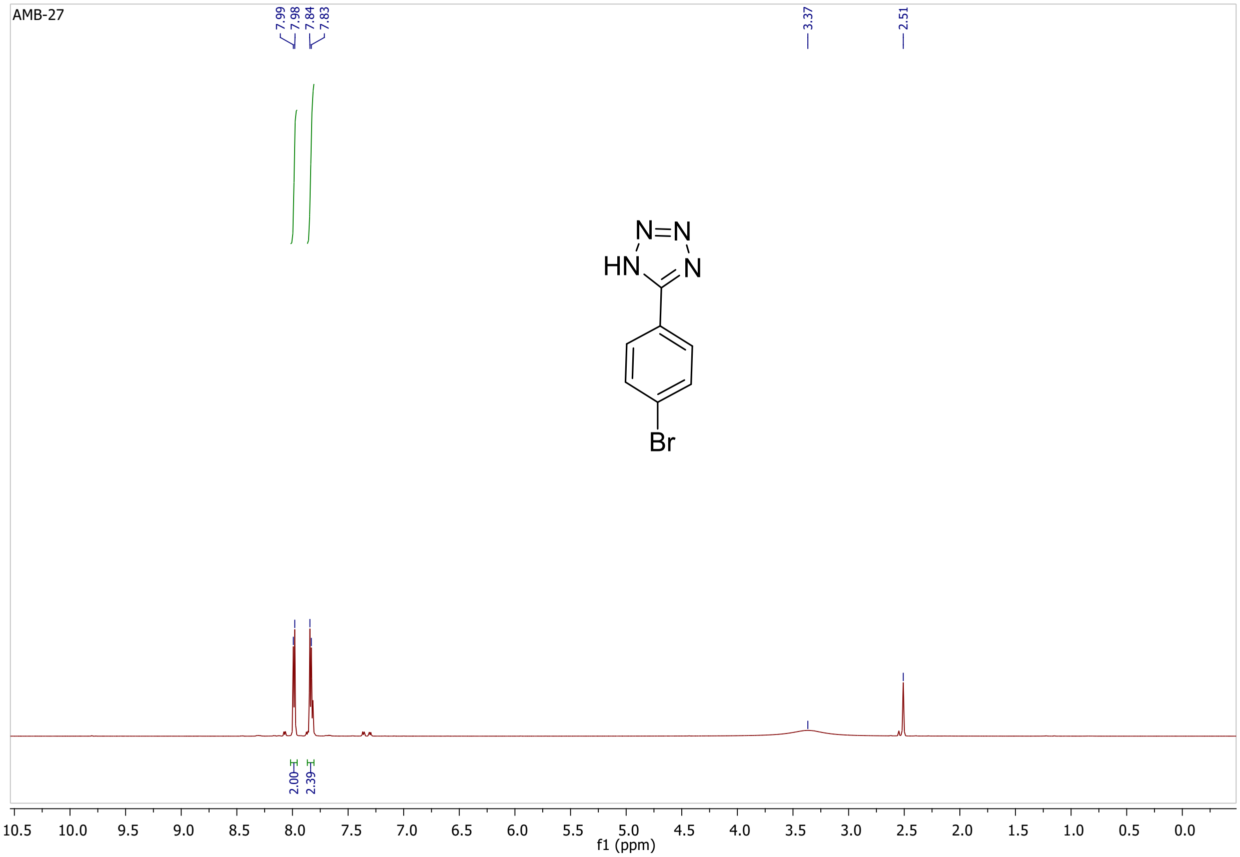

^{13}C NMR of 5-(4-bromophenyl)-1H-tetrazole (5k)

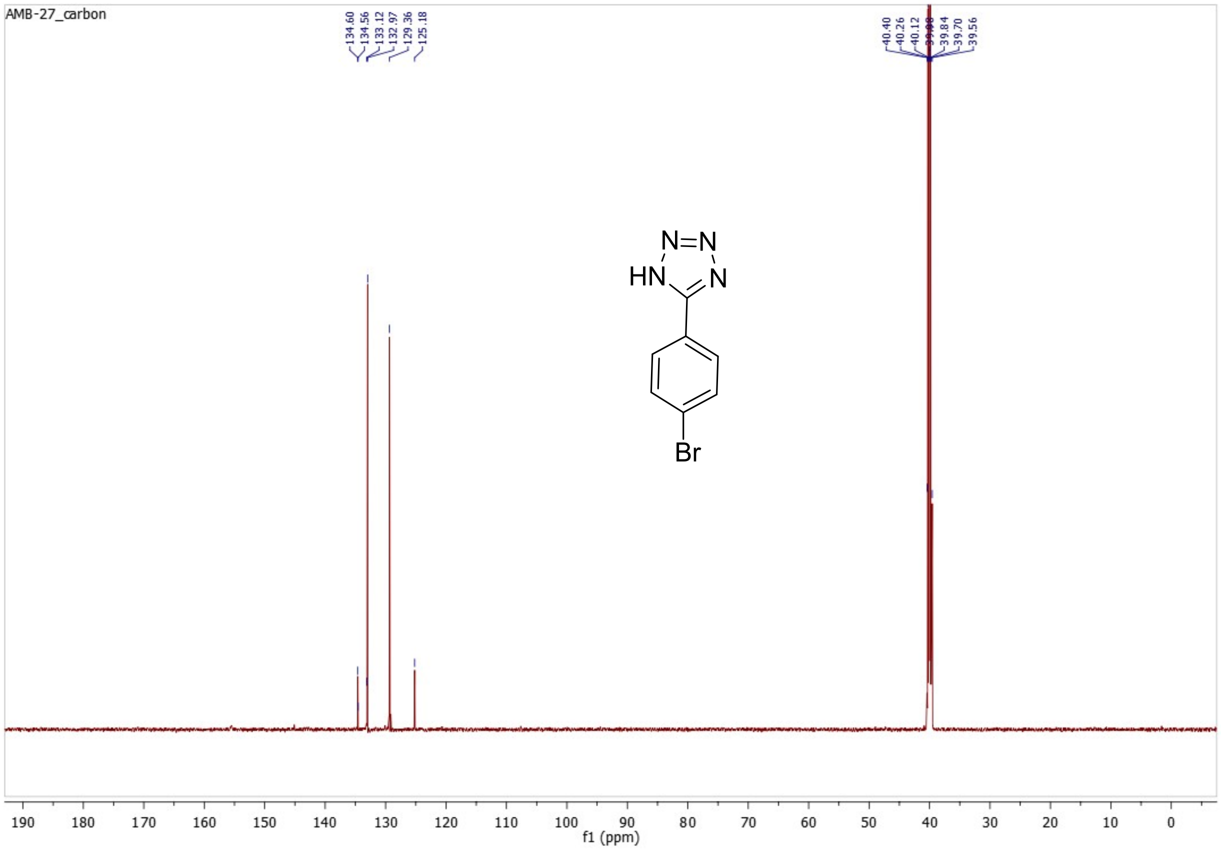

FT-IR of 5-(4-bromophenyl)-1H-tetrazole (5k)

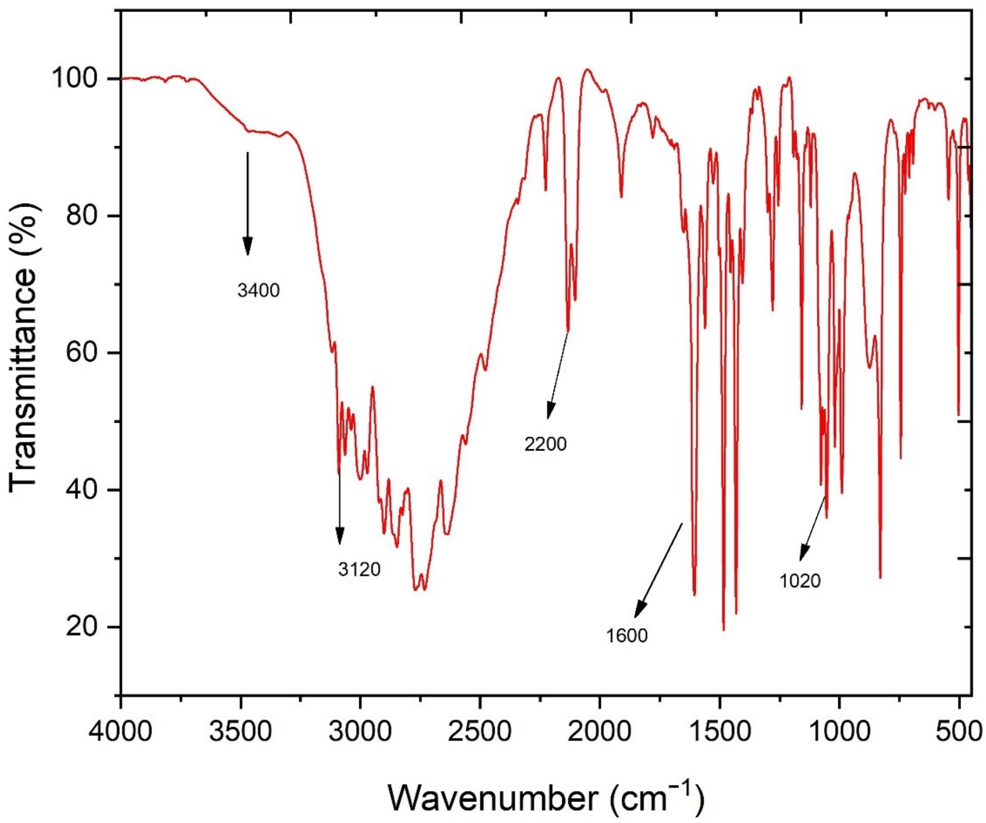

^{1}H NMR of 2-bromo-4-(1H-tetrazol-5-yl)phenol (5l)

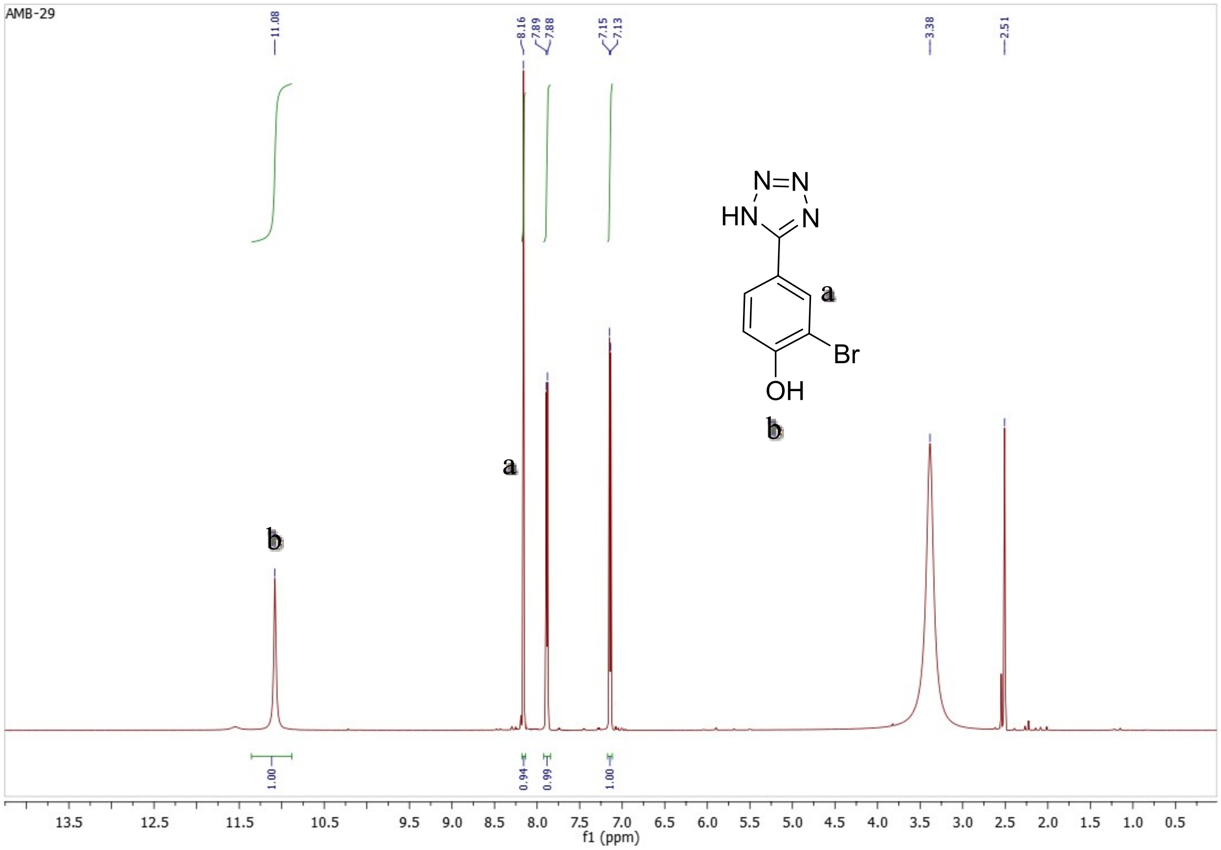

^{13}C NMR of 2-bromo-4-(1H-tetrazol-5-yl)phenol (5l)

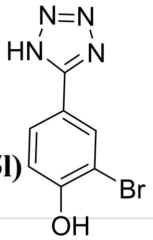

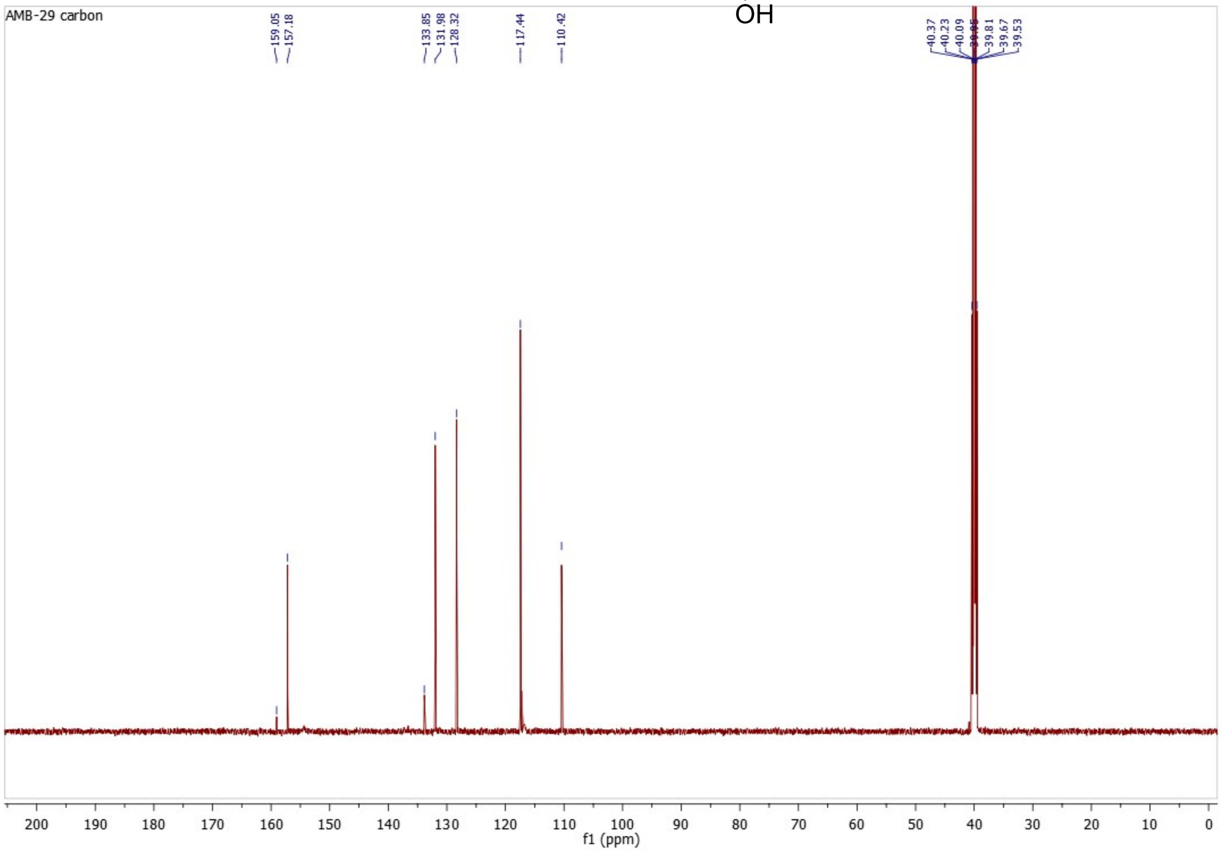

HRMS of 2-bromo-4-(1H-tetrazol-5-yl)phenol (5l)

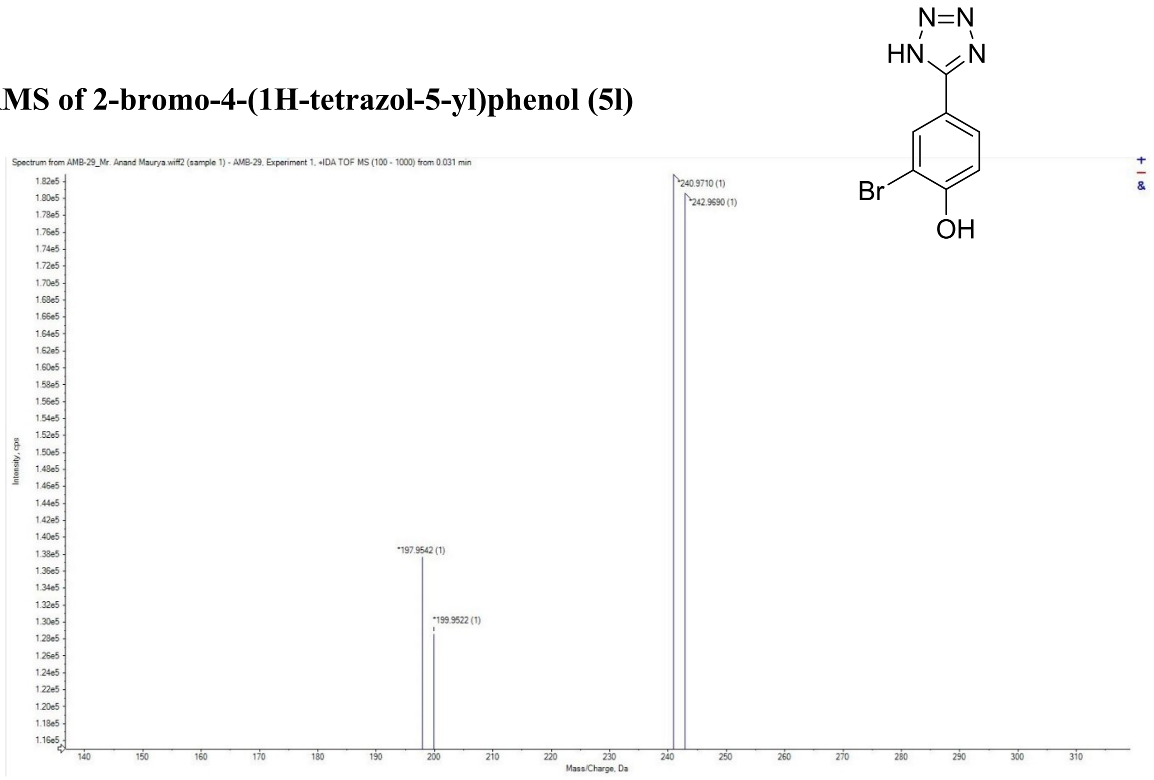

FT-IR of 2-bromo-4-(1H-tetrazol-5-yl)phenol (5l)

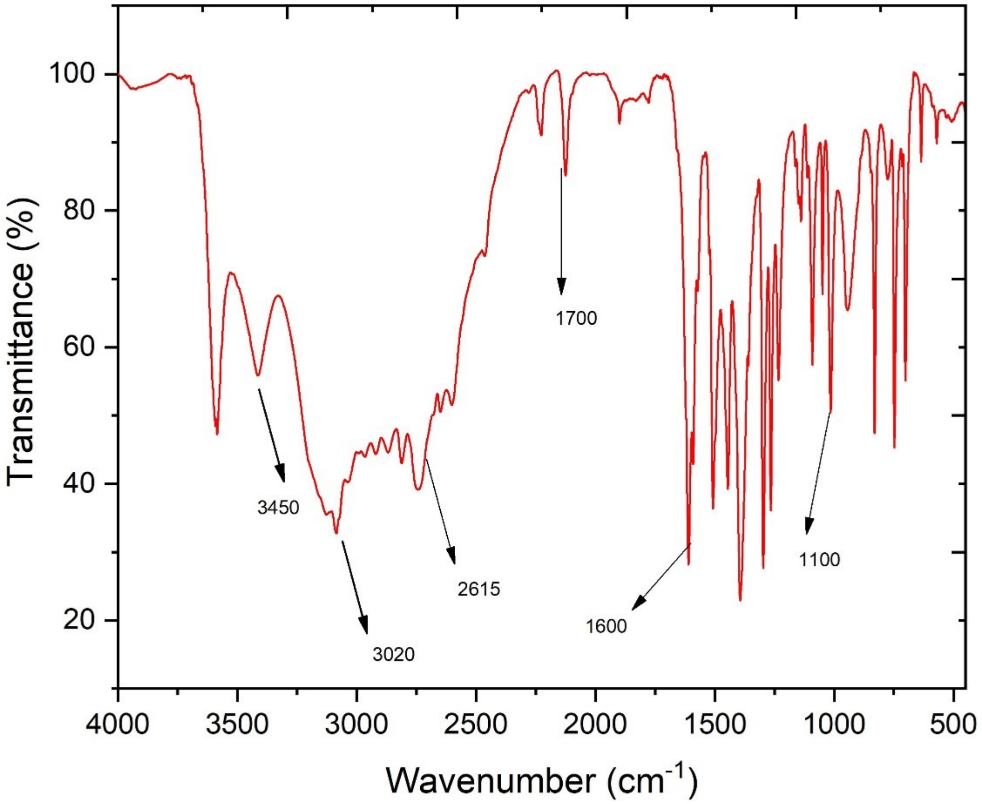

^{1}H NMR of 5-(p-tolyl)-1H-tetrazole (5m)

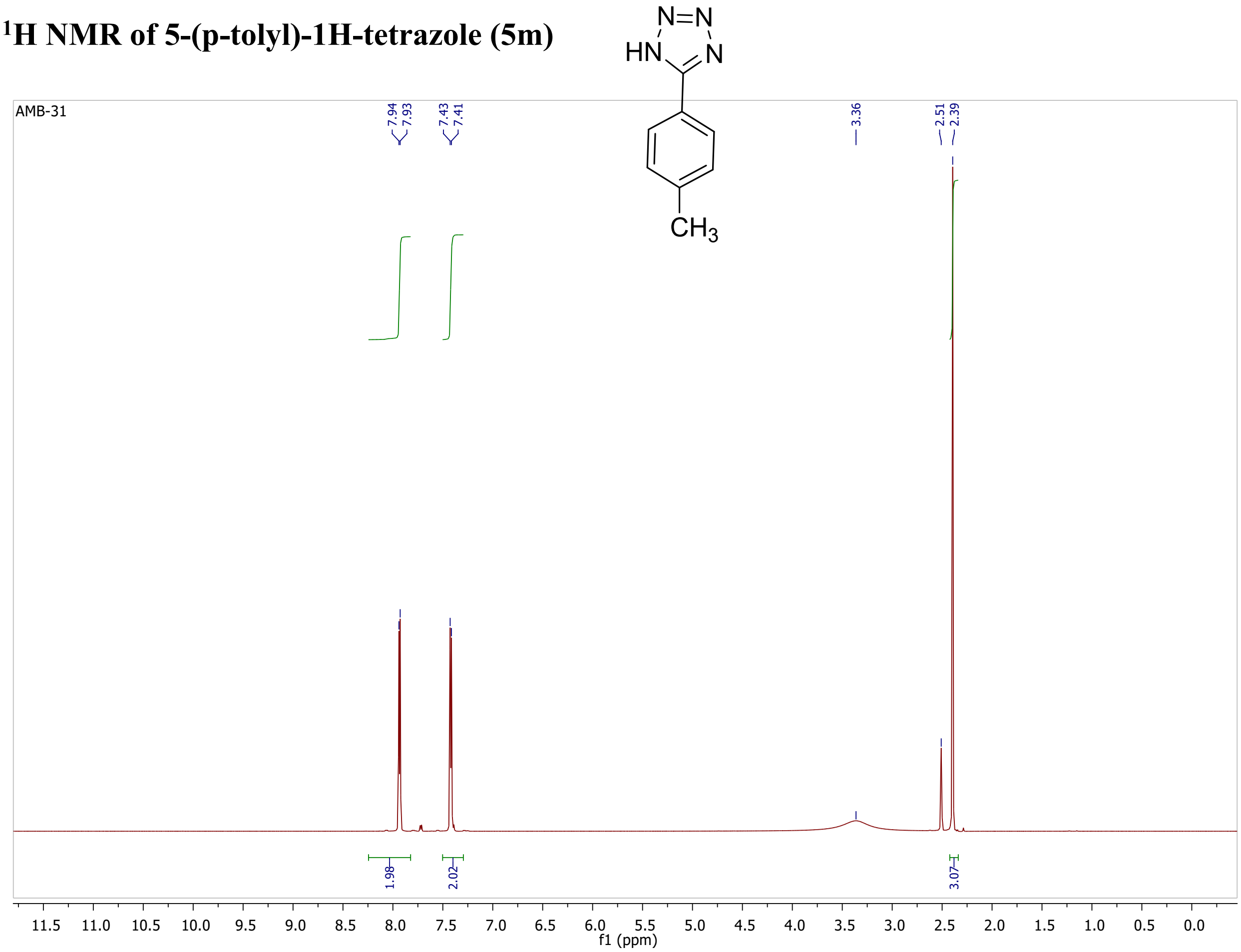

^{13}C NMR of 5-(p-tolyl)-1H-tetrazole (5m)

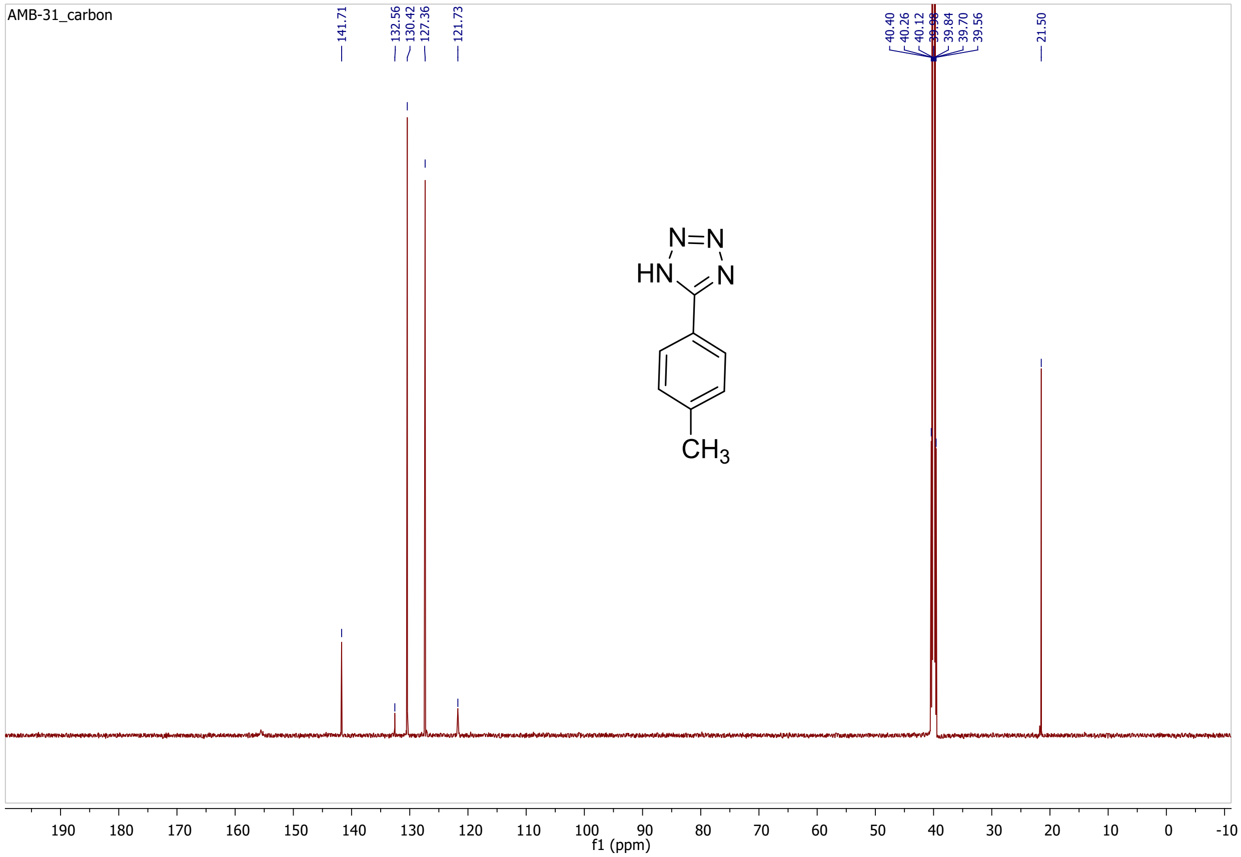

FT-IR of 5-(p-tolyl)-1H-tetrazole (5m)

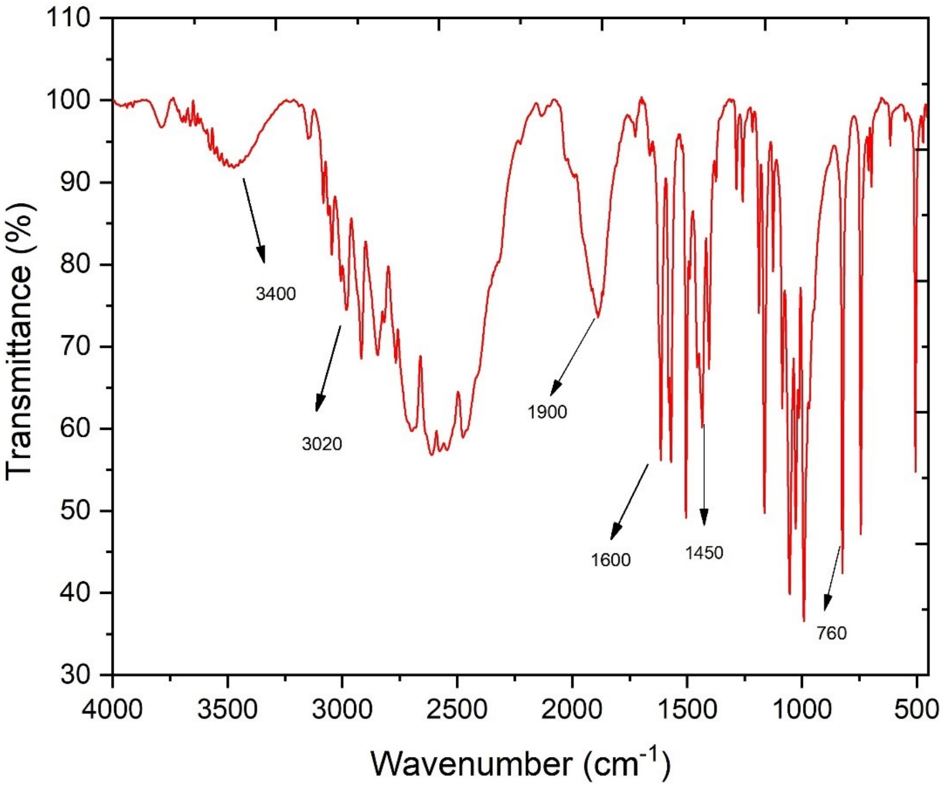

^{1}H NMR of 2-chloro-3-(1H-tetrazol-5-yl)pyridine (5n)

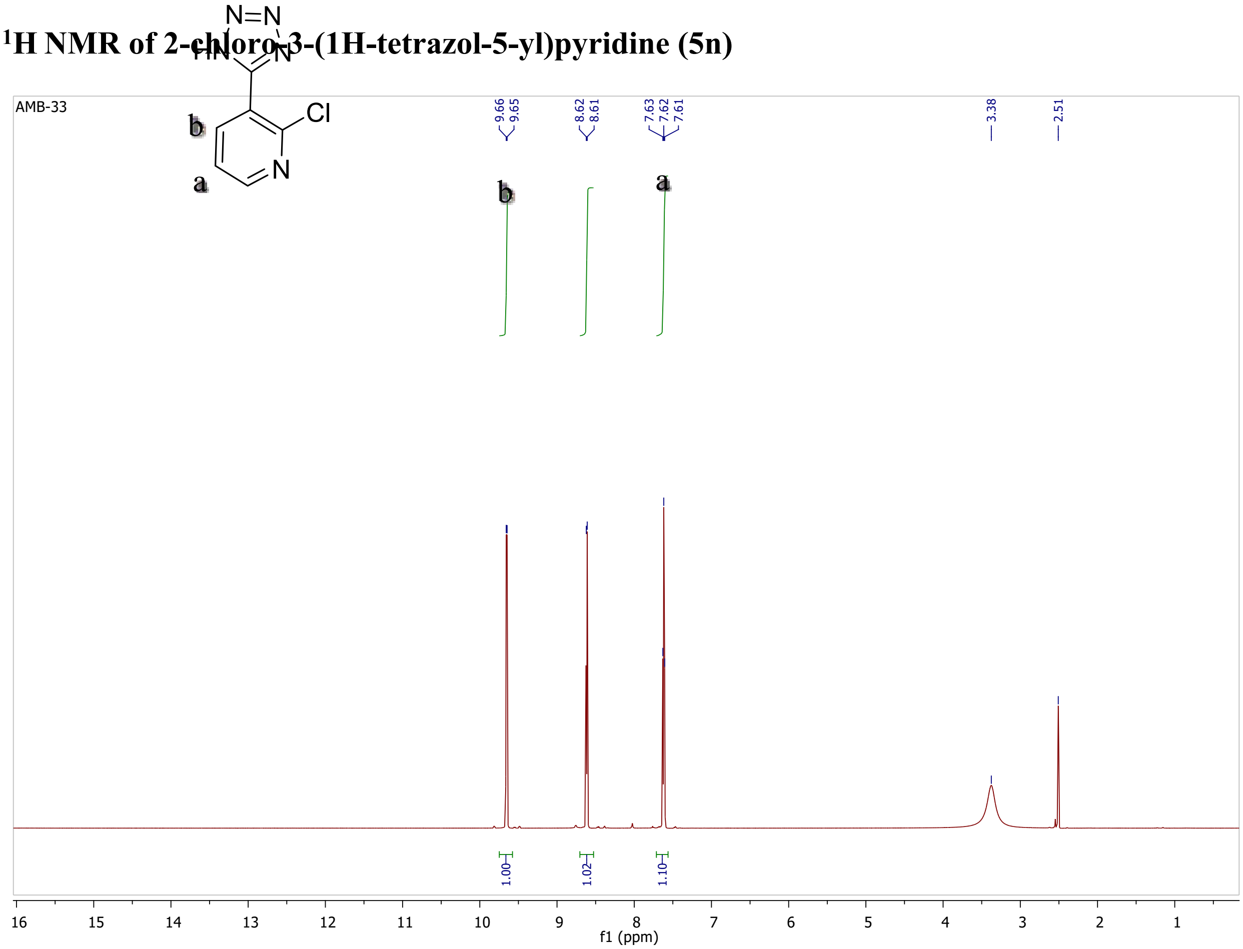

^{13}C NMR of 2-chloro-3-(1H-tetrazol-5-yl)pyridine (5n)

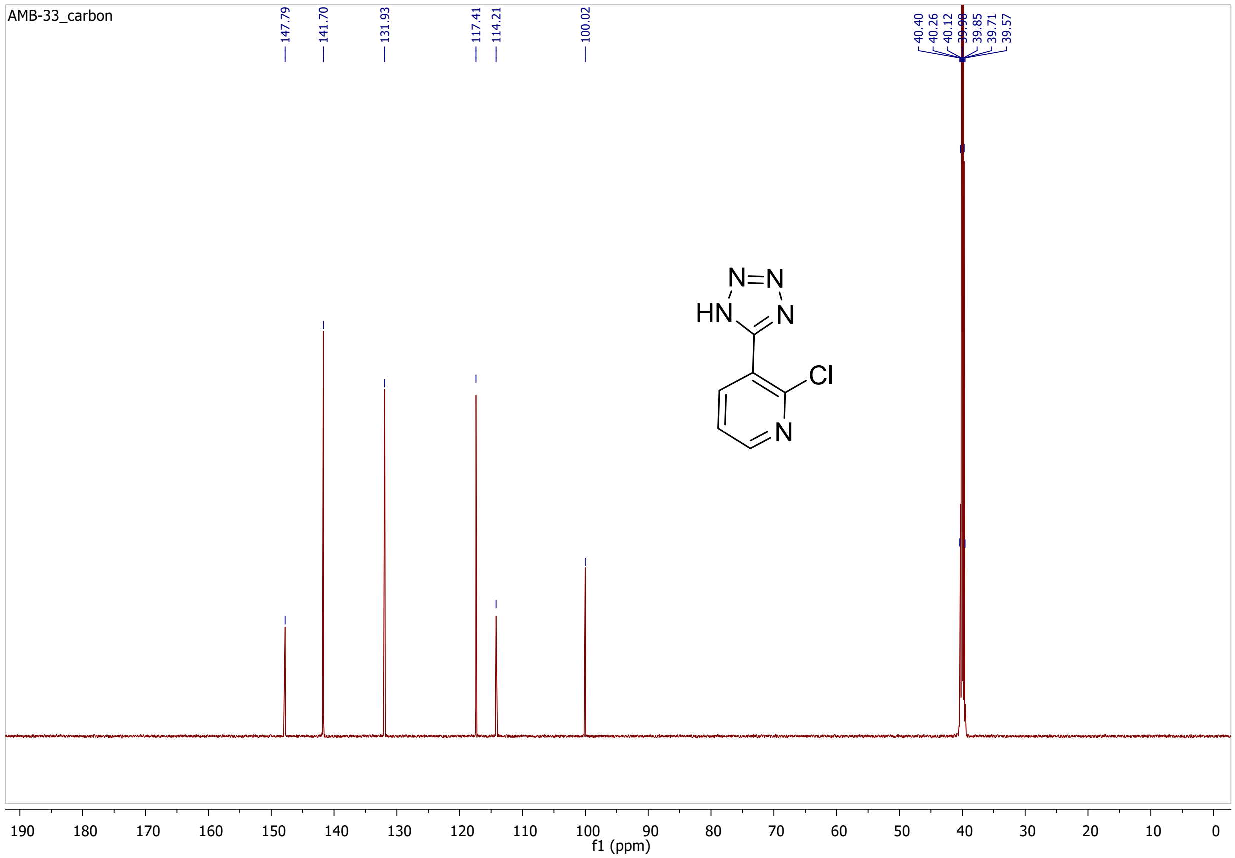

FT-IR of 2-chloro-3-(1H-tetrazol-5-yl)pyridine (5n)

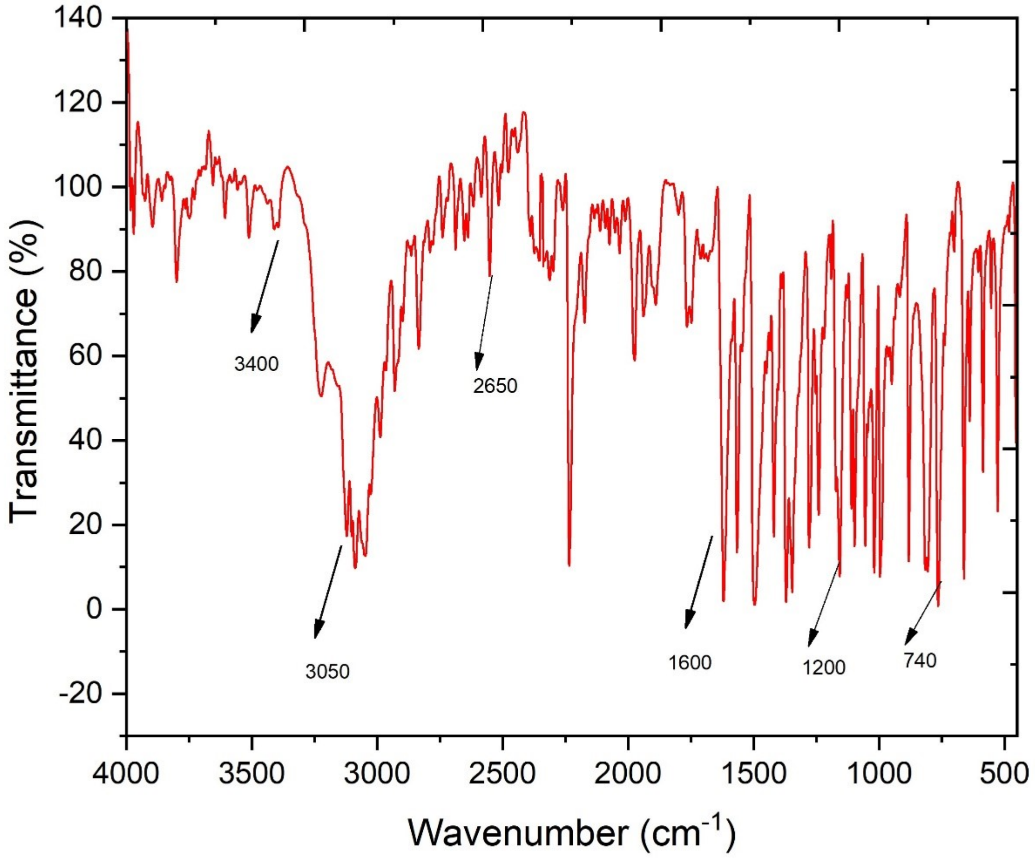

^{1}H NMR of 4-(1H-tetrazol-5-yl)benzaldehyde (5o)

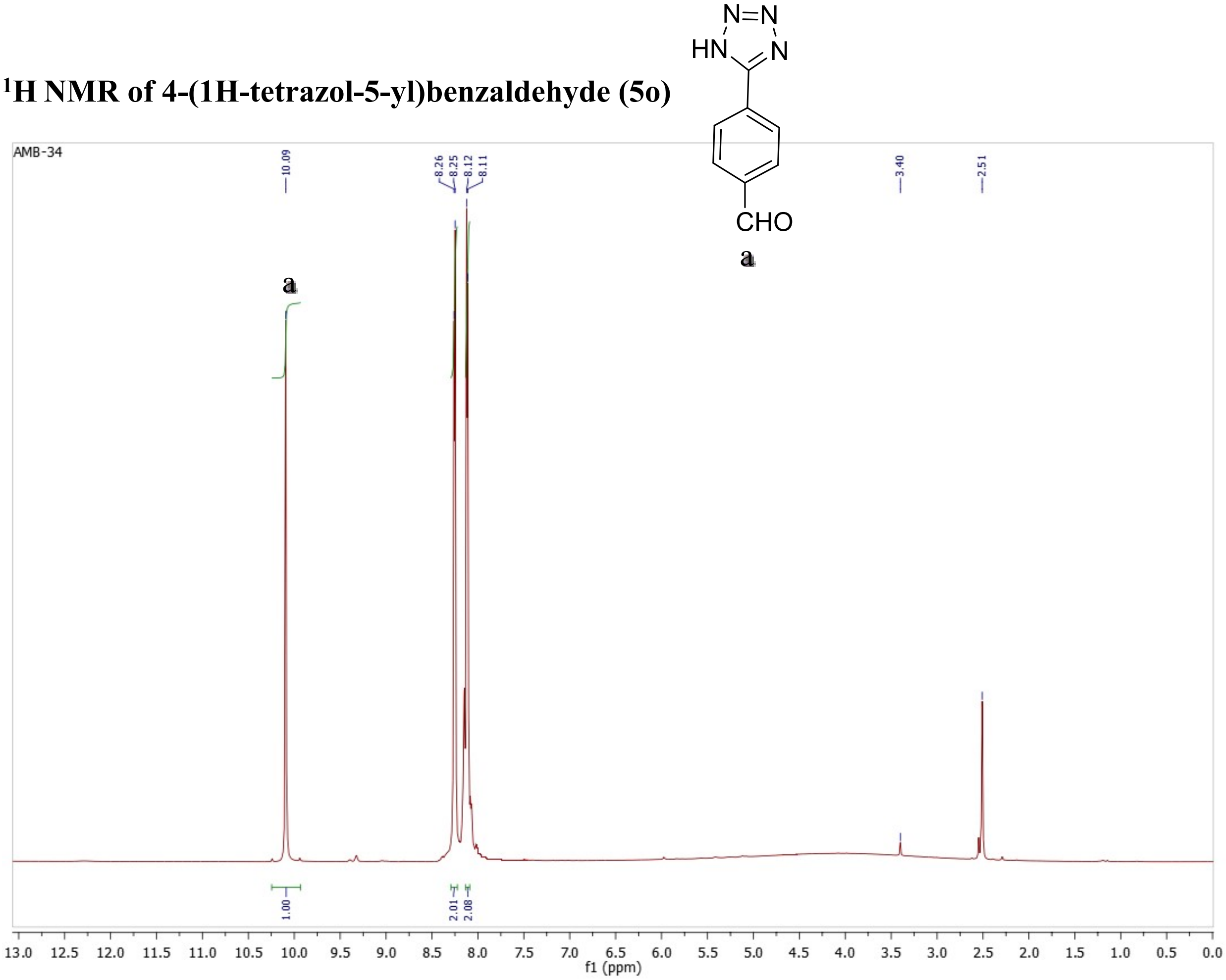

^{13}C NMR of 4-(1H-tetrazol-5-yl)benzaldehyde (5o)

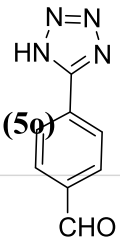

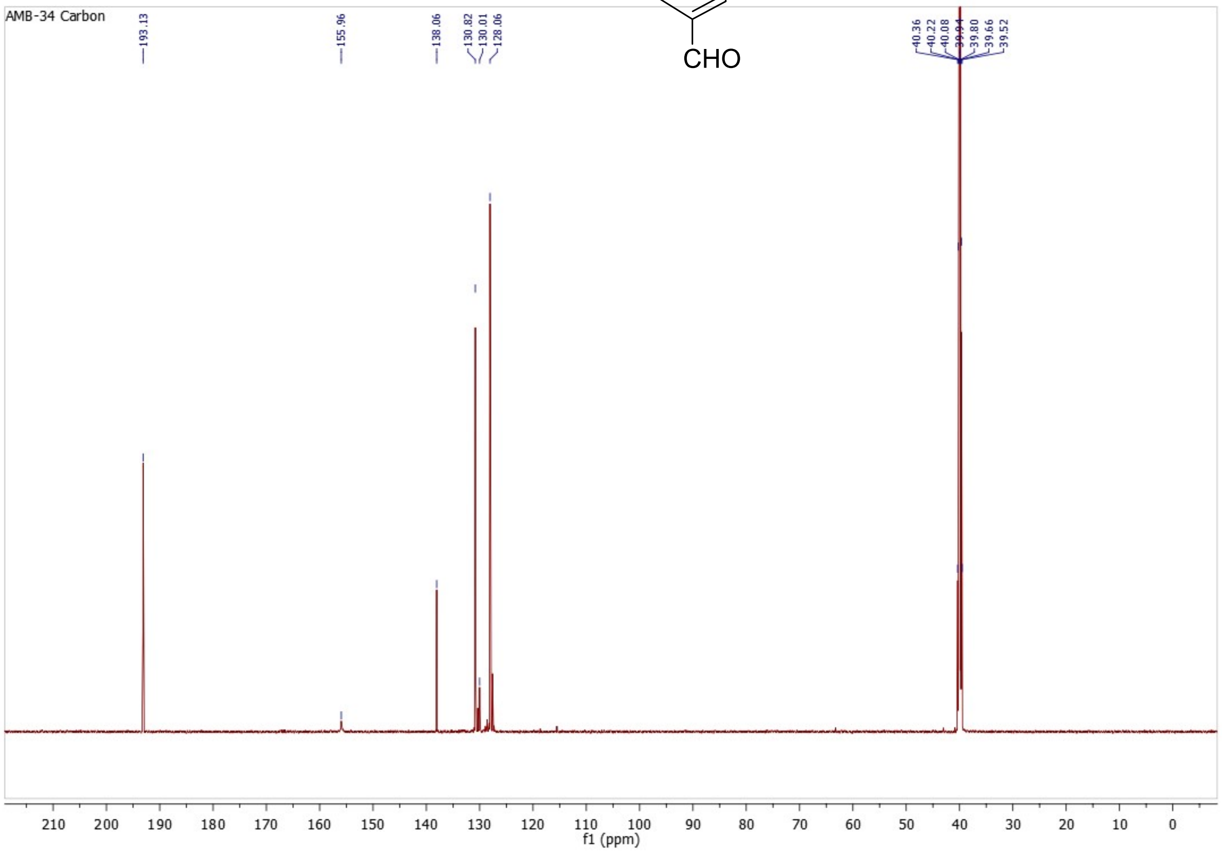

HRMS of 4-(1H-tetrazol-5-yl)benzaldehyde (5o)

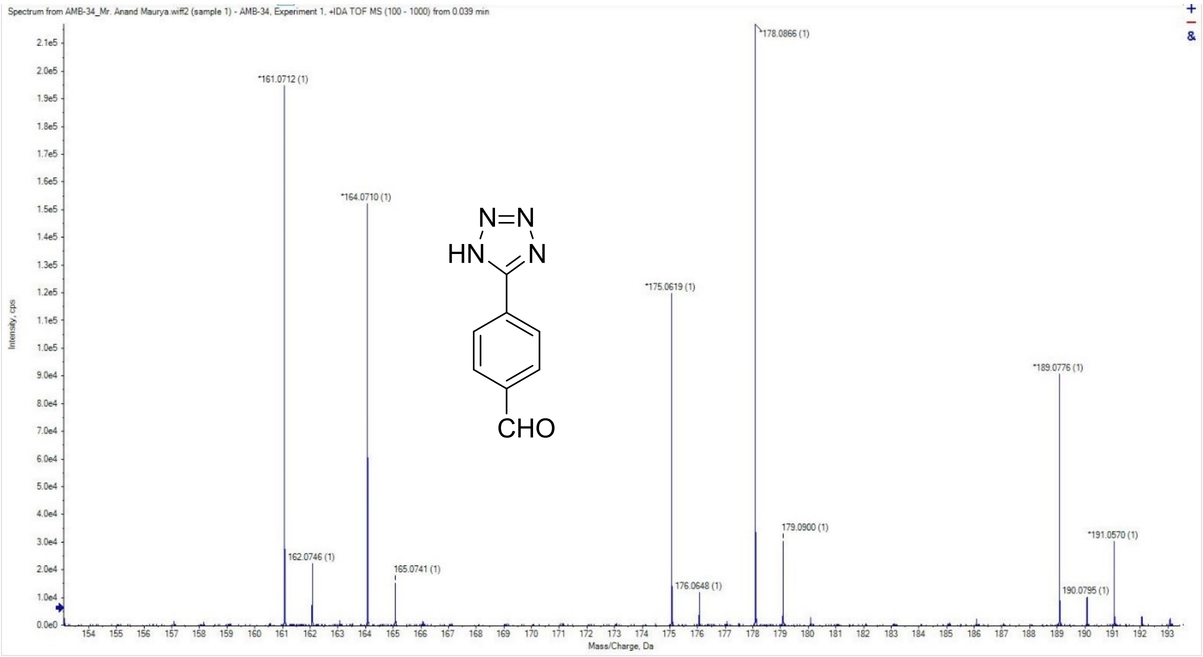

FT-IR of 4-(1H-tetrazol-5-yl)benzaldehyde (5o)

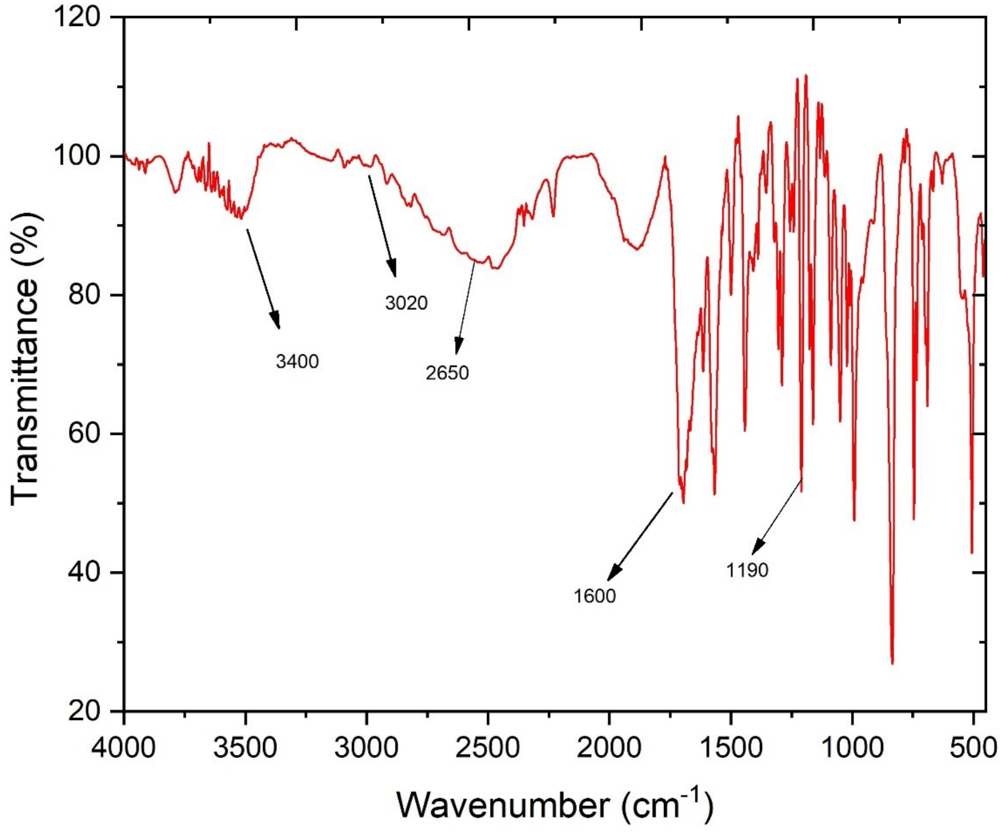

6. Physical data of 5-phenyl-1*H*-tetrazole and 4-chloroaniline after the seventh cycle.

^{1}H NMR of 5-phenyl-1*H*-tetrazole

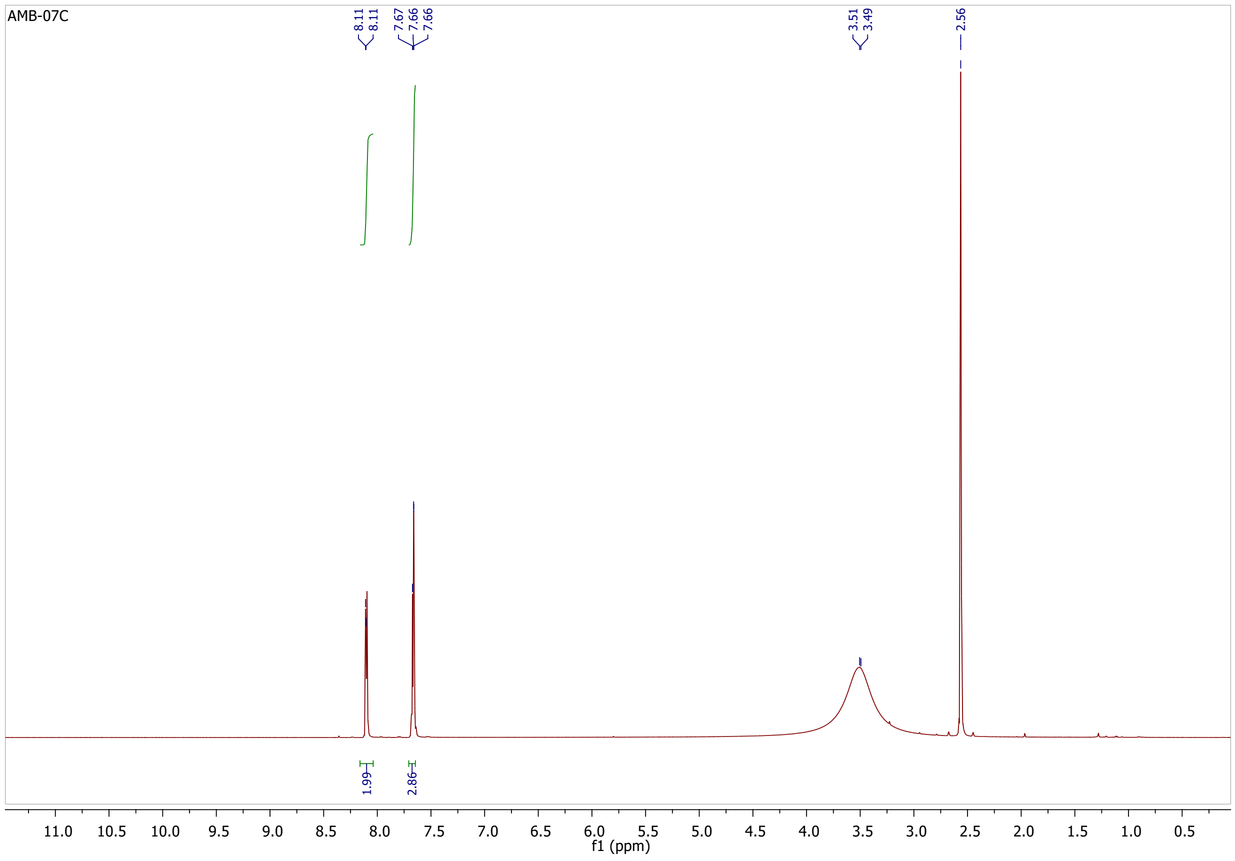

^{1}H NMR of 4-chloroaniline

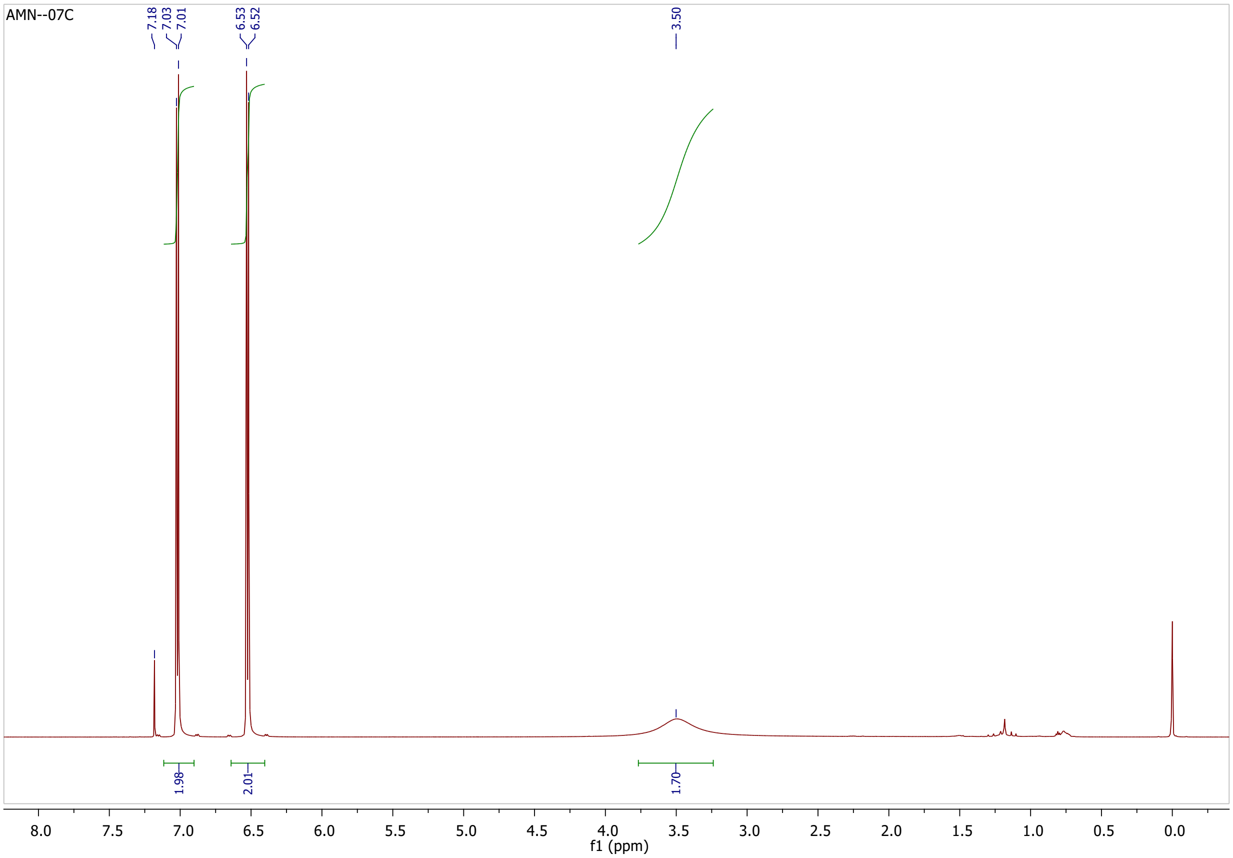

Supplement: RA-014-D4RA05443A-s001 [file RA-014-D4RA05443A-s001.pdf]
